# Substituents Regulate the Cyclization of Conjugated Alkynes to Accurately Construct Cyclo-(*E*)-[3]dendralenes

**DOI:** 10.3390/molecules28114382

**Published:** 2023-05-27

**Authors:** Yun-Tao Xia, Ya-Xin Li, Tong-Tong Bi, Wei Lian, Xia Wang, Meng Yan, Tao Guo

**Affiliations:** College of Chemistry & Chemical Engineering, Henan University of Technology, Academician Workstation for Natural Medicinal Chemistry of Henan Province, Zhengzhou 450001, China; yaxinli@haut.edu.cn (Y.-X.L.); 202013030321@stu.haut.edu.cn (T.-T.B.); lian18790530028@163.com (W.L.); a18838926005@163.com (X.W.); taoguo@haut.edu.cn (T.G.)

**Keywords:** substituents regulate, conjugated alkynes, cyclization, cyclo-[3]dendralenes

## Abstract

Substituent-regulated cyclization of conjugated alkynes with acid catalysis was developed in this paper, and it provides a straightforward synthesis of cyclic-(*E*)-[3]dendralenes. Depending on the electronic effect of the aromatic ring pairing, a variety of phosphinyl quintuplet/hexa cyclo-[3]dendralenes with diverse substitution patterns are accessible, with good efficiency and high stereoselectivity. This self-cyclization process achieves the first precise construction of a phosphinylcyclo-(*E*)-[3]dendralene from conjugated alkynes to aromatization.

## 1. Introduction

**Scheme 1:**
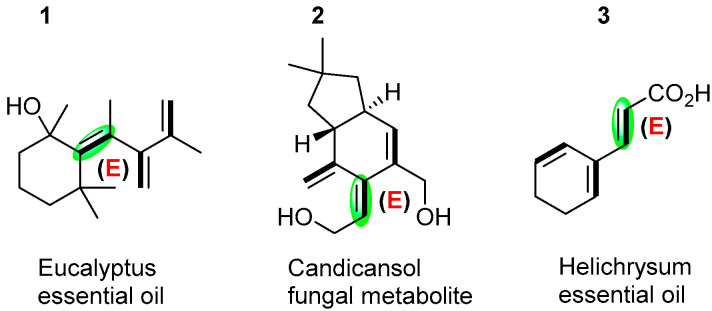
Naturally occurring cyclo-(*E*)-[3]dendralenes.

Dendralenes, commonly known as cross-conjugated oligoalkenes, are an important class of oligo-olefinic hydrocarbon structures, exclusively comprising sp^2^-hybridized carbons [1]. Until the turn of the century, dendralenes had received little attention, most likely due to the erroneous assumption that they were too unstable to be handled in the laboratory using standard equipment and methods [2,3]. These formations were difficult to synthesize for a long time and were believed to be unstable entities from their discovery in 1955, which limited further research. A noticeable breakthrough in the practical synthesis of dendranlenes was reported by Sherburn and co-workers in 2009, finding that dendralenes are actually stable and also have surprising reactivities [4]. Subsequently, dendralenes attracted increased interest because of their unusual structure and electronic phenomenon, with widespread applications of dendralenes in polymer chemistry [5,6], theoretical chemistry [7,8,9,10], materials chemistry [11,12], electrochemistry [13], and synthetic chemistry [14,15,16,17,18,19,20,21,22,23,24,25,26], among which, cyclo-[3]dendralene, as the primary member of the family, has attracted the greatest attention owing to its wide existence in naturally occurring products and engagement in synthetic compounds [27]. For linear dendralenes, the physical and chemical properties of the annulenes are dominated by this parity-dependent behavior. Alternation of melting points in families of compounds with even- and odd-length chains has also been reported, and this is related to the property of the packing of molecules in crystal lattices. In these systems, an alternation in behavior does not extend to any other physical or chemical property [28]. Notably, when all double bonds in a dendralene molecule are in the same plane, the endo-type dendralenes will produce significant site resistance. Single bonds will then be rotated to reduce the site resistance, making the molecule resemble a “propeller” shape. However, cyclic-dendralenes limit the rotation of the single bond, which will affect the arrangement of the double bond and the chemical properties of the molecule [28]. Although cyclic-(*E*)-[3]dendralenes are also important skeletons in natural products, as shown in Scheme 1 [29,30], only a handful of cases have been devoted to synthesizing these stereo-structures, sometimes with unsatisfied stereoselectivity.

**Scheme 2:**
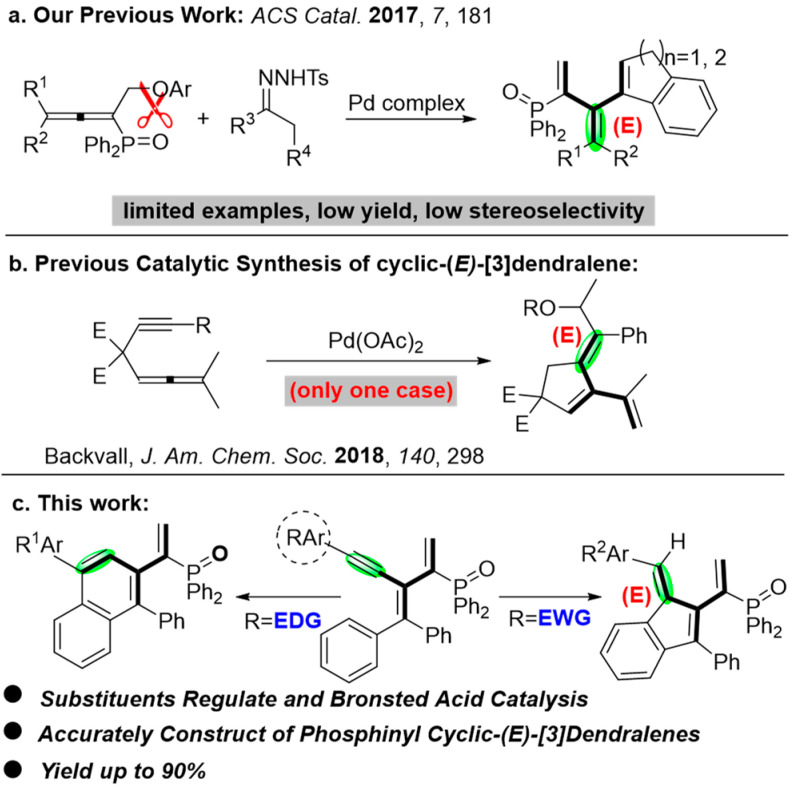
(**a**) Our previous work [29]; (**b**) previous catalytic synthesis of (*E*)-[3]dendralenes [30]; (**c**) this work.

The accurate construction of cyclo-[3]dendralenes is challenging. The palladium-catalyzed coupling of allenynes is an efficient way to obtain these cross-conjugated polyenes. In particular, only two papers documented the construction of cyclic-(*E*)-[3]dendralenes. In 2017, our group disclosed a palladium-catalyzed coupling of allenylphosphine oxide and *N*-tosyl-hydrazone affording diverse phosphinyl cyclic-(*E*)-[3]dendralenes [29]. However, limited examples were successful in acceptable yields and stereoselectivity (Scheme 2a). Later on, Backvall’s group revealed the synthesis of cyclic-(*E*)-[3]dendralenes with palladium-catalyzed oxidative arylating carbocyclization of allenynes, though just one case was reported (Scheme 2b) [30].

## 2. Results and Discussion

Inspired by the aforementioned studies and our continuous work on allenyl-phosphine oxides [31,32,33,34,35,36,37], mechanistically, an ideal approach to access phosphinyl cyclic-(*E*)-[3]dendralenes can be an acid-catalyzed cyclization and substituent-regulated conjugation of alkynes (Scheme 2c).

Initially, phosphinyl-conjugated enynes were prepared according to our previous study, and substrate **1a** was selected as a model. As shown in Table 1, solvents and acids played crucial roles in producing the target phosphinyl cyclic-(*E*)-[3]dendralenes. Successful research indicated that using AgSbF_6_ directly in 1,4-dioxane, the self-cyclization reaction produced phosphinyl cyclic-(*E*)-[3]dendralenes (**2a**) in a 78% yield (entry 3). Interestingly, DCM was proven to be the best choice and enhanced the isolated yield to 90% (entry 1). However, it is accompanied by a small amount of **3a** production. As a comparison, other solvents, including DMF, acetonitrile, and toluene led to negative results. Afterward, systematic screenings of the conditions were further performed using varieties of Lewis acid catalysts (entries 6–11). Although no acid was better than that of trifluoroacetic acid, it is quite clear that weak lewis acids, such as AgCl and AgOAc, rendered the decomposition of starting materials, leading to only trace amounts of the products. The target product **2a** was then achieved in yields of 40% (entry 8) and 61% (entry 9) by changing the optimum conditions and the type of Lewis acid. It is worth mentioning that a small amount of six-membered ring products (**3a**) appeared when the type of catalyst was changed (entry 1, entry 13), indicating that regioselective cyclization reactions might be feasible. The target product can hardly be obtained after reducing the ratios of acid catalysts from 30 to 10 mol% (entry 12). Thus, by using TFA as a promoter in DCM with no additives, an efficient self-cyclization reaction of conjugated enynes was achieved to produce phosphinyl cyclic-(*E*)-[3]dendralenes. Moreover, the X-ray structure of **2a** was obtained, and this confirmed the relative stereochemistry of products and revealed multiple double bonds in [3]dendralenes with a “fan-like” dimensional orientation.

**Scheme 3:**
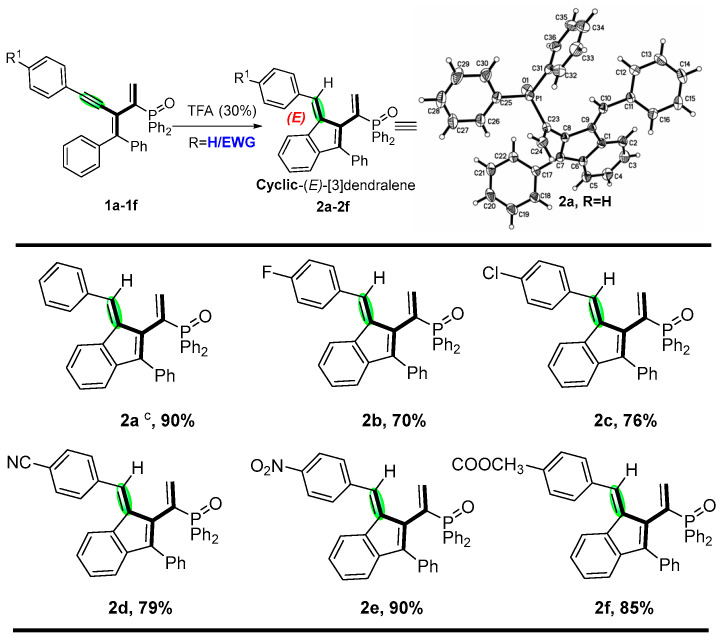
Substrate scope on alkynes ^a,b^. ^a^ Phosphinyl-conjugated enynes (**1a**, 0.1 mmol), catalyst (30 mol%), in 3 mL refluxing solvent for 3 h. ^b^ Isolated yields. ^c^ The stereochemistry was determined using X-ray.

**Scheme 4:**
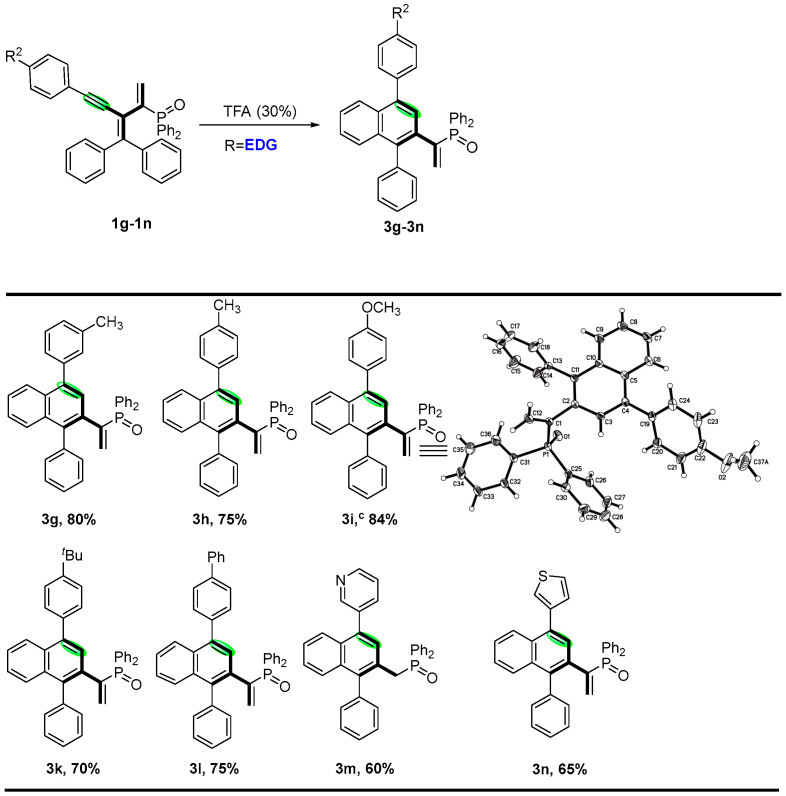
Substrate scope on allenylphosphine oxides and the formation of (*Z*,*Z*)-[3]Dendralenes ^a,b^. ^a^ Phosphinyl-conjugated enynes (**1a**, 0.1 mmol), catalyst (30 mol%), in 3 mL refluxing solvent for 3 h. ^b^ Isolated yields. ^c^ The stereochemistry of **3i** was determined using X-ray.

Encouraged by the preliminary results, we continued to investigate the substrate scope of various phosphinyl-conjugated enynes, and the results are depicted in Scheme 3 and Scheme 4. The stereoselectivity and dimensional orientation of all functional moieties were elucidated by the X-ray structure of **2a** (CCDC 2214047). It is worth mentioning that the electronic effect of the variation in the R^1^ and R^2^ groups would render further stereodiversity to produce cyclic-[3]dendralenes. In general, good to excellent yields of the corresponding cyclic-(*E*)-[3]dendralene products were obtained from para-substituted aryl alkynes containing electron-withdrawing groups, such as fluorine, chlorine, and nitro (**2b**–**e**). In addition, para-substituted aryl alkynes with cyano and ester groups were also tested and, to our delight, were found to be applicable to the system, with good yields ranging from 79 to 85% (**2d** and **2f**). We investigated the substrate scope of enynes, and the results are depicted in Scheme 4. Quite interestingly, phosphinyl-conjugated enynes bearing electron-donating groups, including methyl, methoxy, *t*-butyl, and phenyl at the *para*-positions; methyl at the *meta*-position was well-tolerated in the system, affording the six-membered ring products (**3g**–**3l**) in medium to excellent yields, among which, *para*-methoxy- and *meta-*methyl-derivated conjugated enynes were good candidates as well, and the corresponding products **3g** and **3i** were obtained in 80% and 84% yields. The stereoselectivity was elucidated through the X-ray structure of **3i** (CCDC 2068909). In addition, pyridine-yl and thiophene-yl enynes were also tested, although the conditions needed to be altered and, to our delight, were found to be applicable to the system, with moderate yields (**3m** and **3n**) ranging from 60% to 65% (see Supporting Information (SI) for details).

**Scheme 5:**
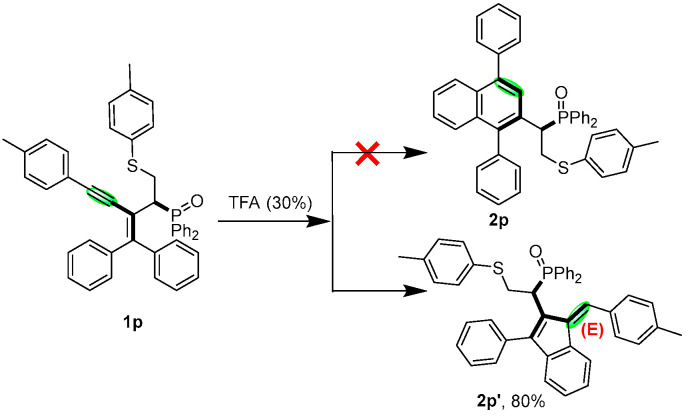
Control experiments.

Finally, in order to study the effect of olefins next to the diphenylphosphine oxide group in this reaction, as shown in Scheme 5, compound **1p** was synthesized and reacted under template conditions. It is noteworthy that these double bonds are crucial for the modulation reaction. If the functional group attached to the phosphorus oxygen is not an olefin, the terminal aryl substituent of the alkyne is not affected by the regulation of this reaction. For example, a substrate of **1g′** does not give the six-membered ring product **2p** according to the previous pattern but only the five-membered ring product **2p′**. This approach offers a novel complementary strategy to synthesize stereodivergent indene skeletal compounds.

**Scheme 6:**
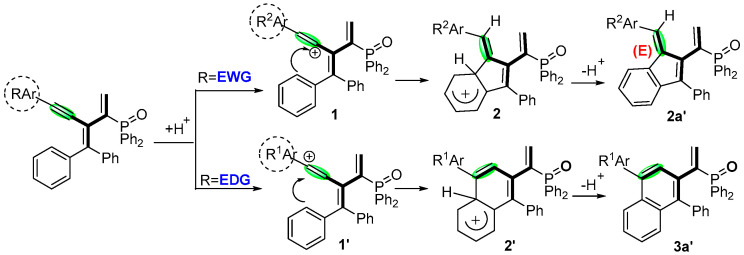
Proposed mechanism.

Based on the acid-catalyzed conjugated alkyne chemistry, self-cyclization process, and previous reports [38], a plausible mechanism is proposed in Scheme 6. Initially, the protonation of alkyne leads to the formation of an alkenyl carbocation species **1** and **1′**. Further dearomatization of electrophilic addition intermediates **2** and **2′** gives the target products **2a′** and **3a′**, respectively.

## 3. Experimental Section

General Procedures for Substrate I Preparation [35].

Diphenylphosphine chloride (40 mmol) in CH_2_Cl_2_ (50 mL) was added dropwise to a stirred and cooled (0 °C) solution of acetylenic alcohol (40 mmol) in anhydrous ether (50 mL) and pyridine (3.88 mL, 48 mmol) under nitrogen. The stirring was maintained 1 h at 0 °C and at room temperature overnight. The solution was then quenched with cold water and extracted with CH_2_Cl_2_, and the organic layer was dried with Na_2_SO_4_. Concentration in vacuo gave the crude product that was subjected to flash chromatography on silica gel eluting with ethyl acetate and petroleum ether.

Note: The acetylenic alcohols were synthesized from terminal alkynes with corresponding ketones mediated by n-BuLi [36].

General Procedures for Substrate II Preparation [36].

To a 25 mL vial, allenylphosphine oxides (1 mmol), alkynes (2 mmol), potassium carbonate (K_2_CO_3_) (2 mmol), DMSO (10 mL), and Pd-NPS (1 mol%, 16 mg) were added. The reaction was then allowed to react at 100 °C for a certain time until the complete consumption of starting materials monitored via TLC. The reaction mixture was extracted with EtOAc (10 mL × 3). The combined organic extract was washed with brine and dried over anhydrous Na_2_SO_4_. The solvent was evaporated under reduced pressure, and the residue was purified using column chromatography on silica gel using petroleum ether/ethylacetate (1.5/1) as the eluent to afford the enynes. After this was finished, the compound was determined through NMR. Further, **1a**–**1n** are known compounds; please refer to [37].

General Procedures for Substituents Regulate the Cyclization of Conjugated Alkynes Constructing Cyclo-(*Z*)-[3]Dendralenes.

A 10 mL flask equipped with a magnetic stirrer was charged with conjugated alkynes (**1a**, 50 mg, 0.1 mmol), TFA (3.4 mg, 30 mol%), and 3 mL DCM. The reaction mixture was then heated to 50 °C for 3 h until the complete consumption of **1a** monitored via TLC. Subsequently, all of the volatiles were removed under vacuum, and the crude product was purified on flash chromatography (eluent: 1:1 (*v/v*) of ethyl acetate/petroleum ether) to afford product **2a** (45 mg, 90%) as a yellow solid.

Products of **2p′**. *(E)-(1-(1-(4-methylbenzylidene)-3-phenyl-1H-inden-2-yl)-2-(p-tolylthio)ethyl)diphenylphosphine oxide* (**2p′**). A yellow solid (92 mg, 80% yield), m.p.: 154.4–155.9 °C. ^1^H NMR (400 MHz, CDCl_3_) δ 8.07 (d, *J* = 8.5 Hz, 2H), 7.66–7.46 (m, 7H), 7.45–7.23 (m, 10H), 7.22–7.05 (m, 5H), 6.95 (d, *J* = 8.1 Hz, 2H), 6.88 (d, *J* = 8.1 Hz, 2H), 5.76 (d, *J* = 7.5 Hz, 1H), 3.97 (m, 1H), 3.89–3.71 (m, 1H), 3.44 (m, 1H), 2.53 (s, 3H), 2.32 (s, 3H). ^13^C NMR (101 MHz, CDCl_3_) δ 140.2 (d, *J* = 7.5 Hz), 137.8, 137.5, 137.1, 135.9, 133.2, 133.1, 132.6, 132.4, 132.1, 131.8, 131.7, 131.6, 131.4, 131.2, 130.9, 130.34 (d, *J* = 15.8 Hz), 130.3, 129.56 (d, *J* = 5.1 Hz), 129.6, 129.4, 129.0, 128.8, 128.7, 128.2, 128.0, 127.8, 127.7, 127.3, 127.1, 126.8, 126.1, 125.7, 125.6, 43.6, 43.0, 35.4, 21.4, 21.0. ^31^P NMR (162 MHz, CDCl_3_) δ 33.47 (s). HRMS (ESI): ([M + H]^+^) calcd for C_44_H_38_OPS^+^: 645.2381, found: 645.2379. IR (film) ν 3581, 2973, 2921, 1677, 1539, 1479, 1241, 1109, 1027, 939, 741, and 689 cm^−1^.

Products of **2a**. *(E)-(1-(1-benzylidene-3-phenyl-1H-inden-2-yl)vinyl)diphenylphosphine oxide* (**2a**). A yellow solid (84 mg, 83% yield), m.p.: 137.4–139.1 °C. ^1^H NMR (400 MHz, CDCl_3_) δ 7.50 (m, 4H), 7.45–7.32 (m, 9H), 7.29–7.22 (m, 7H), 7.18–7.09 (m, 2H), 7.07 (s, 1H), 6.97 (t, *J* = 7.3 Hz, 1H), 6.72 (m, 1H), 6.46 (m, 1H). ^13^C NMR (101 MHz, CDCl_3_) δ 143.7, 141.9, 141.1, 139.7, 137.4, 136.5, 135.4, 134.4, 133.9, 131.9 (d, *J* = 9.6 Hz), 131.5 (d, *J* = 2.7 Hz), 130.5, 129.4 (d, *J* = 13.1 Hz), 128.3, 128.2, 128.1, 128.0, 127.8, 127.7, 125.5, 123.1, 120.2. ^31^P NMR (162 MHz, CDCl_3_) δ 26.04 (s). HRMS (ESI): ([M + H]^+^) calcd for C_36_H_29_OP^+^: 507.1878, found: 507.1876. IR (film) ν 3677, 2985, 2907, 2221, 1592, 1574, 1496, 1438, 1172, 1077, 916, 739, and 695 cm^−1^.

Products of **2b**. *(E)-(1-(1-(4-fluorobenzylidene)-3-phenyl-1H-inden-2-yl)vinyl)diphenylphosphine oxide* (**2b**). A yellow solid (89 mg, 85% yield), m.p.: 107.4–109.1 °C. ^1^H NMR (400 MHz, CDCl_3_) δ 7.53–7.43 (m, 8H), 7.43–7.32 (m, 12H), 7.28–7.20 (m, 16H), 7.19–7.04 (m, 8H), 7.02–6.95 (m, 4H), 6.72 (d, *J* = 1.6 Hz, 1H), 6.67 (d, *J* = 1.6 Hz, 1H), 6.49 (d, *J* = 1.6 Hz, 1H), 6.40 (d, *J* = 1.6 Hz, 1H). ^13^C NMR (101 MHz, CDCl_3_) δ 161.4), 143.7, 142.0, 141.2, 140.6 139.7, 137.4, 135.3, 134.25 (d, *J* = 11.1 Hz), 133.7, 132.19 (d, *J* = 48.1 Hz), 132.0, 131.9, 131.6, 131.5, 131.2, 131.1, 130.4, 129.4, 128.3, 128.1, 128.0, 127.9, 127.8, 125.5, 122.9, 120.3, 115.5, 115.2, 100.0. ^31^P NMR (162 MHz, CDCl_3_) δ 26.15 (s). HRMS (ESI): ([M + H]^+^) calcd for C_36_H_26_FOP: 525.1784, found: 525.1783. IR (film) ν 3677, 2985, 2900, 2213, 1502, 1396, 1245, 1061, 924, 723, and 694 cm^−1^.

Products of **2c**. *(E)-(1-(1-(4-chlorobenzylidene)-3-phenyl-1H-inden-2-yl)vinyl)diphenylphosphine oxide* (**2c**). A yellow solid (82 mg, 77% yield), m.p.: 111.6–113.7 °C. ^1^H NMR (400 MHz, CDCl_3_) δ 7.54–7.31 (m, 26H), 7.22 (m, 16H), 7.13 (m, 4H), 6.98 (m, 4H), 6.73 (s, 1H), 6.69 (s, 1H), 6.49 (s, 1H), 6.40 (s, 1H). ^13^C NMR (101 MHz, CDCl_3_) δ 143.7, 141.6, 134.9, 134.1, 133.7 (d, *J* = 17.0 Hz), 131.9 (d, *J* = 9.6 Hz), 131.6, 130.7, 129.4, 128.5, 128.3, 128.2, 128.1, 128.0, 127.9, 125.6, 122.9, 120.4. ^31^P NMR (162 MHz, CDCl_3_) δ 26.47 (s). HRMS (ESI): ([M + H]^+^) calcd for C_36_H_27_ClOP^+^: 541.1488, found: 537.2322. IR (film), ν 3059, 2947, 2837, 2385, 1544, 1423, 1422, 1343, 1023, 881, 735, and 675 cm^−1^.

Products of **2d**. *(E)-4-((2-(1-(diphenylphosphoryl)vinyl)-3-phenyl-1H-inden-1-ylidene)methyl)benzonitrile* (**2d**). A yellow solid (98 mg, 87% yield), m.p.: 125.7–127.6 °C. ^1^H NMR (400 MHz, CDCl_3_) δ 7.68 (d, *J* = 8.2 Hz, 4H), 7.55–7.32 (m, 24H), 7.31–7.20 (m, 15H), 7.20–7.06 (m, 4H), 7.02–6.94 (m, 4H), 6.67 (d, *J* = 1.3 Hz, 1H), 6.62 (d, *J* = 1.3 Hz, 1H), 6.49 (d, *J* = 1.4 Hz, 1H), 6.39 (d, *J* = 1.4 Hz, 1H). ^13^C NMR (101 MHz, CDCl_3_) δ 143.9, 143.0, 141.5, 137.8, 134.0, 133.3, 132.3, 132.1, 131.9, 131.8, 131.7, 131.6, 131.3, 131.3, 130.3, 130.0, 129.3, 128.45 (d, *J* = 20.2 Hz), 128.13 (d, *J* = 12.0 Hz), 125.9, 122.9, 120.6, 118.8, 111.6, 100.0. ^31^P NMR (162 MHz, CDCl_3_) δ 26.27 (s). HRMS (ESI): ([M + H]^+^) calcd for C_37_H_27_NOP^+^: 532.1830, found: 532.1830. IR (film) ν 3675, 2970, 2906, 2231, 1537, 1338, 1244, 1044, 891, and 694 cm^−1^.

Products of **2e**. *(E)-(1-(1-(4-nitrobenzylidene)-3-phenyl-1H-inden-2-yl)vinyl)diphenylphosphine oxide* (**2e**). A yellow solid (65 mg, 56% yield), m.p.: 186.3–188.0 °C. ^1^H NMR (400 MHz, CDCl_3_) δ 7.67 (d, *J* = 7.5 Hz, 2H), 7.63–7.47 (m, 12H), 7.45–7.26 (m, 10H), 7.17 (s, 5H), 6.14 (d, *J* = 12.1 Hz, 1H), 6.05 (d, *J* = 12.1 Hz, 1H), 5.89 (d, *J* = 42.0 Hz, 1H), 5.46 (d, *J* = 19.4 Hz, 1H). ^13^C NMR (101 MHz, CDCl_3_) δ 145.2 (d, *J* = 7.5 Hz), 143.4 (d, *J* = 94.9 Hz), 142.4, 142.1, 140.9, 139.7, 136.0, 135.5 (d, *J* = 10.1 Hz), 133.7 (d, *J* = 7.2 Hz), 133.0, 132.7, 131.9, 131.8, 131.5 (d, *J* = 2.6 Hz), 130.9, 130.0, 129.8, 128.9, 128.3, 128.2, 127.9, 127.6, 127.3 (d, *J* = 8.1 Hz), 127.0, 126.9, 126.3. ^31^P NMR (162 MHz, CDCl_3_) δ 28.67 (s). HRMS (ESI): ([M + H]+) calcd for C_42_H_34_OP^+^: 585.2342, found: 585.2341. IR (film) ν 3062, 3015, 1599, 1487, 1435, 1195, 1111, 966, 860, and 690 cm^−1^.

Products of **2f***. Methyl-(E)-4-((2-(1-(diphenylphosphoryl)vinyl)-3-phenyl-1H-inden-1-ylidene)methyl)benzoate* (**2f**). A yellow solid (98 mg, 91% yield), m.p.: 185.5–187.4 °C. ^1^H NMR (400 MHz, CDCl_3_) δ 8.06 (d, *J* = 8.2 Hz, 4H), 7.46 (M, 13H), 7.35 (M, 9H), 7.23 (t, *J* = 7.9 Hz, 9H), 7.13 (M, 5H), 7.07–6.92 (m, 5H), 6.76 (s, 1H), 6.71 (s, 1H), 6.54 (d, *J* = 4.6 Hz, 1H), 6.45 (s, 1H), 3.97 (s, 3H). ^13^C NMR (101 MHz, CDCl_3_) δ 152.2, 152.1, 132.3, 132.2, 131.9, 131.8, 131.7, 130.3 (d, *J* = 7.0 Hz), 130.2, 128.4, 128.3, 128.2, 128.0, 127.9, 127.8, 127.6, 89.5, 21.2. ^31^P NMR (162 MHz, CDCl_3_) δ 27.80 (s). HRMS (ESI): ([M + H]^+^) calcd for C_37_H_32_O_2_P^+^:C_38_H_30_O_3_P:565.1933, found: 565.1931. IR (film) ν 3676, 2985, 2901, 1605, 1524, 1455, 1255, 1108, 1021, 945, 765, and 696 cm^−1^.

Products of **3g**. *Diphenyl(1-(1-phenyl-4-(m-tolyl)naphthalen-2-yl)vinyl)phosphine oxide* (**3g**). A yellow solid (70 mg, 67% yield), m.p.: 179.5–181.6 °C. ^1^H NMR (400 MHz, CDCl_3_) δ 7.97 (d, *J* = 8.1 Hz, 1H), 7.62–7.52 (m, 7H), 7.40 (m, 16H), 7.25–6.99 (m, 14H), 6.00 (m, 1H), 5.93 (d, *J* = 8.2 Hz, 1H), 2.42 (s, 3H). ^13^C NMR (101 MHz, CDCl_3_) δ 143.8, 139.0, 138.5, 137.2, 137.0, 134.8 133.6, 133.2, 133.1, 132.4, 132.3 (d, *J* = 2.7 Hz), 131.9, 131.8, 131.4, 131.1, 130.8, 130.0, 128.9, 128.5, 128.3, 127.8, 127.5, 127.3, 127.0, 125.9, (d, *J* = 5.1 Hz), 21.3. ^31^P NMR (162 MHz, CDCl_3_) δ 28.58 (s). HRMS (ESI): ([M + H]^+^) calcd for C_37_H_30_OP^+^: 521.2034, found: 521.2033. IR (film) ν 3645, 3408, 3235, 2985, 2917, 1634, 1594, 1435, 1245, 1093, 834, 758, and 695 cm^−1^.

Products of **3h**. *Diphenyl(1-(1-phenyl-4-(p-tolyl)naphthalen-2-yl)vinyl)phosphine oxide* (**3h**). A yellow solid (65 mg, 61% yield), m.p.: 167.3–168.8 °C. ^1^H NMR (400 MHz, CDCl_3_) δ 7.98 (d, *J* = 8.1 Hz, 1H), 7.67–7.47 (m, 7H), 7.46–7.32 (m, 9H), 7.30–7.21 (m, 4H), 7.17 (dd, *J* = 6.4, 3.0 Hz, 2H), 7.11 (s, 1H), 6.09–5.96 (m, 1H), 5.93 (s, 1H), 2.47 (s, 3H). ^13^C NMR (101 MHz, CDCl_3_) δ 144.7, 143.8, 139.0 (s), 138.7 (d, *J* = 47.4 Hz), 137.2, 137.0, 134.8, 133.6, 133.2 (d, *J* = 8.5 Hz), 132.3 (d, *J* = 9.6 Hz), 131.9 (d, *J* = 2.7 Hz), 131.4, 131.1, 130.8, 130.0, 128.9 128.4 (d, *J* = 12.0 Hz), 127.8, 127.4 (d, *J* = 16.2 Hz), 127.0, 125.9 (d, *J* = 5.6 Hz), 21.3. ^31^P NMR (162 MHz, CDCl_3_) δ 28.17 (s), HRMS (ESI): ([M + H]^+^) calcd for C_37_H_30_OP^+^: 521.2034, found: 521.2039. IR (film) ν 3645, 3051, 2968, 2900, 2228, 1603, 1503, 1432, 1177, 950, 865, and 694 cm^−1^.

Products of **3i**. *(1-(4-(4-methoxyphenyl)-1-phenylnaphthalen-2-yl)vinyl)Diphenylphosphine oxide* (**3i**). A yellow solid (91 mg, 80% yield), m.p.: 181.5–183.4 °C. ^1^H NMR (400 MHz, CDCl_3_) δ 7.98 (d, *J* = 8.3 Hz, 1H), 7.68–7.46 (m, 7H), 7.46–7.31 (m, 9H), 7.30–7.22 (m, 2H), 7.15 (dd, *J* = 6.4, 3.0 Hz, 2H), 7.09 (s, 1H), 6.99 (d, *J* = 8.6 Hz, 2H), 6.01 (d, *J* = 22.2 Hz, 1H), 5.93 (s, 1H), 3.90 (s, 3H). ^13^C NMR (101 MHz, CDCl_3_) δ 159.0, 144.4, 138.7, 138.5, 135.1, 133.6, 133.1, 132.5, 132.3, 132.2, 132.0 (d, *J* = 2.7 Hz), 131.5 (d, *J* = 10.3 Hz), 131.1, 130.5, 128.4 (d, *J* = 12.1 Hz), 127.8, 127.4 (d, *J* = 18.5 Hz), 127.0, 125.9, 113.7, 55.4. ^31^P NMR (162 MHz, CDCl_3_) δ 28.86 (s). HRMS (ESI): ([M + H]^+^) calcd for C_37_H_30_O_2_P: 537.1983, found: 537.1986. IR (film) ν 3365, 2765, 2656, 2344, 1581, 1492, 1488, 1385, 1055, 883, 745, and 635 cm^−1^.

Products of **3j**. (*1-(4-(4-(tert-butyl)phenyl)-1-phenylnaphthalen-2-yl)vinyl)diphenylphosphine oxide* (**3j**). A yellow solid (62 mg, 56% yield), m.p.: 195.2–197.7 °C. ^1^H NMR (400 MHz, CDCl_3_) δ 8.01 (d, *J* = 8.3 Hz, 1H), 7.55 (m, 6H), 7.52–7.32 (m, 13H), 7.33–7.24 (m, 4H), 7.13 (m, 4H), 5.95 (dd, *J* = 29.9, 16.8 Hz, 2H), 1.43 (s, 9H). ^13^C NMR (101 MHz, CDCl_3_) δ 150.2, 144.8, 138.9, 138.5, 137.1, 134.8, 133.6, 132.27 (d, *J* = 9.7 Hz), 131.8, 131.4, 131.0, 129.8, 128.4 (d, *J* = 12.0 Hz), 127.7, 127.4, 127.0, 126.0 (d, *J* = 18.2 Hz), 125.1, 124.7, 34.6, 31.6,31.5. ^31^P NMR (162 MHz, CDCl_3_) δ 28.82 (s). HRMS (ESI): ([M + H]^+^) calcd for C_40_H_36_OP^+^: 563.2504, found: 563.2501. IR (film) ν 3012, 2945, 2832, 2375, 1545, 1423, 1425, 1315, 1054, 945, 738, and 645 cm^−1^.

Products of dendralenes. *(1-(4-([1,1’-biphenyl]-4-yl)-1-phenylnaphthalen-2-yl)vinyl)diphenylphosphine oxide* (**3k**). A yellow solid (87 mg, 83% yield), m.p.: 112.1–113.3 °C. ^1^H NMR (400 MHz, CDCl_3_) δ 8.04 (d, *J* = 8.3 Hz, 1H), 7.70 (m, 4H), 7.57 (m, 9H), 7.48–7.34 (m, 12H), 7.22–7.11 (m, 3H), 6.00 (d, *J* = 25.8 Hz, 1H), 5.92 (d, *J* = 4.3 Hz, 1H). ^13^C NMR (101 MHz, CDCl_3_) δ 140.8, 140.1, 139.2, 138.9,138.8, 138.5 (d, *J* = 11.0 Hz), 134.9 (d, *J* = 9.2 Hz), 133.7, 133.3, 133.2, 132.4,132.3, 131.9,131.8, 131.4, 131.0, 130.53 (s), 130.8, 128.9, 128.5, 128.4, 127.8, 127.6,127.5, 127.2, 127.1, 126.9, 126.1, 126.0, 125.8. ^31^P NMR (162 MHz, CDCl_3_) δ 28.27 (s). HRMS (ESI): ([M + H]^+^) calcd for C_42_H_32_OP^+^: 583.2191, found: 583.2195. IR (film) ν 3668, 2983, 2902, 2214, 1605, 1401, 1254, 1065, 894, and 693 cm^−1^.

Products of **3i.** *Diphenyl(1-(1-phenyl-4-(pyridin-3-yl)naphthalen-2-yl)vinyl)phosphine oxide* (**3i**). A yellow solid (74 mg, 70% yield), m.p.: 146.2–148.5 °C. ^1^H NMR (400 MHz, CDCl_3_) δ 8.40 (s, 1H), 8.00–7.60 (m, 5H), 7.59–6.82 (m, 18H), 5.97 (dd, *J* = 54.8, 28.6 Hz, 2H). ^13^C NMR (101 MHz, CDCl_3_) δ 150.8, 141.2, 140.2, 138.7, 132.1 (d, *J* = 10.0 Hz), 131.0, 130.2 (d, *J* = 18.0 Hz), 128.5, 128.4, 128.0, 127.8, 127.7, 126.7, 123.0, 100.0, 96.4. ^31^P NMR (162 MHz, CDCl_3_) δ 28.62 (s). HRMS (ESI): ([M + H]^+^) calcd for C_35_H_27_NOP^+^: 508.1830, found: 508.1835. IR (film) ν 3653, 3382, 2933, 2223, 1548, 1435, 1173, 957, 795, and 695 cm^−1^.

Products of **3m.** *Diphenyl(1-(1-phenyl-4-(thiophen-3-yl)naphthalen-2-yl)vinyl)phosphine oxide* (**3m**). A yellow solid (76 mg, 75% yield), m.p.: 173.2–174.7 °C. ^1^H NMR (400 MHz, CDCl_3_) δ 8.08 (d, *J* = 8.4 Hz, 1H), 7.60–7.49 (m, 5H), 7.48–7.33 (m, 9H), 7.20 (M, 1H), 7.14 (M, 3H), 5.97 (dd, *J* = 30.5, 20.9 Hz, 2H). ^13^C NMR (101 MHz, CDCl_3_) δ 140.5, 139.0, 138.2, 135.6, 133.7 (d, *J* = 29.3 Hz), 132.4, 132.3, 132.1, 131.3, 131.2, 129.6, 128.6, 128.5, 128.4, 127.8, 127.7, 127.6, 127.4, 127.1, 127.0, 126.2, 126.1, 126.0, 125.7, 125.5, 125.3, 123.7. ^31^P NMR (162 MHz, CDCl_3_) δ 30.37 (s). HRMS (ESI): ([M + H]^+^) calcd for C_34_H_26_OPS^+^: 513.1442, found: 513.1447. IR (film) ν 3675, 3050, 2223, 1678, 1585, 1437, 1314, 126.6, 117.03, 112.7, 903, and 853 cm^−1^.

## 4. Conclusions

In summary, we revealed that an unprecedented substituent electron effect regulates the cyclization reactions of conjugated alkynes via Lewis acid catalysis, providing a novel and efficient method for the construction of cyclic-(*E*)-[3]dendralenes from alkynes with remarkable functional group tolerance and good yields. This strategy features easily accessible substrates, operational simplicity, and expands the pool of cyclic-[3]dendralenes in organic synthesis.

## Figures and Tables

**Table 1:**
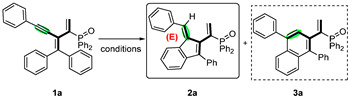
Optimization of the reaction conditions ^a^.

Entry	Catalyst	Solvent	Yield (2a/3a, %) ^b^
1	AgSbF_6_	DCM	90/trace
2	AgSbF_6_	DMF	0/0
3	AgSbF_6_	1,4-dioxane	78/<5
4	AgSbF_6_	toluene	63/<5
5	AgSbF_6_	CH_3_CN	0/0
6	AgCl	DCM	trace/0
7	AgOAc	DCM	trace/0
8	H_3_PO_4_	DCM	40/0
9	TsOH	DCM	61/0
10	TFA	DCM	90/0
11	Pivalic acid	DCM	0/0
12 ^c^	TFA	DCM	trace/0
13 ^d^	TFA	TFA	87/<5

^a^ Reaction conditions: Phosphinyl-conjugated enynes (1a, 0.1 mmol), TFA (30 mol%), solvent (3 mL), 40 °C, 3 h. ^b^ Isolated yield. ^c^ 10 mol% catalyst. ^d^ TFA (3.0 mL) as solvent.

## Data Availability

The data presented in this study can be found in the article or in the associated Supplementary Materials.

## Supplementary Materials

The following supporting information can be downloaded at: https://www.mdpi.com/article/10.3390/molecules28114382/s1. Experimental procedures and spectral data for all new compounds (PDF). This material is available free of charge via the Internet at http://pubs.acs.org. Reference [39] is cited in the Supplementary Materials.

## Sample Availability

Samples of the compounds **2b**–**2f** and **3g**–**n** are available from the authors.

## References

[1] Sherburn M.S. (2015). Preparation and Synthetic Value of π-Bond-Rich Branched Hydrocarbons. Acc. Chem. Res..

[2] Mackay E.G., Sherburn M.S. (2013). Demystifying the dendralenes. Pure Appl. Chem..

[3] Hopf H. (2009). Forgotten hydrocarbons prepared. Nature.

[4] Payne A.D., Bojase G., Paddon-Row M.N., Sherburn M.S. (2009). Practical Synthesis of the Dendralene Family Reveals Alternation in Behavior. Angew. Chem. Int. Ed..

[5] Takenaka K., Amamoto S., Kishi H., Takeshita H., Miya M., Shiomi T. (2013). Anionic Polymerization of 2-Phenyl[3]dendralene and 2-(4-Methoxyphenyl)[3]dendralene. Macromolecules.

[6] Takamura Y., Takenaka K., Toda T., Takeshita H., Miya M., Shiomi T. (2018). Anionic Polymerization of 2-Hexyl [3]dendralene. Macromol. Chem. Phys..

[7] Ravinder P., Subramanian V. (2012). Theor. Density functional theory studies on the Diels–Alder reaction of [3]dendralene with C_60_: An attractive approach for functionalization of fullerene. Chem. Acc..

[8] Paddon-Row M.N., Sherburn M.S. (2012). On the origin of the alternating Diels–Alder reactivity in [n]dendralenes. Chem. Commun..

[9] Saglam M.F., Fallon T., Paddon-Row M.N., Sherburn M.S. (2016). Discovery and Computational Rationalization of Diminishing Alternation in [n]Dendralenes. J. Am. Chem. Soc..

[10] Hosoya H. (2019). Mathematical Features of the Genealogy of Acyclic Conjugated Polyenes. Bull. Chem. Soc. Jpn..

[11] Breiten B., Wu Y.-L., Jarowski P.D., Gisselbrecht J.-P., Boudon C., Griesser M., Onitsch C., Gescheidt G., Schweizer W.B., Langer N. (2011). Donor-substituted octacyano [4]dendralenes: A new class of cyano-rich non-planar organic acceptors. Chem. Sci..

[12] Shoji T., Miura K., Araki T., Maruyama A., Ohta A., Sekiguchi R., Ito S., Okujima T. (2018). Synthesis of 2-Methyl-1-azulenyl Tetracyanobutadienes and Dicyanoquinodimethanes: Substituent Effect of 2-Methyl Moiety on the Azulene Ring toward the Optical and Electrochemical Properties. J. Org. Chem..

[13] Michinobu T., Diederich F. (2018). The [2 + 2] Cycloaddition-Retroelectrocyclization (CA-RE) Click Reaction: Facile Access to Molecular and Polymeric Push-Pull Chromophores. Angew. Chem. Int. Ed..

[14] Pronin S.V., Shenvi R.A. (2012). Synthesis of a Potent Antimalarial Amphilectene. J. Am. Chem. Soc..

[15] Volla C.M.R., Bäckvall J.-E. (2013). Palladium-Catalyzed Aerobic Domino Oxidative Carbocyclization-Alkynylation of Allenynes. Angew. Chem. Int. Ed..

[16] Fallon T., Willis A.C., Paddon-Row M.N., Sherburn M.S. (2014). Furanodendralenes. J. Org. Chem..

[17] Bartholomeyzik T., Mazuela J., Pendrill R., Deng Y., Bäckvall J.-E. (2014). Palladium-Catalyzed Oxidative Arylating Carbocyclization of Allenynes: Control of Selectivity and Role of H_2_O. Angew. Chem. Int. Ed..

[18] Thies N., Haak E. (2015). Ruthenium-Catalyzed Synthesis of 2,3-Cyclo[3]dendralenes and Complex Polycycles from Propargyl Alcohols. Angew. Chem. Int. Ed..

[19] Tan S.M., Willis A.C., Paddon-Row M.N., Sherburn M.S. (2016). Multicomponent Diene-Transmissive Diels–Alder Sequences Featuring Aminodendralenes. Angew. Chem. Int. Ed..

[20] Sakashita K., Shibata Y., Tanaka K. (2016). Rhodium-Catalyzed Cross-Cyclotrimerization and Dimerization of Allenes with Alkynes. Angew. Chem. Int. Ed..

[21] Lippincott D.J., Linstadt R.T.H., Maser M.R., Lipshutz B.H. (2017). Synthesis of Functionalized [3], [4], [5] and [6]Dendralenes through Palladium-Catalyzed Cross-Couplings of Substituted Allenoates. Angew. Chem. Int. Ed..

[22] Qiu Y., Posevins D., Bäckvall J.-E. (2017). Selective Palladium-Catalyzed Allenic C−H Bond Oxidation for the Synthesis of [3]Dendralenes. Angew. Chem. Int. Ed..

[23] Rivera-Chao E., Fañanás-Mastral M. (2018). Synthesis of Stereodefined Borylated Dendralenes through Copper-Catalyzed Allylboration of Alkynes. Angew. Chem. Int. Ed..

[24] Li L., Luo P., Deng Y., Shao Z. (2019). Regioselectivity Switch in Palladium-Catalyzed Allenylic Cycloadditions of Allenic Esters: [4 + 1] or [4 + 3] Cycloaddition/Cross-Coupling. Angew. Chem. Int. Ed..

[25] Li H., Gontla R., Flegel J., Merten C., Ziegler S., Antonchick A.P., Waldmann H. (2019). Enantioselective Formal C(sp^3^)−H Bond Activation in the Synthesis of Bioactive Spiropyrazolone Derivatives. Angew. Chem. Int. Ed..

[26] Li S.C., Hou B., Wang J.B. (2021). Palladium-Catalyzed Oxidative Coupling of the Allenic C–H Bond with α-Diazo Esters: Synthesis of [3]Dendralenes. J. Org. Chem..

[27] Hopf H., Sherburn M.S. (2012). Dendralenes Branch Out: Cross-Conjugated Oligoenes Allow the Rapid Generation of Molecular Complexity. Angew. Chem. Int. Ed..

[28] Zhu J., Yang W.C., Zhang C.Y., Wu L. (2021). Recent Progress in the Synthesis of Dendralenes: A Decade Update. Chin. J. Org. Chem..

[29] Mao M., Zhang L., Chen Y.-Z., Zhu J., Wu L. (2017). Palladium-Catalyzed Coupling of Allenylphosphine Oxides with N-Tosylhydrazones toward Phosphinyl [3]Dendralenes. ACS Catal..

[30] Bartholomeyzik T., Pendrill R., Lihammar R. (2018). Kinetics and Mechanism of the Palladium-Catalyzed Oxidative Arylating Carbocyclization of Allenynes. J. Am. Chem. Soc..

[31] Wei K., Luo K., Liu F., Wu L., Wu L.-Z. (2019). Visible-Light-Driven Selective Alkenyl C–P Bond Cleavage of Allenylphosphine Oxides. Org. Lett..

[32] Zhu J., Mao M., Ji H.-J., Xu J.-Y., Wu L. (2017). Palladium-Catalyzed Cleavage of α-Allenylic Aryl Ether toward Pyrazolemethylene-Substituted Phosphinyl Allenes and Their Transformations via Alkenyl C–P(O) Cleavage. Org. Lett..

[33] Zhang L., Zhu J., Ma J., Wu L., Zhang W.-H. (2017). Visible-Light-Driven α-Allenylic C–O Bond Cleavage and Alkenyl C–S Formation: Metal-Free and Oxidant-Free Thiolation of Allenyl Phosphine Oxides. Org. Lett..

[34] Xia Y.T., Wu J.J., Zhang C.Y., Mao M., Ji Y.G., Wu L., Zhang W.H. (2019). Cascade Alkynylation and Highly Selective Hydrogenation Catalyzed by Binaphthyl-Palladium Nanoparticles Accessing Phosphinyl (Z)-[3]Dendralenes. Org. Lett..

[35] Chen Y.-Z., Zhang L., Lu A.-M., Yang F., Wu L. (2015). α-Allenyl Ethers as Starting Materials for Palladium Catalyzed Suzuki–Miyaura Couplings of Allenylphosphine Oxides with Arylboronic Acids. J. Org. Chem..

[36] Liu P., Deng C.-L., Lei X., Lin G. (2011). Tandem Amination/Cycloisomerization of Aryl Propargylic Alcohols with 2-Aminopyridines as an Expedient Route to Imidazo [1,2-a]pyridines. Eur. J. Org. Chem..

[37] Xia Y.T., Xie X.Y., Cui S.H., Ji Y.G., Wu L. (2019). Secondary phosphine oxides stabilized Au/Pd nanoalloys: Metal components-controlled regioselective hydrogenation toward phosphinyl (Z)-[3]dendralenes. Chem. Commun..

[38] Franco M., Azzena U., Melloni G. (1993). A novel synthesis of methylene-1H-indenes (benzofulvenes) by cyclisation of phenyl-substituted but-1-en-3-ynes. J. Chem. Soc. Perkin Trans..

[39] Luo X.-L., Chen X.-W., Chen L., Zhang K., Chen Y.-B. (2019). Xanthate-mediated synthesis of (*E*)-alkenes by semi-hydrogenation of alkynes using water as the hydrogen donor. Chem. Commun..

