# Supplementary material for: Substituents Regulate the Cyclization of Conjugated Alkynes to Accurately Construct Cyclo-(E)-[3]dendralenes
Source: Molecules. 2023 May 27;28(11):4382. doi: 10.3390/molecules28114382 (PMC10254459; doi:10.3390/molecules28114382)
Supplement: Supplementary file 1 [file molecules-28-04382-s001.zip › molecules-2413660-supplementary.pdf]

Supporting Information

Substituents Regulate the Cyclization of Conjugated Alkynes to Accurately Construct cyclo-(*E*)-[3]Dendralenes

Yun-Tao Xia,[†] Ya-Yin Li,[†] Tong-Tong Bi,[†] Wei Lian,[†] Xia Wang,[†] Meng Yan,[†]Tao Guo,[†] and Lei Wu,[‡]

[†]School of Chemistry & Chemical Engineering, Henan University of Technology, Academician Workstation for Natural Medicinal Chemistry of Henan Province, Zhengzhou 450001, P. R. China

[‡]Jiangsu Key Laboratory of Pesticide Science and Department of Chemistry, College of Sciences, Nanjing Agricultural University, Nanjing 210095, China

Table of Contents for Supporting Information

1. **General Information** 2

2. **General Procedures for Substrates Preparation** 2

3. **General Procedures for Substituents Regulate the Cyclization of Conjugated Alkynes (1p) Constructing 2p'** 3

4. **General Procedures for Substituents Regulate the Cyclization of Conjugated Alkynes constructing cyclo-(*E*)-[3]Dendralenes** .... **Error! Bookmark not defined.**

5. **X-Ray Crystallography Data of 2a** 4

6. **Characterizations of Products** 11

7. **^{1}H-NMR, ^{13}C-NMR, ^{31}P-NMR Spectra** 188

1. General Information

Solvents and reagents were reagent grade and used without purification unless otherwise noted. Column chromatography was performed using silica gel (200-300 mesh). All ^{1}H-NMR (400 MHz) spectra were recorded on a Bruker-DMX 400 using $CDCl_3$ solution in the presence of tetramethylsilane (TMS) as an internal standard and are reported in ppm (δ). Coupling constants are reported in Hertz (Hz). Spectral splitting patterns are designated as s, singlet; d, doublet; t, triplet; q, quartet; p, pentet; m, multiplet; and br, broad. High resolution mass spectroscopic data of the products were collected on a Waters Micromass GCT instrument using EI (70 eV) or an Agilent Technologies 6540 UHD Accurate-Mass Q-TOF LC/MS using ESI.

2. General Procedures for Substrates Preparation

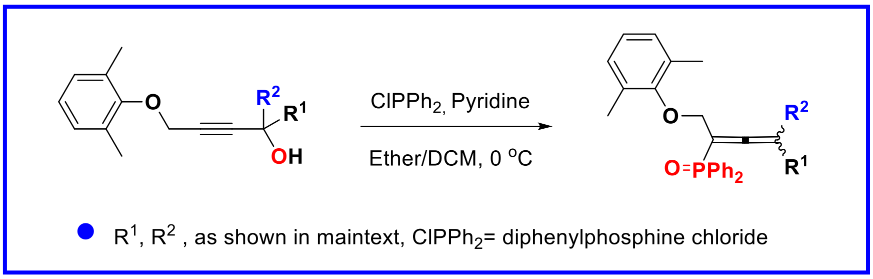

To a stirred and cooled (0 °C) solution of the acetylenic alcohol (40 mmol) in anhydrous ether (50 mL) and pyridine (3.88 mL, 48 mmol) under nitrogen was added dropwise diphenylphosphine chloride (40 mmol) in CH_2Cl_2 (50 mL). The stirring was maintained 1 h at 0 °C and at room temperature overnight. The solution was then quenched with cold water and extracted with CH_2Cl_2, and the organic layer was dried with Na_2SO_4. Concentration in vacuo gave the crude product that was subjected to flash chromatography on silica gel eluting with ethyl acetate and petroleum ether. [Ref.: Chen, Y.-Z.; Zhang, L.; Lu, A.-M.; Yang, F.; Wu, L. *J. Org. Chem.* **2015**, *80*, 673-680.]

Note: the acetylenic alcohols were synthesized from terminal alkynes with corresponding ketones mediated by n-BuLi. (Liu, P.; Deng, C.-L.; Lei, X.; Lin, G. Eur. *J. Org. Chem.* **2011**, 7308-7316.)

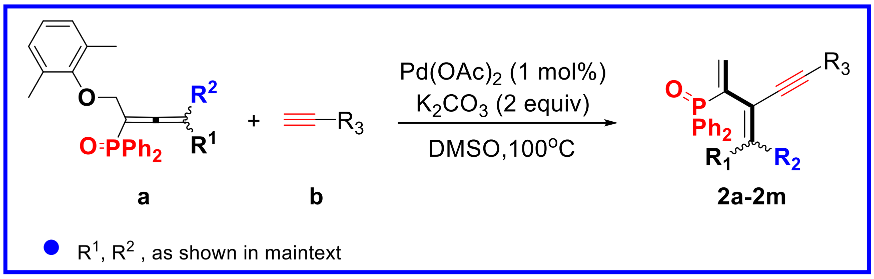

To a 25 mL vial was added allenylphosphine oxides (1 mmol), alkynes (2 mmol), potassium carbonate (K_2CO_3) (2 mmol), DMSO (10 mL) and $Pd(OAc)_2$ (1 mol%) respectively. The reaction was then allowed to react at 100 °C for a certain time until the complete consuming of

starting materials monitored by TLC. The reaction mixture was extracted with EtOAc (10 mL×3). The combined organic extract was washed with brine and dried over anhydrous Na_2SO_4. The solvent was evaporated under reduced pressure and the residue was purified by column chromatography on silica gel using petroleum ether/ethylacetate (1.5/1) as the eluent to afford the enynes. After being finished, the compound was determined by **NMR**.

Note: **1a-1n** is a known compound, please refer to the literature [4].

3. General Procedures for Substituents Regulate the Cyclization of Conjugated Alkynes Constructing Cyclo-(*E*)-[3]Dendralenes

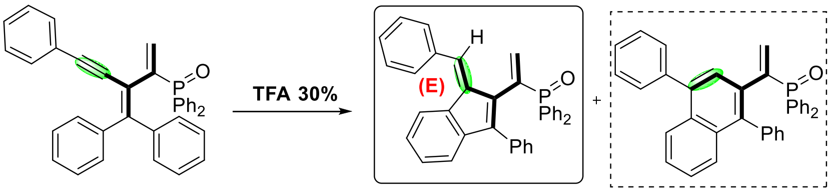

1a **2a** **3a**

TFA= Trifluoroacetic acid

A 10 mL flask equipped with a magnetic stirrer was charged with conjugated alkynes (**1a**, 50 mg, 0.1 mmol), TFA (3.4 mg, 30 mol%) and 3 mL DCM. The reaction mixture was then heated to 50 °C for 3 hour until the complete consuming of **1a** monitored by TLC. Subsequently, all of the volatiles were removed under vacuum, the crude product was purified on flash chromatography (eluent: 1:1 (v/v) of ethyl acetate/petroleum ether) to afford product **2a** (45 mg, 90%) as a yellow solid.

4. Control Experiments

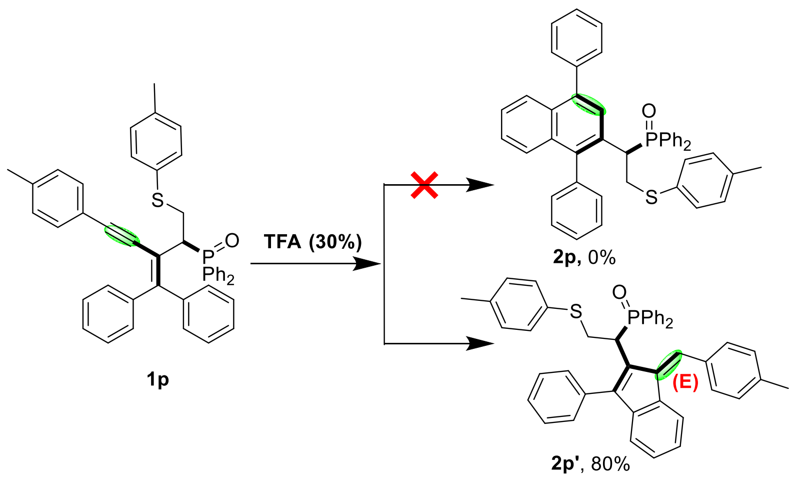

To a 25 mL vial was added Phosphinyl conjugated enynes (0.1 mmol, 64 mg), catalyst TFA (Trifluoroacetic acid, 30 mol%,3.4 mg) in 3 mL solvent (Dichloromethane) . The reaction was then allowed to react at 40 ^{o}C for a certain time until the complete consuming of starting materials monitored by TLC. The solvent was evaporated under reduced pressure and the residue was purified by column chromatography on silica gel using petroleum ether/ethylacetate (1/1) as the eluent to afford product **2p’** (5 mg, 80%) as A yellow solid, and the compound was determined by NMR and MS.

Note: 2p is not detected and the synthesis method of the five-membered episulfide compound was reported for the first time, and the product **2p’** was a new compound. [Ref.: Luo, X.-L; Chen, X,-W; Chen, L; Zhang, K and Chen, Y,-B; *Chem. Commun.*, **2019**, *55*, 2170.]

5. X-Ray Crystallography Data of 2a

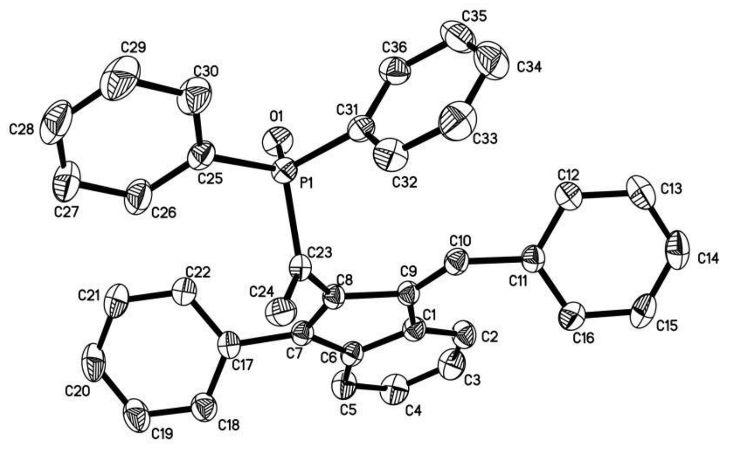

Table S1. Crystal data and structure refinement for 2a.

Identification code	2a
Empirical formula	$C_{36} H_{27}$ O P
Formula weight	506.54
Temperature	296(2) K
Wavelength	0.71073
Crystal system	Monoclinic
Space group	P21/c
Unit cell dimensions	a = 18.285(2)
	b = 9.1800(9)
	c = 15.9471(16)
Volume	2676.7(5) ?3
Z	4
Density (calculated)	1.257 Mg/m3
Absorption coefficient	0.131 mm-1
F(000)	1064
Crystal size	0.220 x 0.200 x 0.100 mm3
Theta range for data collection	1.114 to 27.530
Index ranges	-23<=h<=23, -11<=k<=10, -20<=l<=20
Reflections collected	26057
Independent reflections	6093 [R(int) = 0.0698]
Completeness to theta = 25.242?	99.6 %
Absorption correction	Semi-empirical from equivalents
Max. and min. transmission	0.7456 and 0.5546
Refinement method	Full-matrix least-squares on F2
Data / restraints / parameters	6093 / 0 / 343
Goodness-of-fit on F2	1.028
Final R indices [I>2sigma(I)]	R1 = 0.0595, wR2 = 0.1428
R indices (all data)	R1 = 0.1051, wR2 = 0.1630
Extinction coefficient	n/a
Largest diff. peak and hole	0.373 and -0.361 e.?-3

Table S2. Bond lengths for 2a.

C(1)-C(2)	1.388(3)
C(1)-C(6)	1.405(3)

C(1)-C(9)	1.483(3)
C(2)-C(3)	1.378(4)
C(2)-H(2)	0.9300
C(3)-C(4)	1.378(4)
C(3)-H(3)	0.9300
C(4)-C(5)	1.385(4)
C(4)-H(4)	0.9300
C(5)-C(6)	1.376(3)
C(5)-H(5)	0.9300
C(6)-C(7)	1.478(3)
C(7)-C(8)	1.352(3)
C(7)-C(17)	1.484(3)
C(8)-C(9)	1.481(3)
C(8)-C(23)	1.490(3)
C(9)-C(10)	1.335(3)
C(10)-C(11)	1.471(3)
C(10)-H(10)	0.9300
C(11)-C(12)	1.380(3)
C(11)-C(16)	1.382(3)
C(12)-C(13)	1.381(4)
C(12)-H(12)	0.9300
C(13)-C(14)	1.354(4)
C(13)-H(13)	0.9300
C(14)-C(15)	1.349(4)
C(14)-H(14)	0.9300
C(15)-C(16)	1.383(4)
C(15)-H(15)	0.9300
C(16)-H(16)	0.9300
C(17)-C(22)	1.387(3)
C(17)-C(18)	1.388(3)
C(18)-C(19)	1.385(4)
C(18)-H(18)	0.9300
C(19)-C(20)	1.372(4)
C(19)-H(19)	0.9300
C(20)-C(21)	1.361(4)
C(20)-H(20)	0.9300
C(21)-C(22)	1.381(3)
C(21)-H(21)	0.9300

C(22)-H(22)	0.9300
C(23)-C(24)	1.319(3)
C(23)-P(1)	1.823(2)
C(24)-H(24A)	0.9300
C(24)-H(24B)	0.9300
C(25)-C(30)	1.383(4)
C(25)-C(26)	1.386(4)
C(25)-P(1)	1.808(2)
C(26)-C(27)	1.384(4)
C(26)-H(26)	0.9300
C(27)-C(28)	1.358(5)
C(27)-H(27)	0.9300
C(28)-C(29)	1.351(5)
C(28)-H(28)	0.9300
C(29)-C(30)	1.391(4)
C(29)-H(29)	0.9300
C(30)-H(30)	0.9300
C(31)-C(36)	1.381(3)
C(31)-C(32)	1.392(3)
C(31)-P(1)	1.811(2)
C(32)-C(33)	1.375(4)
C(32)-H(32)	0.9300
C(33)-C(34)	1.385(5)
C(33)-H(33)	0.9300
C(34)-C(35)	1.367(4)
C(34)-H(34)	0.9300
C(35)-C(36)	1.382(4)
C(35)-H(35)	0.9300
C(36)-H(36)	0.9300
O(1)-P(1)	1.4854(16)

C(2)-C(1)-C(6)	119.1(2)
C(2)-C(1)-C(9)	132.9(2)
C(6)-C(1)-C(9)	107.74(18)
C(3)-C(2)-C(1)	119.6(2)
C(3)-C(2)-H(2)	120.2
C(1)-C(2)-H(2)	120.2
C(2)-C(3)-C(4)	120.8(2)

C(2)-C(3)-H(3)	119.6
C(4)-C(3)-H(3)	119.6
C(3)-C(4)-C(5)	120.5(2)
C(3)-C(4)-H(4)	119.7
C(5)-C(4)-H(4)	119.7
C(6)-C(5)-C(4)	119.1(2)
C(6)-C(5)-H(5)	120.5
C(4)-C(5)-H(5)	120.5
C(5)-C(6)-C(1)	120.8(2)
C(5)-C(6)-C(7)	131.0(2)
C(1)-C(6)-C(7)	108.01(19)
C(8)-C(7)-C(6)	109.18(19)
C(8)-C(7)-C(17)	127.7(2)
C(6)-C(7)-C(17)	122.83(19)
C(7)-C(8)-C(9)	109.54(18)
C(7)-C(8)-C(23)	126.25(19)
C(9)-C(8)-C(23)	123.94(18)
C(10)-C(9)-C(8)	123.1(2)
C(10)-C(9)-C(1)	131.4(2)
C(8)-C(9)-C(1)	105.44(17)
C(9)-C(10)-C(11)	130.4(2)
C(9)-C(10)-H(10)	114.8
C(11)-C(10)-H(10)	114.8
C(12)-C(11)-C(16)	117.7(2)
C(12)-C(11)-C(10)	119.2(2)
C(16)-C(11)-C(10)	123.0(2)
C(11)-C(12)-C(13)	120.4(3)
C(11)-C(12)-H(12)	119.8
C(13)-C(12)-H(12)	119.8
C(14)-C(13)-C(12)	121.0(3)
C(14)-C(13)-H(13)	119.5
C(12)-C(13)-H(13)	119.5
C(15)-C(14)-C(13)	119.4(3)
C(15)-C(14)-H(14)	120.3
C(13)-C(14)-H(14)	120.3
C(14)-C(15)-C(16)	120.7(3)
C(14)-C(15)-H(15)	119.6
C(16)-C(15)-H(15)	119.6

C(11)-C(16)-C(15)	120.7(2)
C(11)-C(16)-H(16)	119.7
C(15)-C(16)-H(16)	119.7
C(22)-C(17)-C(18)	118.1(2)
C(22)-C(17)-C(7)	119.8(2)
C(18)-C(17)-C(7)	122.1(2)
C(19)-C(18)-C(17)	120.5(3)
C(19)-C(18)-H(18)	119.7
C(17)-C(18)-H(18)	119.7
C(20)-C(19)-C(18)	120.1(3)
C(20)-C(19)-H(19)	120.0
C(18)-C(19)-H(19)	120.0
C(21)-C(20)-C(19)	120.2(3)
C(21)-C(20)-H(20)	119.9
C(19)-C(20)-H(20)	119.9
C(20)-C(21)-C(22)	120.2(3)
C(20)-C(21)-H(21)	119.9
C(22)-C(21)-H(21)	119.9
C(21)-C(22)-C(17)	120.9(2)
C(21)-C(22)-H(22)	119.6
C(17)-C(22)-H(22)	119.6
C(24)-C(23)-C(8)	120.4(2)
C(24)-C(23)-P(1)	120.17(17)
C(8)-C(23)-P(1)	119.41(15)
C(23)-C(24)-H(24A)	120.0
C(23)-C(24)-H(24B)	120.0
H(24A)-C(24)-H(24B)	120.0
C(30)-C(25)-C(26)	118.2(2)
C(30)-C(25)-P(1)	121.9(2)
C(26)-C(25)-P(1)	119.55(19)
C(27)-C(26)-C(25)	120.8(3)
C(27)-C(26)-H(26)	119.6
C(25)-C(26)-H(26)	119.6
C(28)-C(27)-C(26)	120.0(3)
C(28)-C(27)-H(27)	120.0
C(26)-C(27)-H(27)	120.0
C(29)-C(28)-C(27)	120.3(3)
C(29)-C(28)-H(28)	119.8

C(27)-C(28)-H(28)	119.8
C(28)-C(29)-C(30)	120.8(3)
C(28)-C(29)-H(29)	119.6
C(30)-C(29)-H(29)	119.6
C(25)-C(30)-C(29)	119.9(3)
C(25)-C(30)-H(30)	120.1
C(29)-C(30)-H(30)	120.1
C(36)-C(31)-C(32)	119.1(2)
C(36)-C(31)-P(1)	117.94(18)
C(32)-C(31)-P(1)	122.94(19)
C(33)-C(32)-C(31)	120.5(3)
C(33)-C(32)-H(32)	119.8
C(31)-C(32)-H(32)	119.8
C(32)-C(33)-C(34)	119.4(3)
C(32)-C(33)-H(33)	120.3
C(34)-C(33)-H(33)	120.3
C(35)-C(34)-C(33)	120.6(3)
C(35)-C(34)-H(34)	119.7
C(33)-C(34)-H(34)	119.7
C(34)-C(35)-C(36)	119.9(3)
C(34)-C(35)-H(35)	120.0
C(36)-C(35)-H(35)	120.0
C(31)-C(36)-C(35)	120.4(3)
C(31)-C(36)-H(36)	119.8
C(35)-C(36)-H(36)	119.8
O(1)-P(1)-C(25)	112.87(10)
O(1)-P(1)-C(31)	111.68(10)
C(25)-P(1)-C(31)	106.37(11)
O(1)-P(1)-C(23)	112.54(10)
C(25)-P(1)-C(23)	106.18(10)
C(31)-P(1)-C(23)	106.75(10)

Table S3. Hydrogen bonds [Å and °] for 2a.

D-H...A	d(D-H)	d(H...A)	d(D...A)	<(DHA)
C(36)-H(27)OP(1)	0.98	2.11	2.989(4)	147.6

Symmetry transformations used to generate equivalent atoms:

6. Characterizations of Products

6.1 products of 2p'

(*E*)-(1-(1-(4-methylbenzylidene)-3-phenyl-1H-inden-2-yl)-2-(p-tolylthio)ethyl)diphenylphosphine oxide (2p')

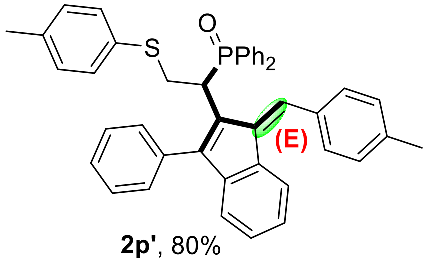

2p', 80%

A yellow solid, (92 mg, 80% yield), *m.p.*: 154.4-155.9 °C. **^{1}H NMR** (400 MHz, $CDCl_3$) δ 8.07 (d, J = 8.5 Hz, 2H), 7.66-7.46 (m, 7H), 7.45-7.23 (m, 10H), 7.22-7.05 (m, 5H), 6.95 (d, J = 8.1 Hz, 2H), 6.88 (d, J = 8.1 Hz, 2H), 5.76 (d, J = 7.5 Hz, 1H), 3.97 (m, 1H), 3.89-3.71 (m, 1H), 3.44 (m, 1H), 2.53 (s, 3H), 2.32 (s, 3H). **^{13}C NMR** (101 MHz, $CDCl_3$) δ 140.2 (d, J = 7.5 Hz), 137.8, 137.5, 137.1, 135.9, 133.2, 133.1, 132.6, 132.4, 132.1, 131.8, 131.7, 131.6, 131.4, 131.2, 130.9, 130.34 (d, J = 15.8 Hz), 130.3, 129.56 (d, J = 5.1 Hz), 129.6, 129.4, 129.0, 128.8, 128.7, 128.2, 128.0, 127.8, 127.7, 127.3, 127.1, 126.8, 126.1, 125.7, 125.6, 43.6, 43.0, 35.4, 21.4, 21.0. **^{31}P NMR** (162 MHz, $CDCl_3$) δ 33.47 (s). **HRMS (ESI):** ($[M+H]^+$) Calcd for $C_{44}H_{38}OPS^+$: 645.2381, Found: 645.2379. **IR** (film) ν 3581, 2973, 2921, 1677, 1539, 1479, 1241, 1109, 1027, 939, 741, 689 cm^{-1}.

6.2. products of dendralenes

(*E*)-(1-(1-benzylidene-3-phenyl-1H-inden-2-yl)vinyl)diphenylphosphine oxide (2a)

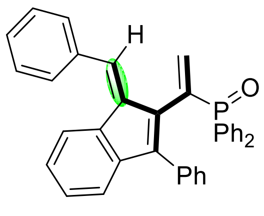

2a

A yellow solid, (42 mg, 83% yield), *m.p.*: 137.4-139.1 °C. **^{1}H NMR** (400 MHz, $CDCl_3$) δ 7.50 (m, 4H), 7.45-7.32 (m, 9H), 7.29-7.22 (m, 7H), 7.18-7.09 (m, 2H), 7.07 (s, 1H), 6.97 (t, J = 7.3 Hz, 1H), 6.72 (m, 1H), 6.46 (m, 1H). **^{13}C NMR** (101 MHz, $CDCl_3$) δ 143.7, 141.9, 141.1, 139.7, 137.4, 136.5, 135.4, 134.4, 133.9, 131.9 (d, J = 9.6 Hz), 131.5 (d, J = 2.7 Hz), 130.5, 129.4 (d, J = 13.1 Hz), 128.3, 128.2, 128.1, 128.0, 127.8, 127.7, 125.5, 123.1, 120.2. **^{31}P NMR** (162 MHz, $CDCl_3$) δ 26.04 (s). **HRMS (ESI):** ($[M+H]^+$) Calcd for $C_{36}H_{29}OP^+$: 507.1878, Found: 507.1876. **IR** (film) ν 3677, 2985, 2907, 2221, 1592, 1574, 1496, 1438, 1172, 1077, 916, 739, 695 cm^{-1}.

(*E*)-(1-(1-(4-fluorobenzylidene)-3-phenyl-1H-inden-2-yl)vinyl)diphenylphosphine oxide (2b)

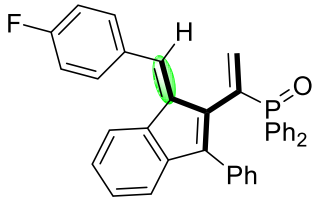

2b

A yellow solid, (37 mg, 70% yield), *m.p.*: 107.4-109.1 °C. **^{1}H NMR** (400 MHz, $CDCl_3$) δ 7.53-7.43 (m, 8H), 7.43-7.32 (m, 12H), 7.28-7.20 (m, 16H), 7.19-7.04 (m, 8H), 7.02-6.95 (m, 4H), 6.72 (d, J = 1.6 Hz, 1H), 6.67 (d, J = 1.6 Hz, 1H), 6.49 (d, J = 1.6 Hz, 1H), 6.40 (d, J = 1.6 Hz, 1H). **^{13}C NMR** (101 MHz, $CDCl_3$) δ 161.4), 143.7, 142.0, 141.2, 140.6 139.7, 137.4, 135.3, 134.25 (d, J = 11.1 Hz), 133.7, 132.19 (d, J = 48.1 Hz), 132.0, 131.9, 131.6, 131.5, 131.2, 131.1, 130.4, 129.4, 128.3, 128.1, 128.0, 127.9, 127.8, 125.5, 122.9, 120.3, 115.5, 115.2, 100.0. **^{31}P NMR** (162 MHz, $CDCl_3$) δ 26.15 (s). **HRMS (ESI):** ($[M+H]^+$) Calcd for $C_{36}H_{26}FOP$: 525.1784, Found: 525.1783. **IR** (film) ν 3677, 2985, 2900, 2213, 1502, 1396, 1245, 1061, 924, 723, 694 cm^{-1}.

(*E*)-(1-(1-(4-chlorobenzylidene)-3-phenyl-1H-inden-2-yl)vinyl)diphenylphosphine oxide (2c)

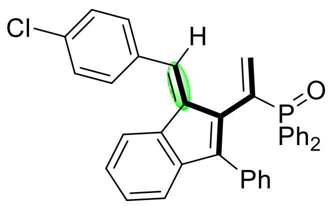

2c

A yellow solid, (41 mg, 76% yield), *m.p.*: 111.6-113.7 °C. **^{1}H NMR** (400 MHz, $CDCl_3$) δ 7.54-7.31 (m, 26H), 7.22 (m, 16H), 7.13 (m, 4H), 6.98 (m, 4H), 6.73 (s, 1H), 6.69 (s, 1H), 6.49 (s, 1H), 6.40 (s, 1H). **^{13}C NMR** (101 MHz, $CDCl_3$) δ 143.7, 141.6, 134.9, 134.1, 133.7 (d, J = 17.0 Hz), 131.9 (d, J = 9.6 Hz), 131.6, 130.7, 129.4, 128.5, 128.3, 128.2, 128.1, 128.0, 127.9, 125.6, 122.9, 120.4. **^{31}P NMR** (162 MHz, $CDCl_3$) δ 26.47 (s). **HRMS (ESI):** ($[M+H]^+$) Calcd for $C_{36}H_{27}ClOP^+$: 541.1488, Found: 537.2322. **IR** (film), ν 3059, 2947, 2837, 2385, 1544, 1423, 1422, 1343, 1023, 881, 735, 675 cm^{-1}.

(*E*)-4-((2-(1-(diphenylphosphoryl)vinyl)-3-phenyl-1H-inden-1-ylidene)methyl)benzonitrile (2d)

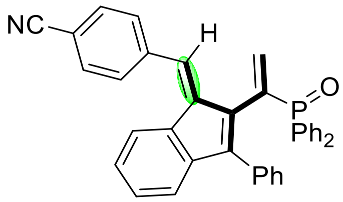

2d

A yellow solid, (42 mg, 79% yield), *m.p.*: 125.7-127.6 °C. **^{1}H NMR** (400 MHz, $CDCl_3$) δ 7.68 (d, J = 8.2 Hz, 4H), 7.55-7.32 (m, 24H), 7.31-7.20 (m, 15H), 7.20-7.06 (m, 4H), 7.02-6.94 (m, 4H), 6.67 (d, J = 1.3 Hz, 1H), 6.62 (d, J = 1.3 Hz, 1H), 6.49 (d, J = 1.4 Hz, 1H), 6.39 (d, J = 1.4 Hz, 1H). **^{13}C NMR** (101 MHz, $CDCl_3$) δ 143.9, 143.0, 141.5, 137.8, 134.0, 133.3, 132.3, 132.1, 131.9, 131.8, 131.7, 131.6, 131.3, 131.3, 130.3, 130.0, 129.3, 128.45 (d, J = 20.2 Hz), 128.13 (d, J = 12.0 Hz), 125.9, 122.9, 120.6, 118.8, 111.6, 100.0. **^{31}P NMR** (162 MHz, $CDCl_3$) δ 26.27 (s). **HRMS (ESI):** ($[M+H]^+$) Calcd for $C_{37}H_{27}NOP^+$: 532.1830, Found: 532.1830. **IR** (film) ν 3675, 2970, 2906, 2231, 1537, 1338, 1244, 1044, 891, 694 cm^{-1}.

(*E*)-(1-(1-(4-nitrobenzylidene)-3-phenyl-1H-inden-2-yl)vinyl)diphenylphosphine oxide (2e)

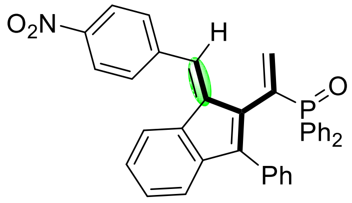

2e

A yellow solid, (49 mg, 90% yield), *m.p.*: 186.3-188.0 °C. **^{1}H NMR** (400 MHz, $CDCl_3$) δ 7.67 (d, *J* = 7.5 Hz, 2H), 7.63 – 7.47 (m, 12H), 7.45 – 7.26 (m, 10H), 7.17 (s, 5H), 6.14 (d, *J* = 12.1 Hz, 1H), 6.05 (d, *J* = 12.1 Hz, 1H), 5.89 (d, *J* = 42.0 Hz, 1H), 5.46 (d, *J* = 19.4 Hz, 1H). **^{13}C NMR** (101 MHz, $CDCl_3$) δ 145.2 (d, *J* = 7.5 Hz), 143.4 (d, *J* = 94.9 Hz), 142.4, 142.1, 140.9, 139.7, 136.0, 135.5 (d, *J* = 10.1 Hz), 133.7 (d, *J* = 7.2 Hz), 133.0, 132.7, 131.9, 131.8, 131.5 (d, *J* = 2.6 Hz), 130.9, 130.0, 129.8, 128.9, 128.3, 128.2, 127.9, 127.6, 127.3 (d, *J* = 8.1 Hz), 127.0, 126.9, 126.3. **^{31}P NMR** (162 MHz, $CDCl_3$) δ 28.67 (s). **HRMS (ESI):** ($[M+H]^+$) Calcd for $C_{42}H_{34}OP^+$: 585.2342, Found: 585.2341. **IR** (film) ν 3062, 3015, 1599, 1487, 1435, 1195, 1111, 966, 860, 690 cm^{-1}.

Methyl-(E)-4-((2-(1-(diphenylphosphoryl)vinyl)-3-phenyl-1H-inden-1-ylidene)methyl)benzoate (2f)

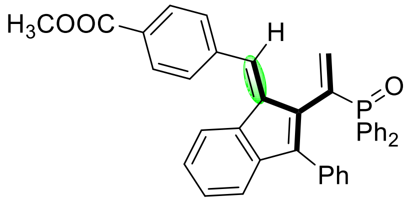

2f

A yellow solid, (48 mg, 85% yield), *m.p.*: 185.5-187.4 °C. **^{1}H NMR** (400 MHz, $CDCl_3$) δ 8.06 (d, J = 8.2 Hz, 4H), 7.46 (M, 13H), 7.35 (M, 9H), 7.23 (t, J = 7.9 Hz, 9H), 7.13 (M, 5H), 7.07-6.92 (m, 5H), 6.76 (s, 1H), 6.71 (s, 1H), 6.54 (d, J = 4.6 Hz, 1H), 6.45 (s, 1H), 3.97 (s, 3H). **^{13}C NMR** (101 MHz, $CDCl_3$) δ 152.2, 152.1, 132.3, 132.2, 131.9, 131.8, 131.7, 130.3 (d, *J* = 7.0 Hz), 130.2, 128.4, 128.3, 128.2, 128.0, 127.9, 127.8, 127.6, 89.5, 21.2. **^{31}P NMR** (162 MHz, $CDCl_3$) δ 27.80 (s). **HRMS (ESI):** ($[M+H]^+$) Calcd for $C_{37}H_{32}O_2P^+$:$C_{38}H_{30}O_3P$:565.1933, Found: 565.1931. **IR** (film) ν 3676, 2985, 2901, 1605, 1524, 1455, 1255, 1108, 1021, 945, 765, 696 cm^{-1}.

Diphenyl(1-(1-phenyl-4-(m-tolyl)naphthalen-2-yl)vinyl)phosphine oxide (3g)

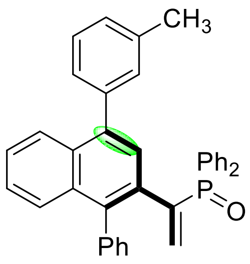

3g

A yellow solid, (42 mg, 80% yield), *m.p.*: 179.5-181.6 °C. **^{1}H NMR** (400 MHz, $CDCl_3$) δ 7.97 (d, J = 8.1 Hz, 1H), 7.62-7.52 (m, 7H), 7.40 (m, 16H), 7.25-6.99 (m, 14H), 6.00 (m, 1H), 5.93 (d, J = 8.2 Hz, 1H), 2.42 (s, 3H). **^{13}C NMR** (101 MHz, $CDCl_3$) δ 143.8, 139.0, 138.5, 137.2, 137.0, 134.8 133.6, 133.2, 133.1, 132.4, 132.3 (d, *J* = 2.7 Hz), 131.9, 131.8, 131.4, 131.1, 130.8, 130.0, 128.9, 128.5, 128.3, 127.8, 127.5, 127.3, 127.0, 125.9, (d, *J* = 5.1 Hz), 21.3. **^{31}P NMR** (162 MHz, $CDCl_3$) δ 28.58 (s). **HRMS (ESI):** ($[M+H]^+$) Calcd for $C_{37}H_{30}OP^+$: 521.2034, Found: 521.2033. **IR** (film) ν 3645, 3408, 3235, 2985, 2917, 1634, 1594, 1435, 1245, 1093, 834, 758, 695 cm^{-1}.

Diphenyl(1-(1-phenyl-4-(p-tolyl)naphthalen-2-yl)vinyl)phosphine oxide (3h)

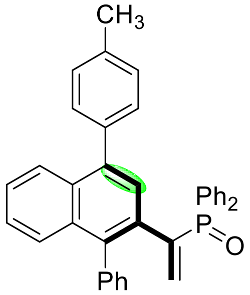

3h

A yellow solid, (39 mg, 75% yield), *m.p.*: 167.3-168.8 °C. **^{1}H NMR** (400 MHz, $CDCl_3$) δ 7.98 (d, J = 8.1 Hz, 1H), 7.67-7.47 (m, 7H), 7.46-7.32 (m, 9H), 7.30-7.21 (m, 4H), 7.17 (dd, J = 6.4, 3.0 Hz, 2H), 7.11 (s, 1H), 6.09-5.96 (m, 1H), 5.93 (s, 1H), 2.47 (s, 3H). **^{13}C NMR** (101 MHz, $CDCl_3$) δ 144.7, 143.8, 139.0 (s), 138.7 (d, J = 47.4 Hz), 137.2, 137.0, 134.8, 133.6, 133.2 (d, J = 8.5 Hz), 132.3 (d, J = 9.6 Hz), 131.9 (d, J = 2.7 Hz), 131.4, 131.1, 130.8, 130.0, 128.9 128.4 (d, J = 12.0 Hz), 127.8, 127.4 (d, J = 16.2 Hz), 127.0, 125.9 (d, J = 5.6 Hz), 21.3. **^{31}P NMR** (162 MHz, $CDCl_3$) δ 28.17 (s), **HRMS (ESI):** ($[M+H]^+$) Calcd for $C_{37}H_{30}OP^+$: 521.2034, Found: 521.2039. **IR** (film) ν 3645, 3051, 2968, 2900, 2228, 1603, 1503, 1432, 1177, 950, 865, 694 cm^{-1}.

(1-(4-(4-methoxyphenyl)-1-phenylnaphthalen-2-yl)vinyl)Diphenylphosphine oxide (3i)

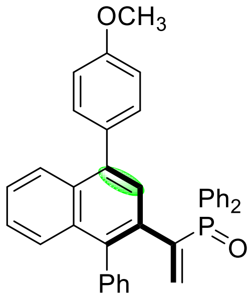

3i

A yellow solid, (45 mg, 84% yield), *m.p.*: 181.5-183.4 °C. **^{1}H NMR** (400 MHz, $CDCl_3$) δ 7.98 (d, J = 8.3 Hz, 1H), 7.68-7.46 (m, 7H), 7.46-7.31 (m, 9H), 7.30-7.22 (m, 2H), 7.15 (dd, J = 6.4, 3.0 Hz, 2H), 7.09 (s, 1H), 6.99 (d, J = 8.6 Hz, 2H), 6.01 (d, J = 22.2 Hz, 1H), 5.93 (s, 1H), 3.90 (s, 3H). **^{13}C NMR** (101 MHz, $CDCl_3$) δ 159.0, 144.4, 138.7, 138.5, 135.1, 133.6, 133.1, 132.5, 132.3, 132.2, 132.0 (d, J = 2.7 Hz), 131.5 (d, J = 10.3 Hz), 131.1, 130.5, 128.4 (d, J = 12.1 Hz), 127.8, 127.4 (d, J = 18.5 Hz), 127.0, 125.9, 113.7, 55.4. **^{31}P NMR** (162 MHz, $CDCl_3$) δ 28.86 (s). **HRMS (ESI):** ($[M+H]^+$) Calcd for $C_{37}H_{30}O_2P$: 537.1983, Found: 537.1986. **IR** (film) ν 3365, 2765, 2656, 2344, 1581, 1492, 1488, 1385, 1055, 883, 745, 635 cm^{-1}.

(1-(4-(4-(tert-butyl)phenyl)-1-phenylnaphthalen-2-yl)vinyl)diphenylphosphine oxide(3k)

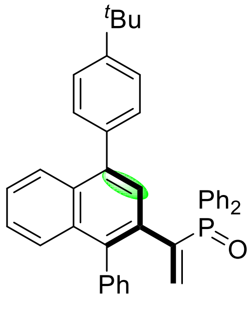

3k

A yellow solid, (39 mg, 70% yield), *m.p.*: 195.2-197.7 °C. ^{1}H NMR (400 MHz, $CDCl_3$) δ 8.01 (d, J = 8.3 Hz, 1H), 7.55 (m, 6H), 7.52-7.32 (m, 13H), 7.33-7.24 (m, 4H), 7.13 (m, 4H), 5.95 (dd, J = 29.9, 16.8 Hz, 2H), 1.43 (s, 9H). **^{13}C NMR** (101 MHz, $CDCl_3$) δ 150.2, 144.8, 138.9, 138.5, 137.1, 134.8, 133.6, 132.27 (d, J = 9.7 Hz), 131.8, 131.4, 131.0, 129.8, 128.4 (d, J = 12.0 Hz), 127.7, 127.4, 127.0, 126.0 (d, J = 18.2 Hz), 125.1, 124.7, 34.6, 31.6,31.5. **^{31}P NMR** (162 MHz, $CDCl_3$) δ 28.82 (s). **HRMS (ESI):** ($[M+H]^+$) Calcd for $C_{40}H_{36}OP^+$: 563.2504, Found: 563.2501. **IR** (film) ν 3012, 2945, 2832, 2375, 1545, 1423, 1425, 1315, 1054, 945, 738, 645 cm^{-1}.

(1-(4-([1,1'-biphenyl]-4-yl)-1-phenylnaphthalen-2-yl)vinyl)diphenylphosphine oxide (3l)

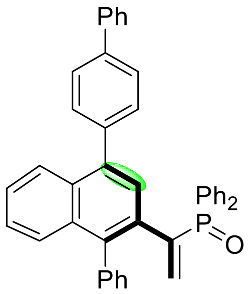

3l

A yellow solid, (44 mg, 75% yield), *m.p.*: 112.1-113.3 °C. **^{1}H NMR** (400 MHz, $CDCl_3$) δ 8.04 (d, J = 8.3 Hz, 1H), 7.70 (m, 4H), 7.57 (m, 9H), 7.48-7.34 (m, 12H), 7.22-7.11 (m, 3H), 6.00 (d, J = 25.8 Hz, 1H), 5.92 (d, J = 4.3 Hz, 1H). **^{13}C NMR** (101 MHz, $CDCl_3$) δ 140.8, 140.1, 139.2, 138.9,138.8, 138.5 (d, J = 11.0 Hz), 134.9 (d, J = 9.2 Hz), 133.7, 133.3, 133.2, 132.4,132.3, 131.9,131.8, 131.4, 131.0, 130.53 (s), 130.8, 128.9, 128.5, 128.4, 127.8, 127.6,127.5, 127.2, 127.1, 126.9, 126.1, 126.0, 125.8. **^{31}P NMR** (162 MHz, $CDCl_3$) δ 28.27 (s). **HRMS (ESI):** ([M+H]$^+$) Calcd for $C_{42}H_{32}OP^+$: 583.2191, Found: 583.2195. **IR** (film) ν 3668, 2983, 2902, 2214, 1605, 1401, 1254, 1065, 894, 693 cm^{-1}.

Diphenyl(1-(1-phenyl-4-(pyridin-3-yl)naphthalen-2-yl)vinyl)phosphine oxide (3m)

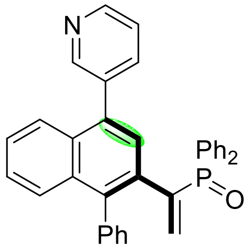

3m

A yellow solid, (30 mg, 60% yield), *m.p.*: 146.2-148.5 °C. **^{1}H NMR** (400 MHz, $CDCl_3$) δ 8.40 (s, 1H), 8.00-7.60 (m, 5H), 7.59-6.82 (m, 18H), 5.97 (dd, J = 54.8, 28.6 Hz, 2H). **^{13}C NMR** (101 MHz, $CDCl_3$) δ 150.8, 141.2, 140.2, 138.7, 132.1 (d, J = 10.0 Hz), 131.0, 130.2 (d, J = 18.0 Hz), 128.5, 128.4, 128.0, 127.8, 127.7, 126.7, 123.0, 100.0, 96.4. **^{31}P NMR** (162 MHz, $CDCl_3$) δ 28.62 (s). **HRMS (ESI):** ([M+H]$^+$) Calcd for $C_{35}H_{27}NOP^+$: 508.1830, Found: 508.1835. **IR** (film) ν 3653, 3382, 2933, 2223, 1548, 1435, 1173, 957, 795, 695 cm^{-1}.

Diphenyl(1-(1-phenyl-4-(thiophen-3-yl)naphthalen-2-yl)vinyl)phosphine oxide (3n)

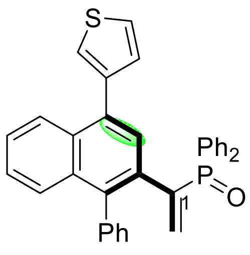

3n

A yellow solid, (33 mg, 65% yield), *m.p.*: 173.2-174.7 °C. **^{1}H NMR** (400 MHz, $CDCl_3$) δ 8.08 (d, J = 8.4 Hz, 1H), 7.60-7.49 (m, 5H), 7.48-7.33 (m, 9H), 7.20 (M, 1H), 7.14 (M, 3H), 5.97 (dd, J = 30.5, 20.9 Hz, 2H). **^{13}C NMR** (101 MHz, $CDCl_3$) δ 140.5, 139.0, 138.2, 135.6, 133.7 (d, J = 29.3 Hz), 132.4, 132.3, 132.1, 131.3, 131.2, 129.6, 128.6, 128.5, 128.4, 127.8, 127.7, 127.6, 127.4, 127.1, 127.0, 126.2, 126.1, 126.0, 125.7, 125.5, 125.3, 123.7. **^{31}P NMR** (162 MHz, $CDCl_3$) δ 30.37 (s). **HRMS (ESI):** ($[M+H]^+$) Calcd for $C_{34}H_{26}OPS^+$: 513.1442, Found: 513.1447. **IR** (film) ν 3675, 3050, 2223, 1678, 1585, 1437, 1314, 126.6, 117.03, 112.7, 903, 853 cm^{-1}.

7. ^{1}H-NMR, ^{13}C-NMR, ^{31}P-NMR and HRMS spectra

2P'

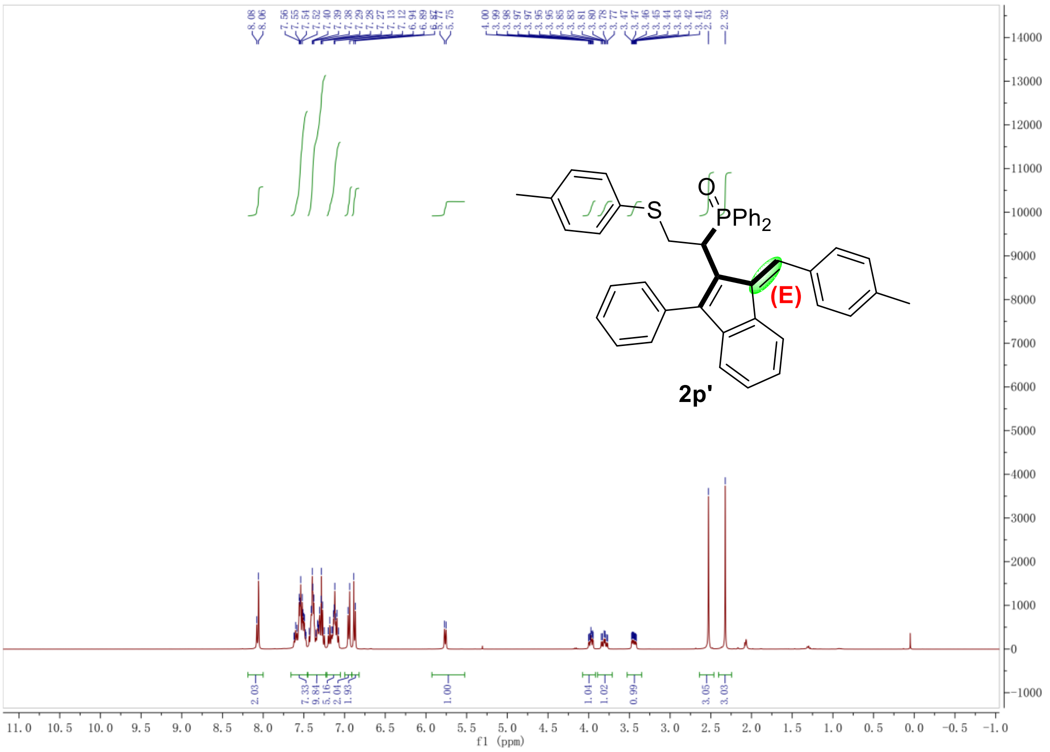

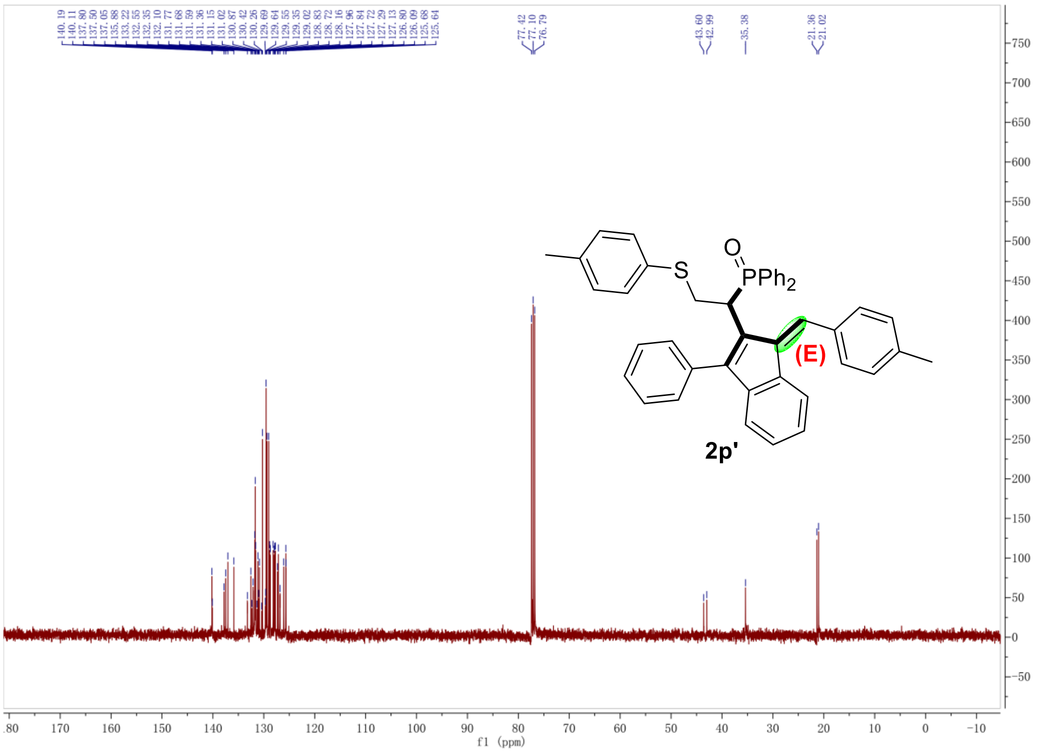
140.19
140.11
137.80
137.50
137.05
135.88
133.22
132.55
132.35
132.10
131.77
131.68
131.59
131.36
131.15
131.02
130.87
130.42
130.26
129.69
129.64
129.55
129.35
129.02
128.83
128.72
128.16
127.96
127.84
127.72
127.29
127.13
126.80
126.09
125.68
125.64
77.42
77.10
76.79
43.60
42.99
35.38
21.36
21.02
O
PPh2
S
(E)
2p'
f1 (ppm)

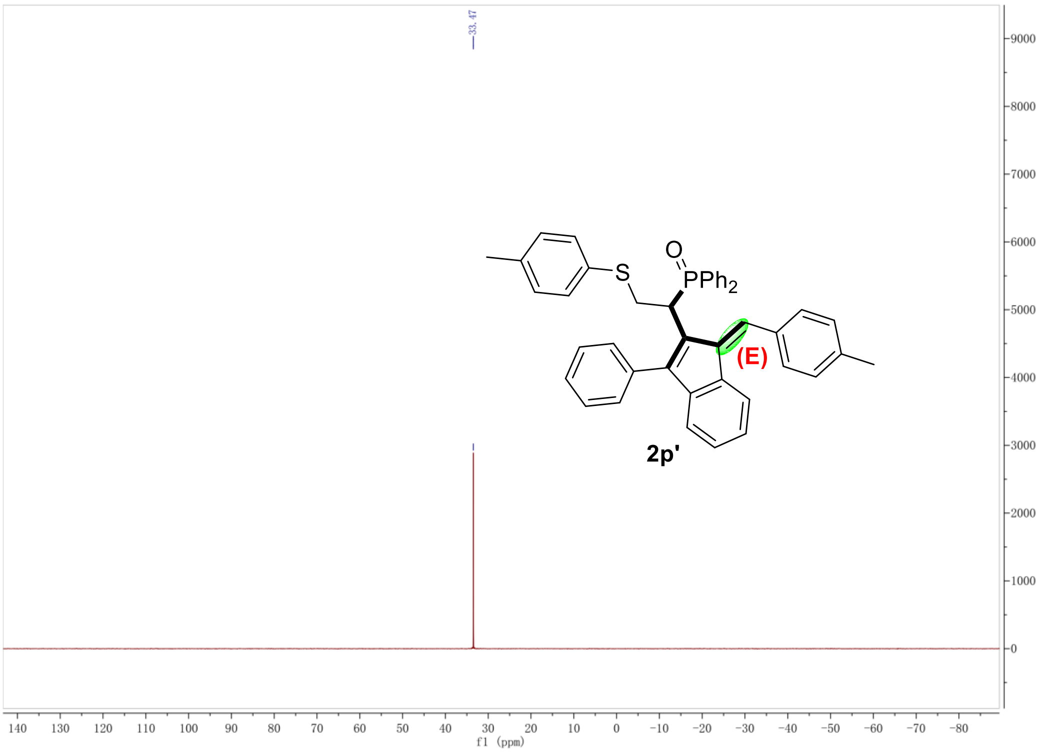
33.47
O
PPh2
S
(E)
2p'
f1 (ppm)

2a

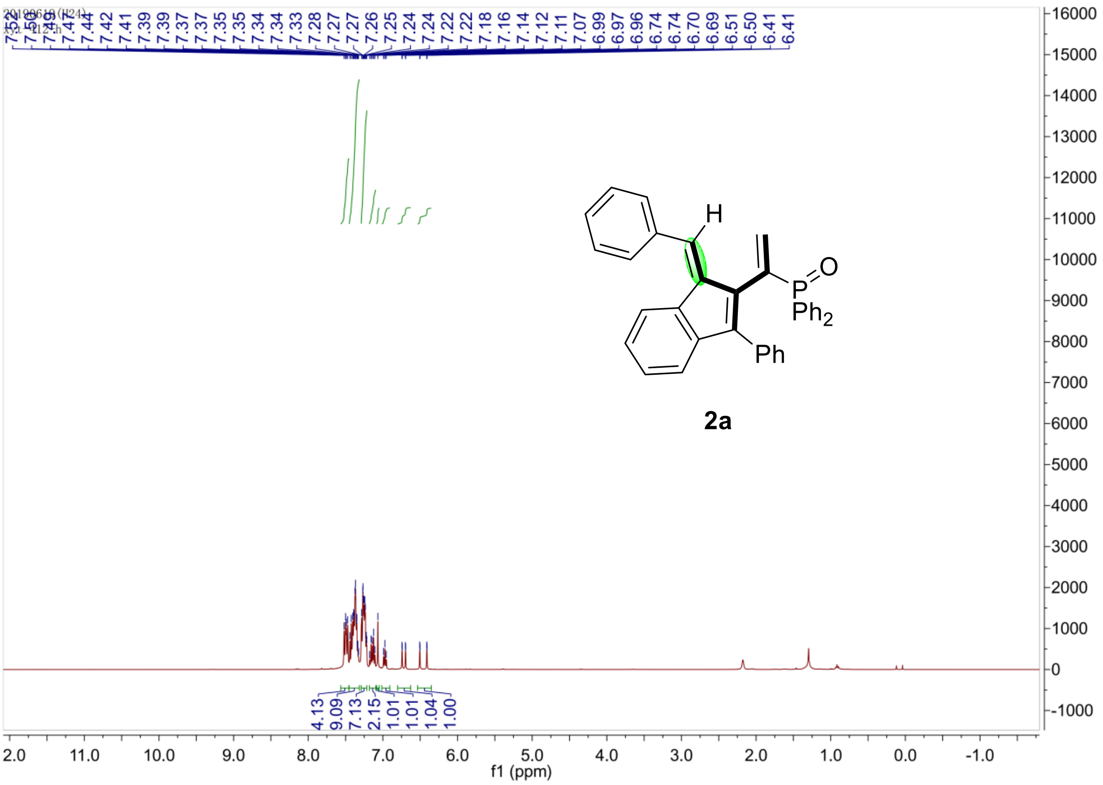
7.44
7.42
7.41
7.39
7.39
7.37
7.37
7.35
7.35
7.34
7.34
7.33
7.28
7.27
7.27
7.26
7.25
7.24
7.24
7.22
7.22
7.18
7.16
7.14
7.12
7.11
7.07
6.99
6.97
6.96
6.74
6.74
6.70
6.69
6.51
6.50
6.41
6.41
4.13
9.09
7.13
2.15
1.01
1.01
1.04
1.00
H
P
O
Ph2
Ph
2a
f1 (ppm)

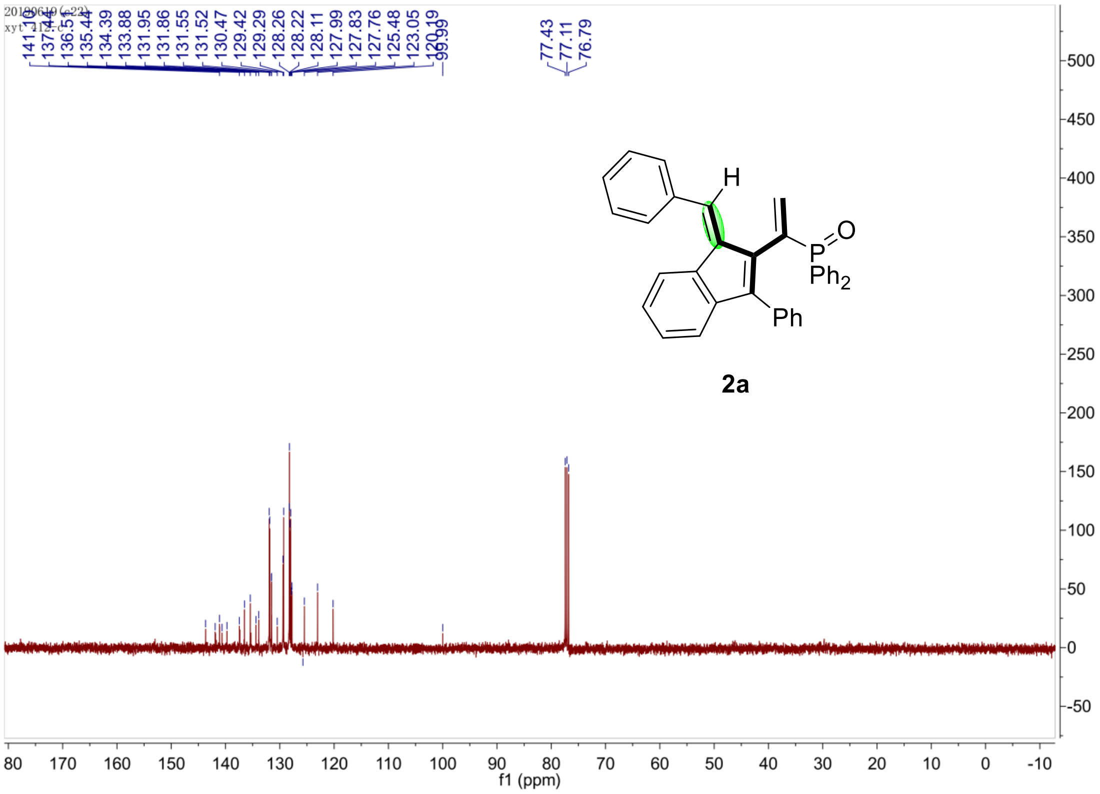
141.10
137.44
136.53
135.44
134.39
133.88
131.95
131.86
131.55
131.52
130.47
129.42
129.29
128.26
128.22
128.11
127.99
127.83
127.76
125.48
123.05
120.19
99.99
77.43
77.11
76.79
H
P
O
Ph2
Ph
2a
f1 (ppm)

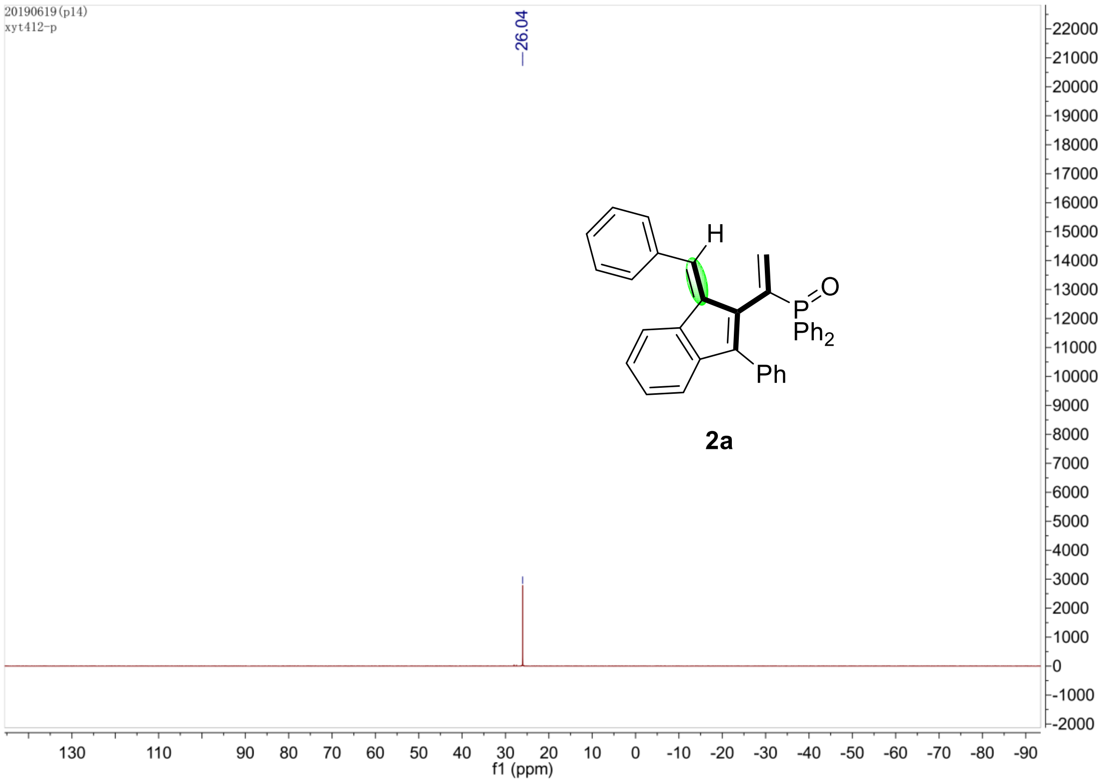
20190619(p14)
xyt412-p
26.04
H
P=O
Ph2
Ph
2a
f1 (ppm)

2b

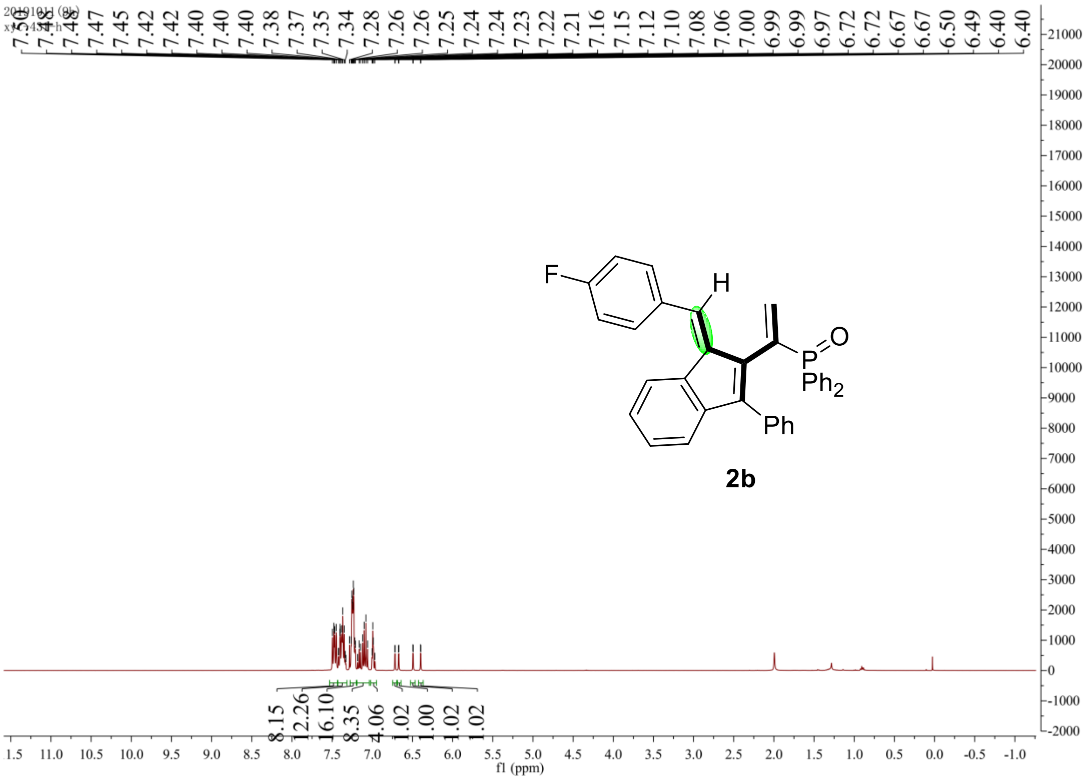
7.50 7.48 7.48 7.47 7.45 7.42 7.42 7.40 7.40 7.40 7.38 7.37 7.35 7.34 7.28 7.26 7.26 7.25 7.24 7.24 7.23 7.22 7.21 7.16 7.15 7.12 7.10 7.08 7.06 7.00 6.99 6.99 6.97 6.72 6.72 6.67 6.67 6.50 6.49 6.40 6.40
F
H
P=O
Ph2
Ph
2b
8.15 12.26 16.10 8.35 4.06 1.02 1.00 1.02 1.02
f1 (ppm)

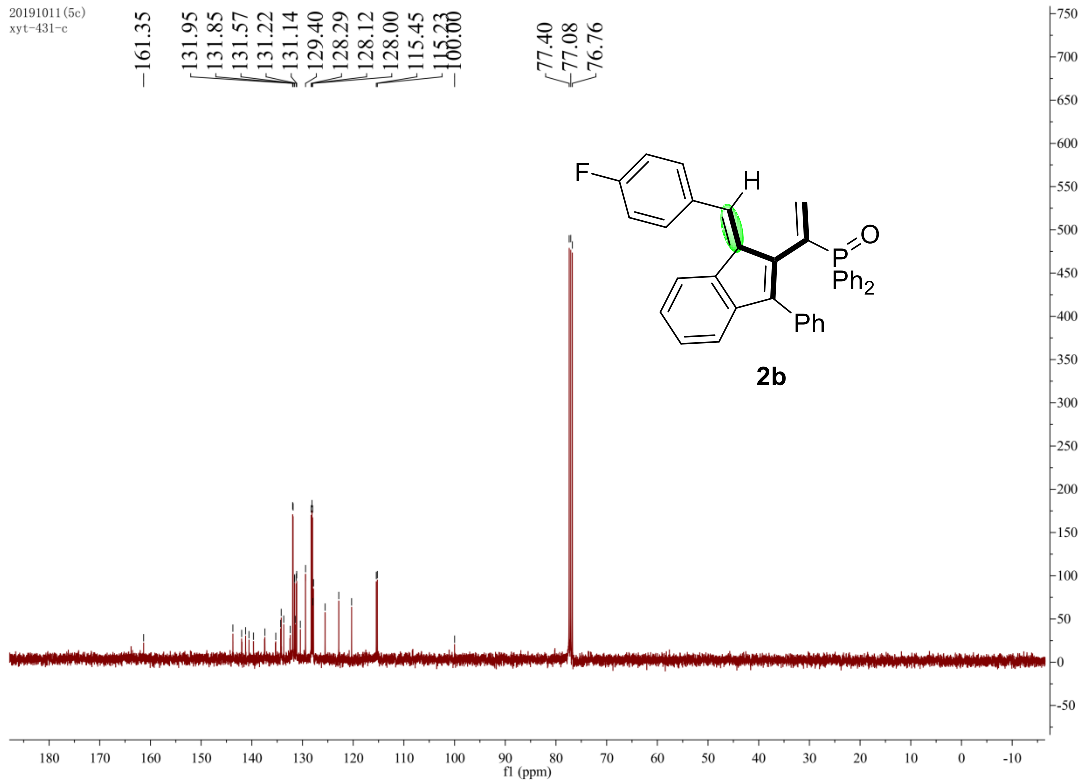
20191011(5c)
xyt-431-c
161.35
131.95
131.85
131.57
131.22
131.14
129.40
128.29
128.12
128.00
115.45
115.23
100.00
77.40
77.08
76.76
F
H
P
O
Ph2
Ph
2b
fl (ppm)

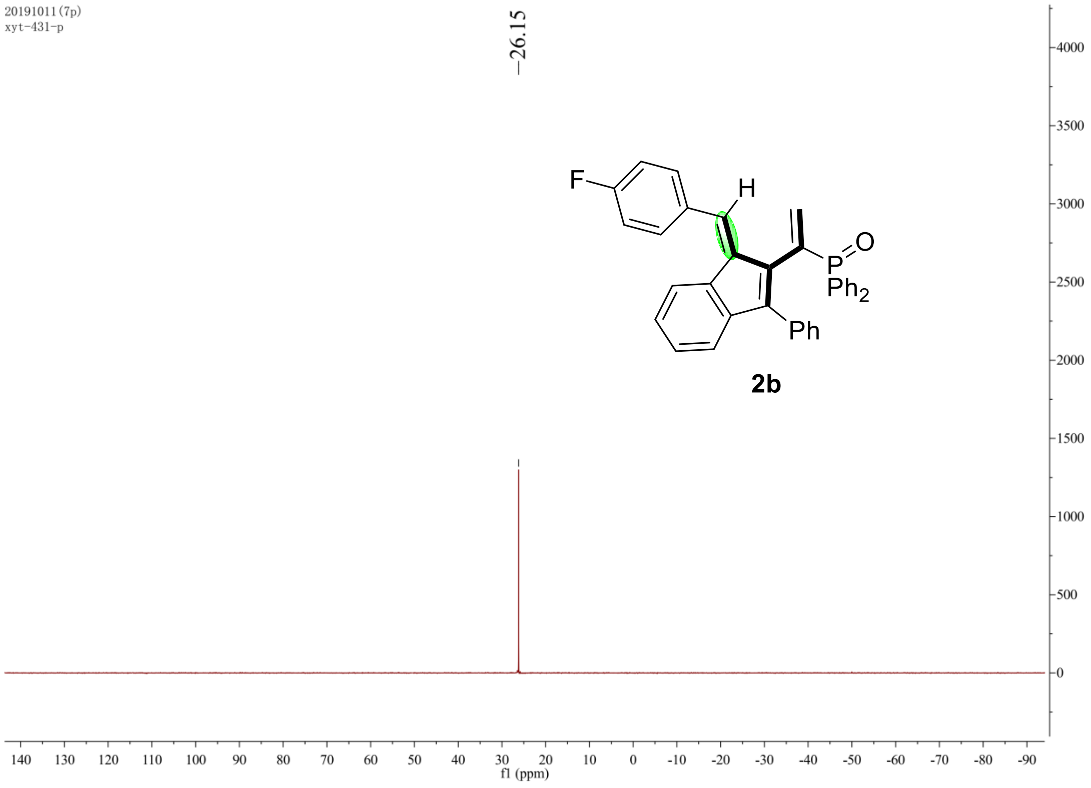
20191011(7p)
xyt-431-p
26.15
F
H
P
O
Ph2
Ph
2b
fl (ppm)

2c

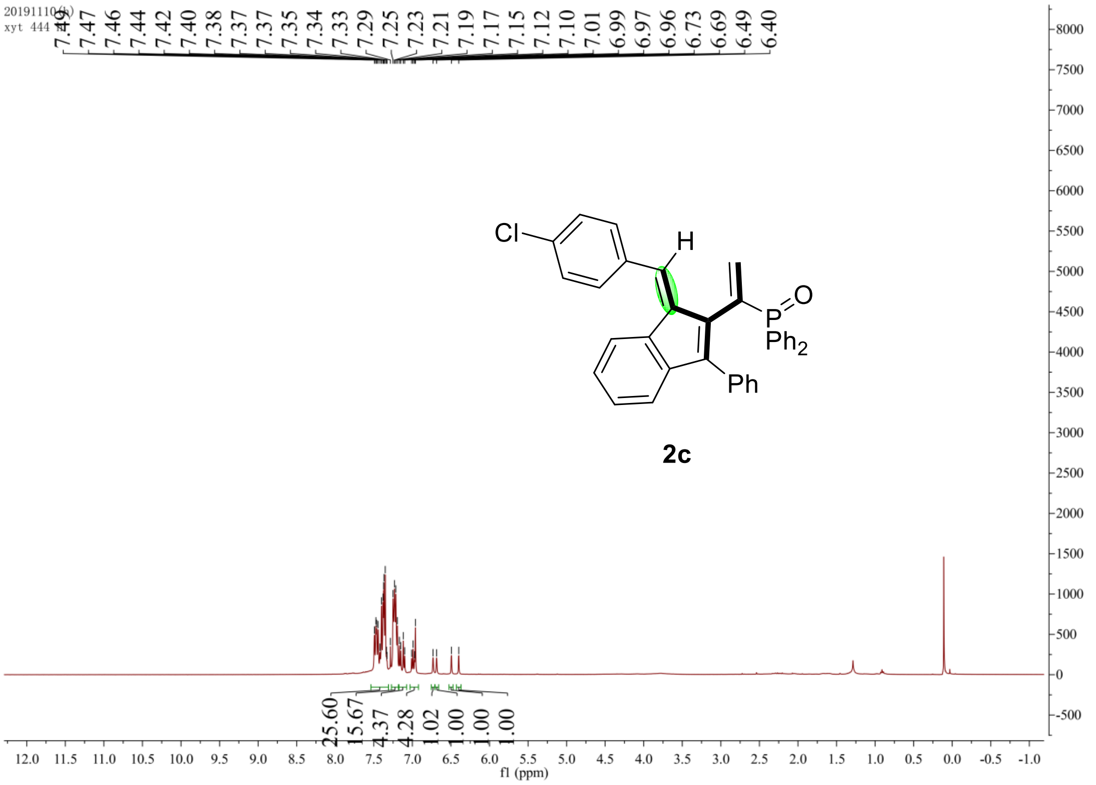
7.49 7.47 7.46 7.44 7.42 7.40 7.38 7.37 7.37 7.35 7.34 7.33 7.29 7.25 7.23 7.21 7.19 7.17 7.15 7.12 7.10 7.01 6.99 6.97 6.96 6.73 6.69 6.49 6.40
25.60 15.67 4.37 4.28 1.02 1.00 1.00 1.00
Cl
H
P=O
Ph2
Ph
2c
fl (ppm)

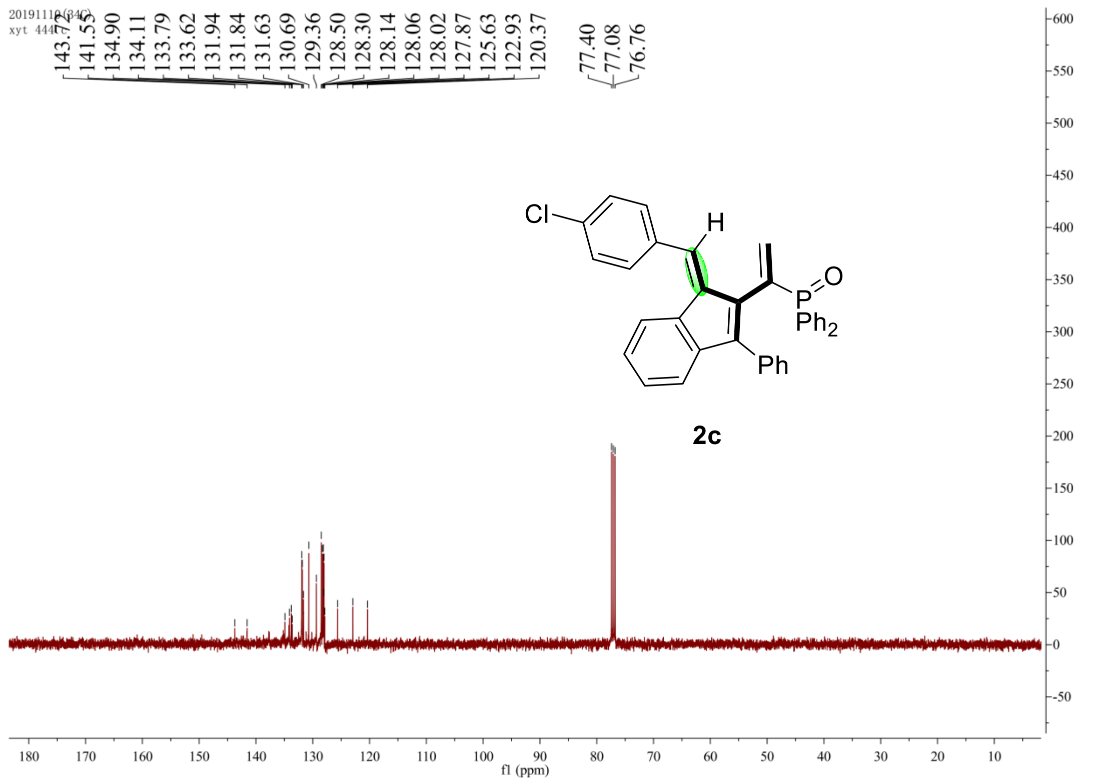
134.90 134.11 133.79 133.62 131.94 131.84 131.63 130.69 129.36 128.50 128.30 128.14 128.06 128.02 127.87 125.63 122.93 120.37
77.40 77.08 76.76
Cl
H
P=O
Ph2
Ph
2c
fl (ppm)

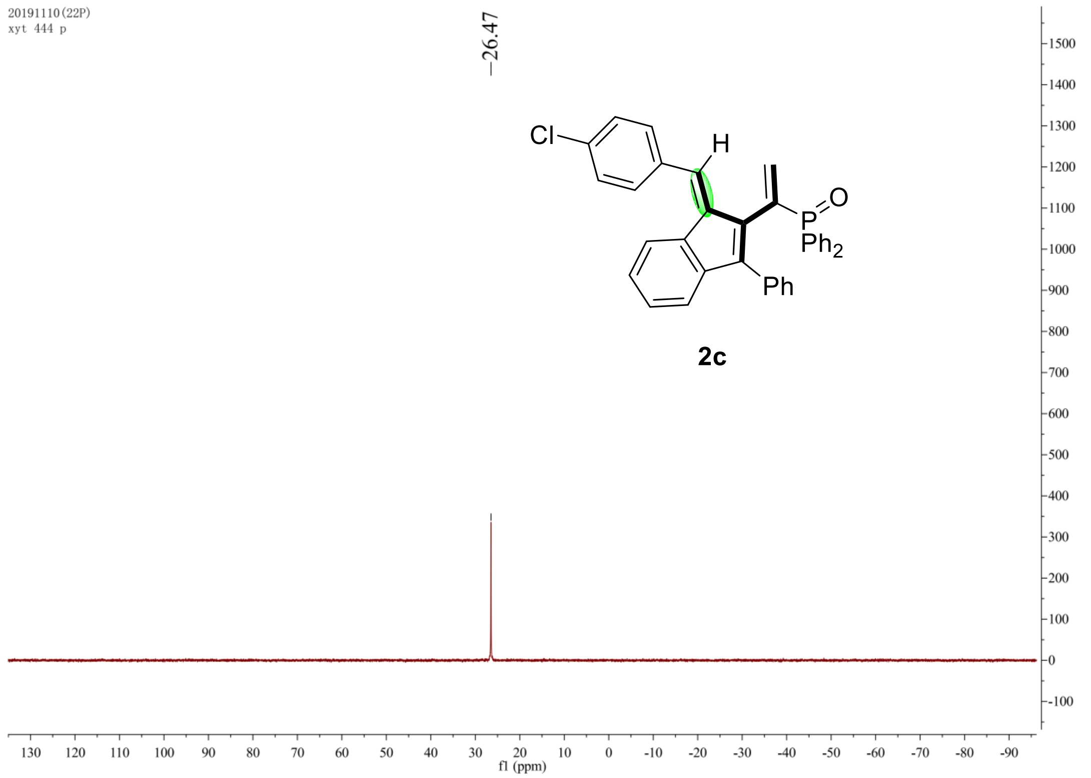
20191110(22P)
xyt 444 p
26.47
Cl
H
O
P
Ph2
Ph
2c
f1 (ppm)

2d

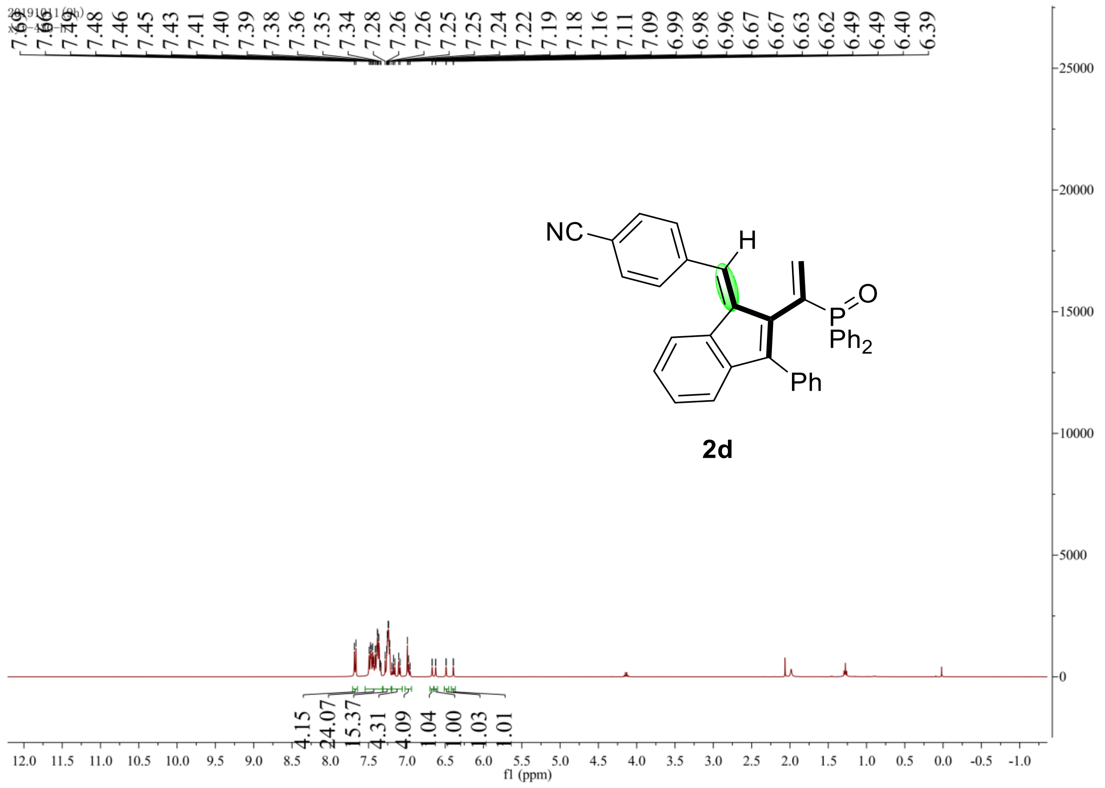
7.48
7.46
7.45
7.43
7.41
7.40
7.39
7.38
7.36
7.35
7.34
7.28
7.26
7.26
7.25
7.25
7.24
7.22
7.19
7.18
7.16
7.11
7.09
6.99
6.98
6.96
6.67
6.67
6.63
6.62
6.49
6.49
6.40
6.39
NC
H
O
P
Ph2
Ph
2d
4.15
24.07
15.37
4.31
4.09
1.04
1.00
1.03
1.01
f1 (ppm)

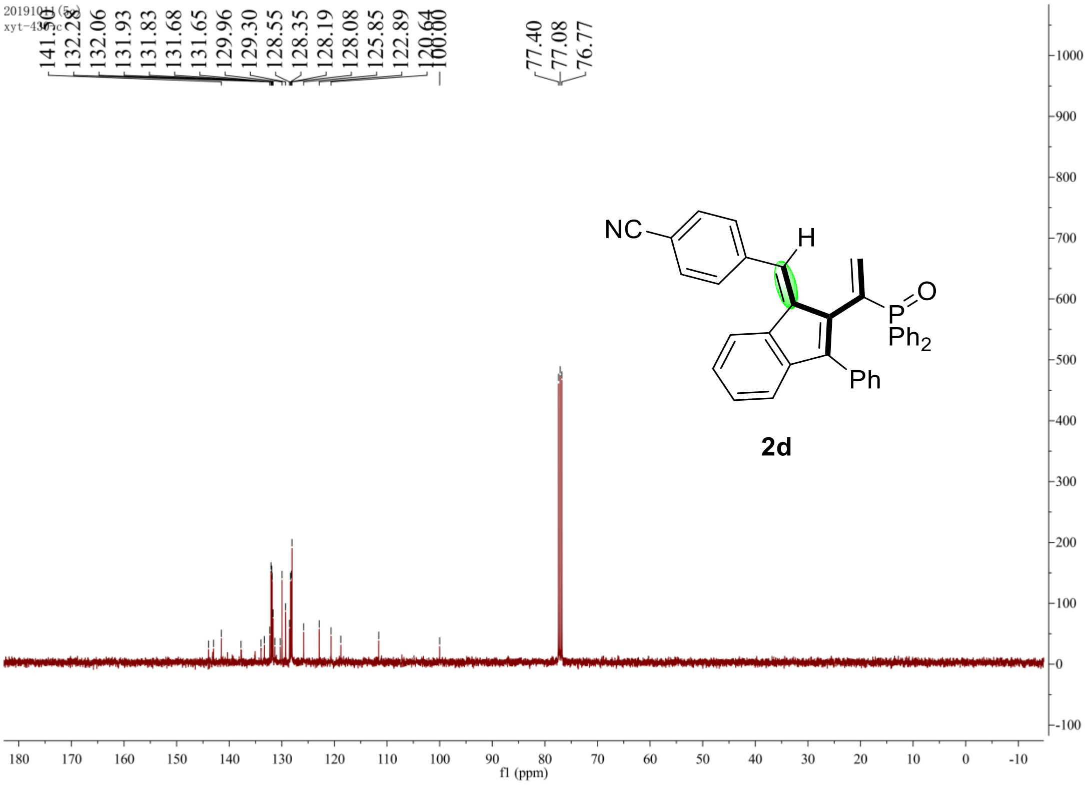
20191011(5c)
xyt-430-c
141.50
132.28
132.06
131.93
131.83
131.68
131.65
129.96
129.30
128.55
128.35
128.19
128.08
125.85
122.89
120.64
100.00
77.40
77.08
76.77
NC
H
P
O
Ph2
Ph
2d
f1 (ppm)

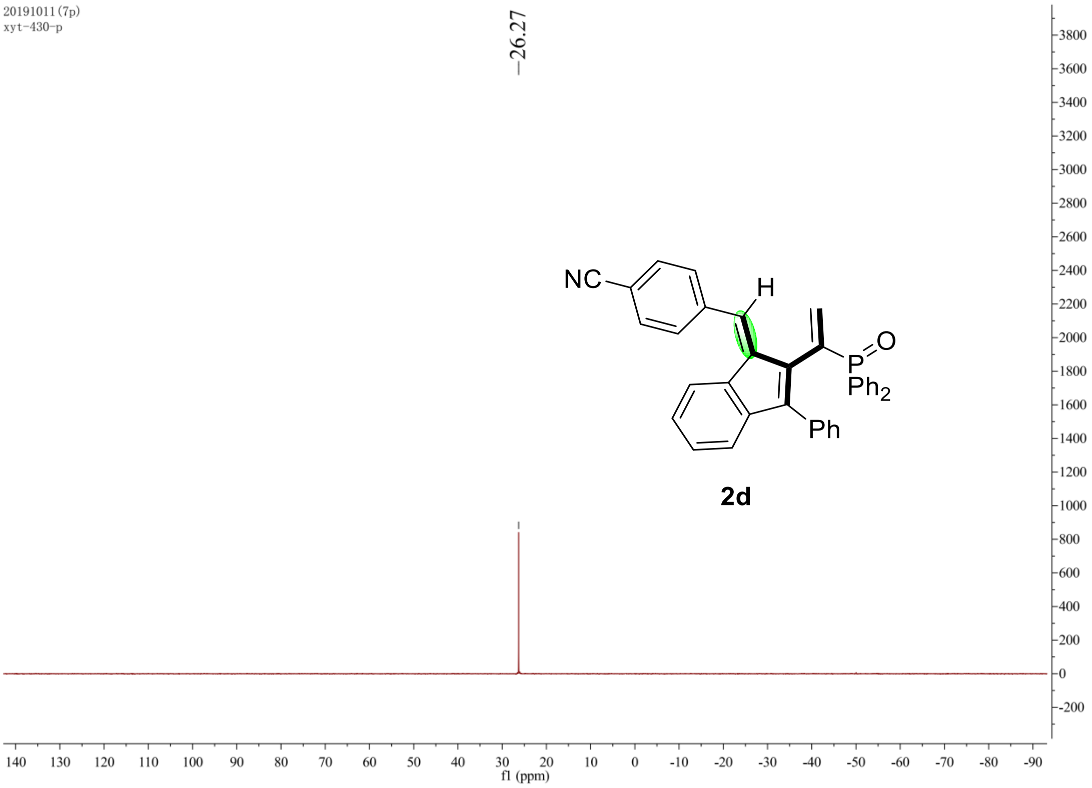
20191011(7p)
xyt-430-p
26.27
NC
H
P
O
Ph2
Ph
2d
f1 (ppm)

2e

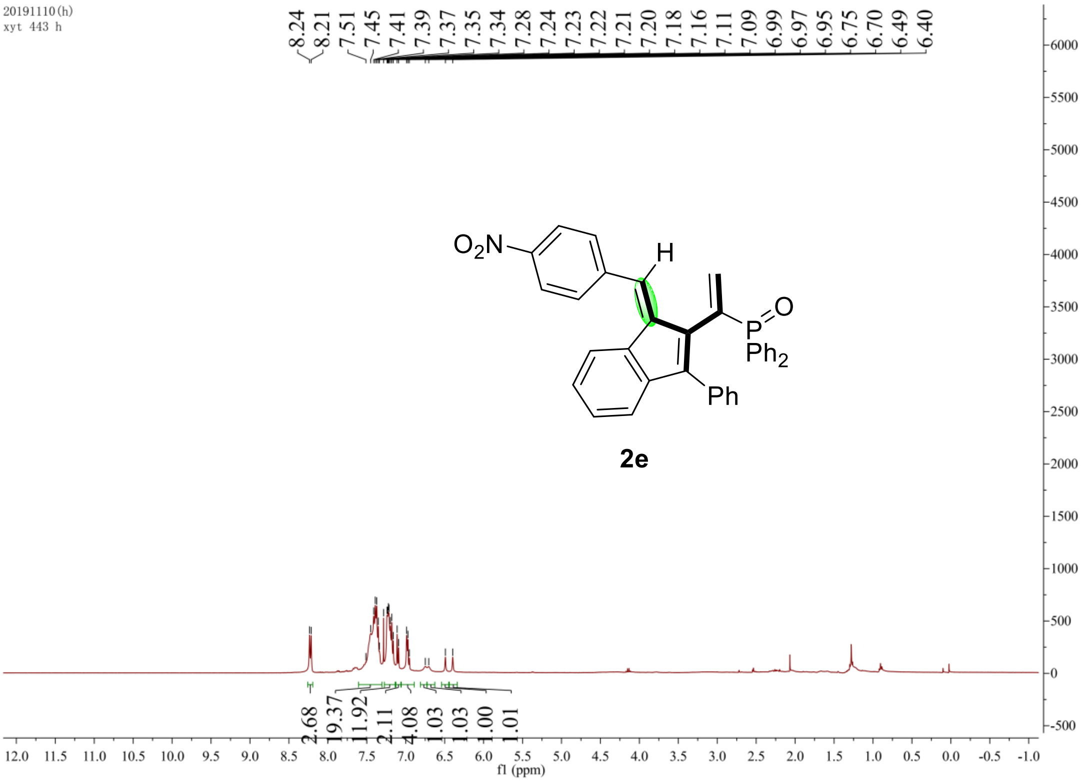
20191110(h)
xyt 443 h
8.24
8.21
7.51
7.45
7.41
7.39
7.37
7.35
7.34
7.28
7.24
7.23
7.22
7.21
7.20
7.18
7.16
7.11
7.09
6.99
6.97
6.95
6.75
6.70
6.49
6.40
O2N
H
P=O
Ph2
Ph
2e
2.68
19.37
11.92
2.11
4.08
1.03
1.03
1.00
1.01
f1 (ppm)

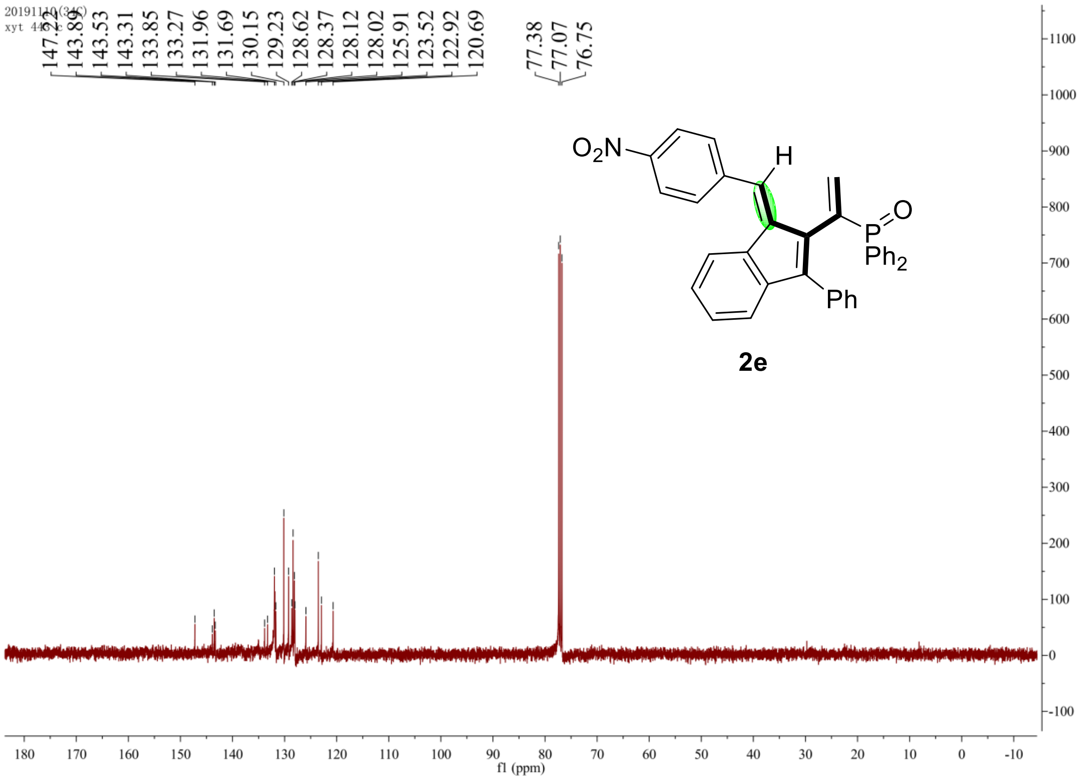
20191110(13C)
147.72
143.89
143.53
143.31
133.85
133.27
131.96
131.69
130.15
129.23
128.62
128.37
128.12
128.02
125.91
123.52
122.92
120.69
77.38
77.07
76.75
O2N
H
P=O
Ph2
Ph
2e
f1 (ppm)

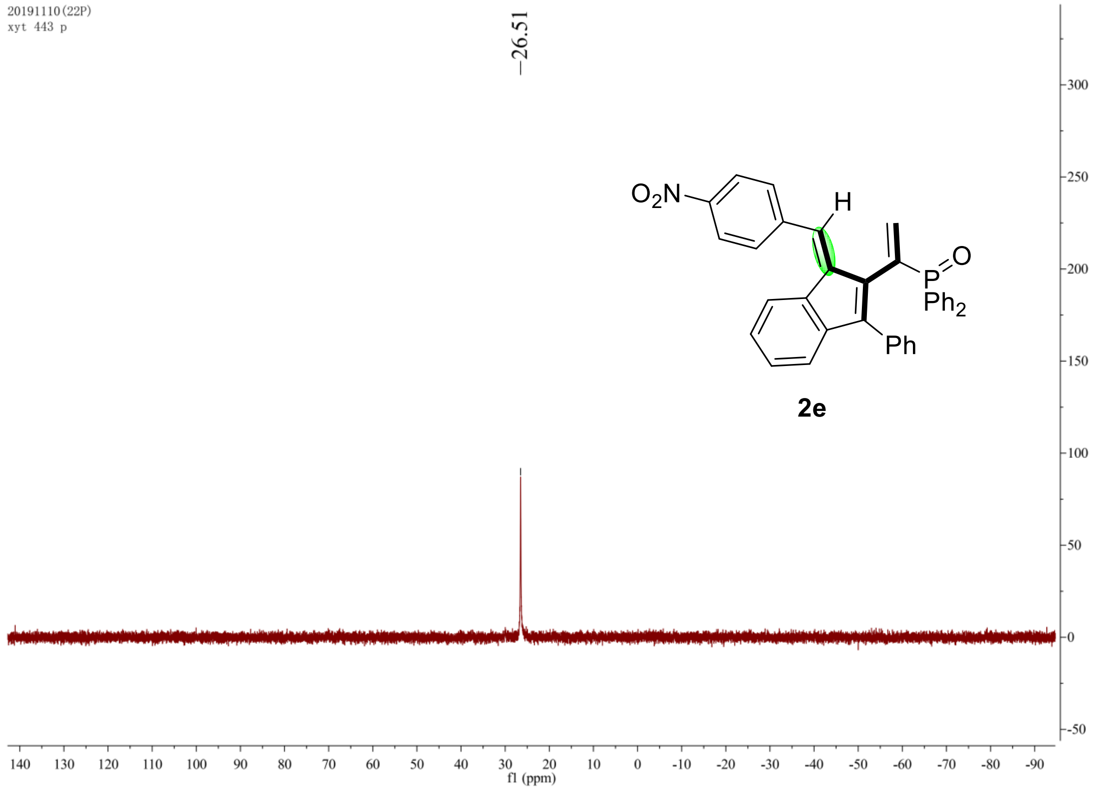
20191110(22P)
xyt 443 p
—26.51
O_2N
H
P=O
Ph_2
Ph
2e
f1 (ppm)

2f

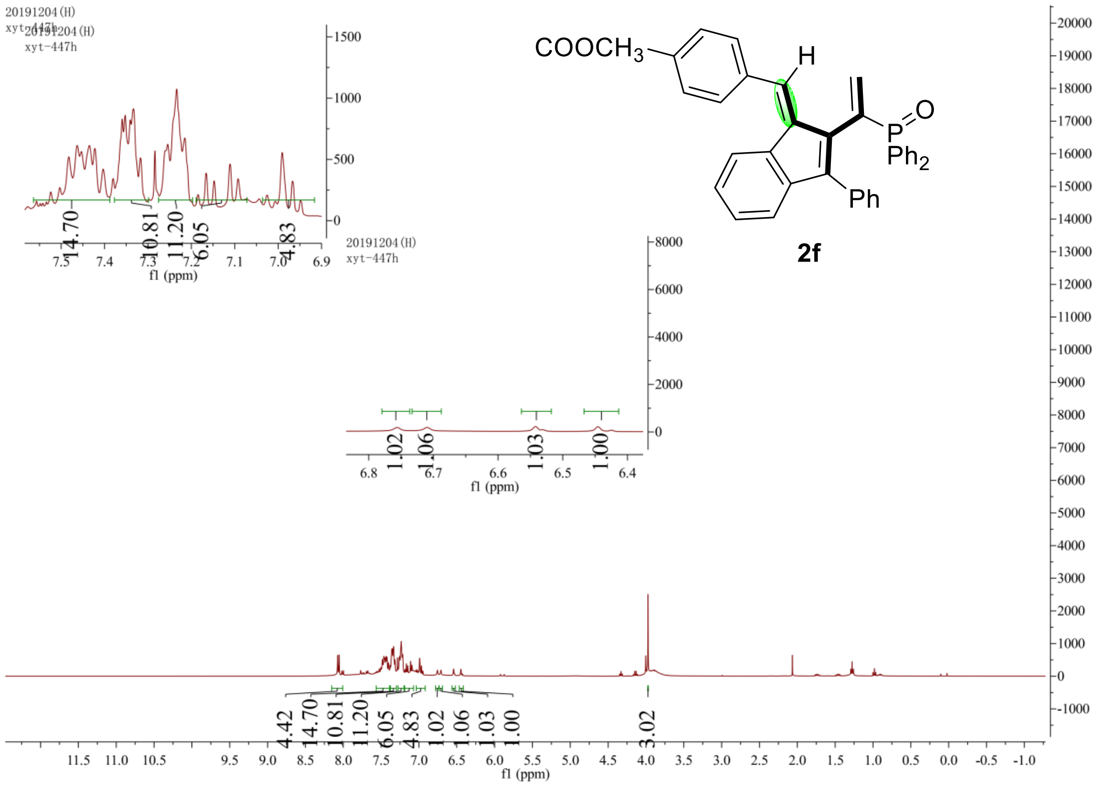
20191204(H)
xyt-447h
COOCH$_3$
H
P=O
Ph_2
Ph
2f
14.70
10.81
11.20
6.05
4.83
1.02
1.06
1.03
1.00
4.42
3.02
f1 (ppm)

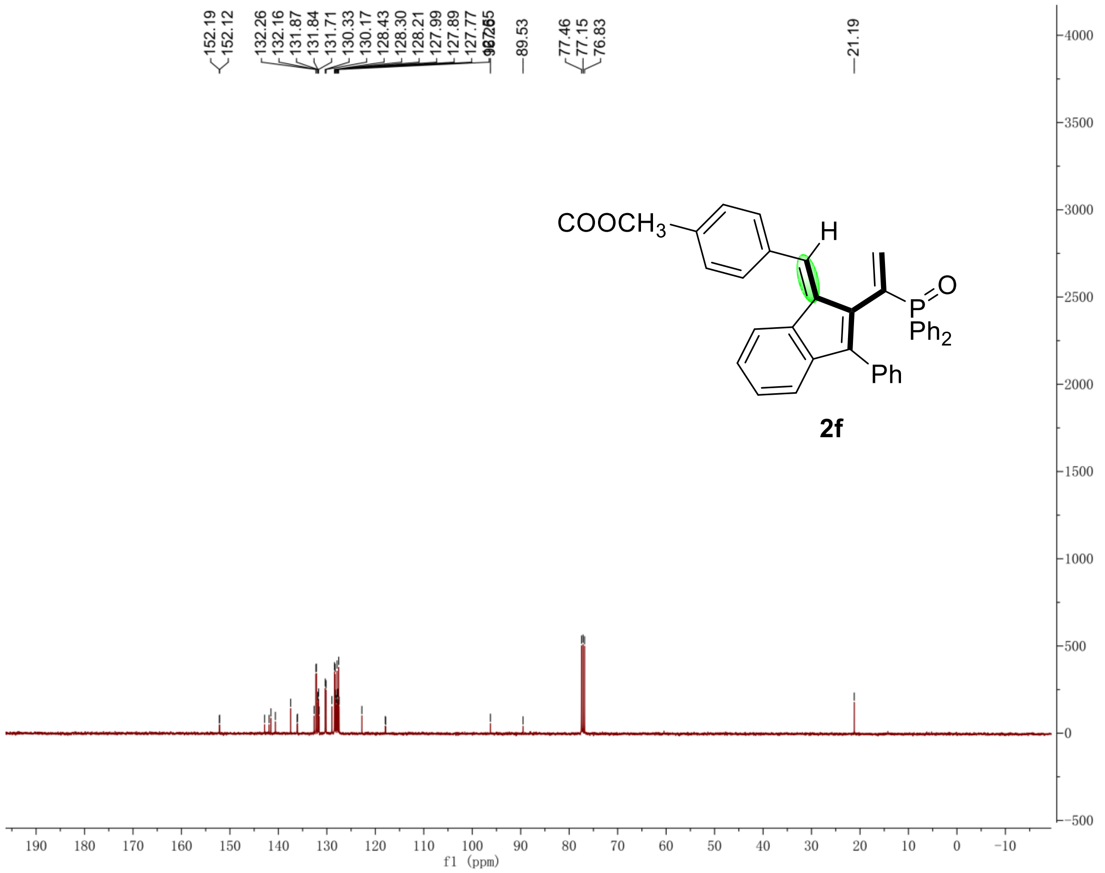
152.19
152.12
132.26
132.16
131.87
131.84
131.71
130.33
130.17
128.43
128.30
128.21
127.99
127.89
127.77
89.53
77.46
77.15
76.83
21.19
COOCH3
H
P=O
Ph2
Ph
2f
f1 (ppm)

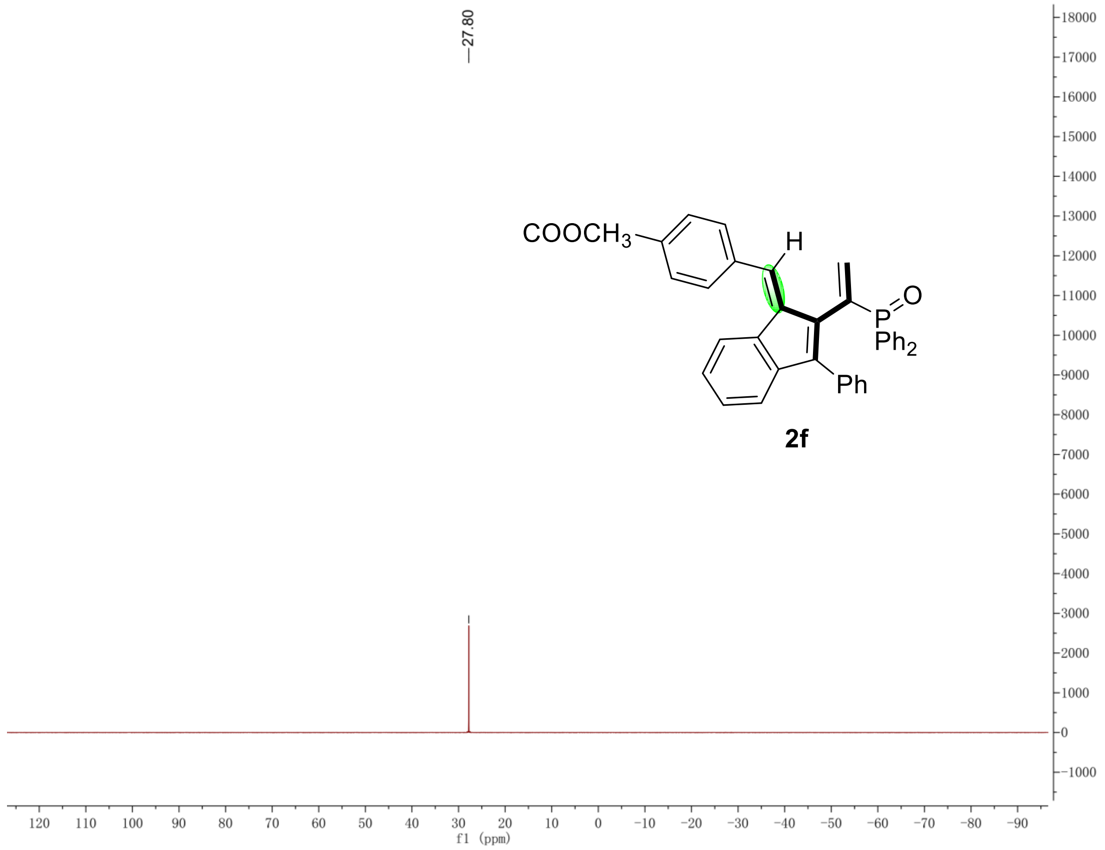
27.80
COOCH3
H
P=O
Ph2
Ph
2f
f1 (ppm)

3g

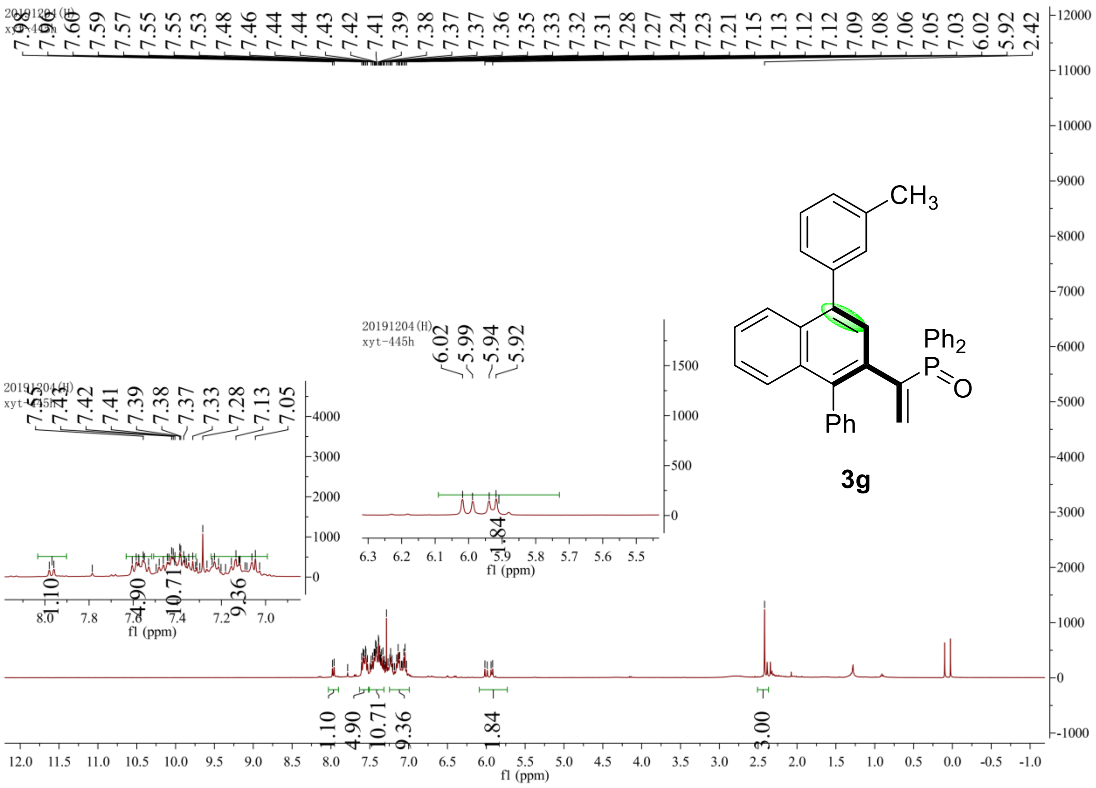

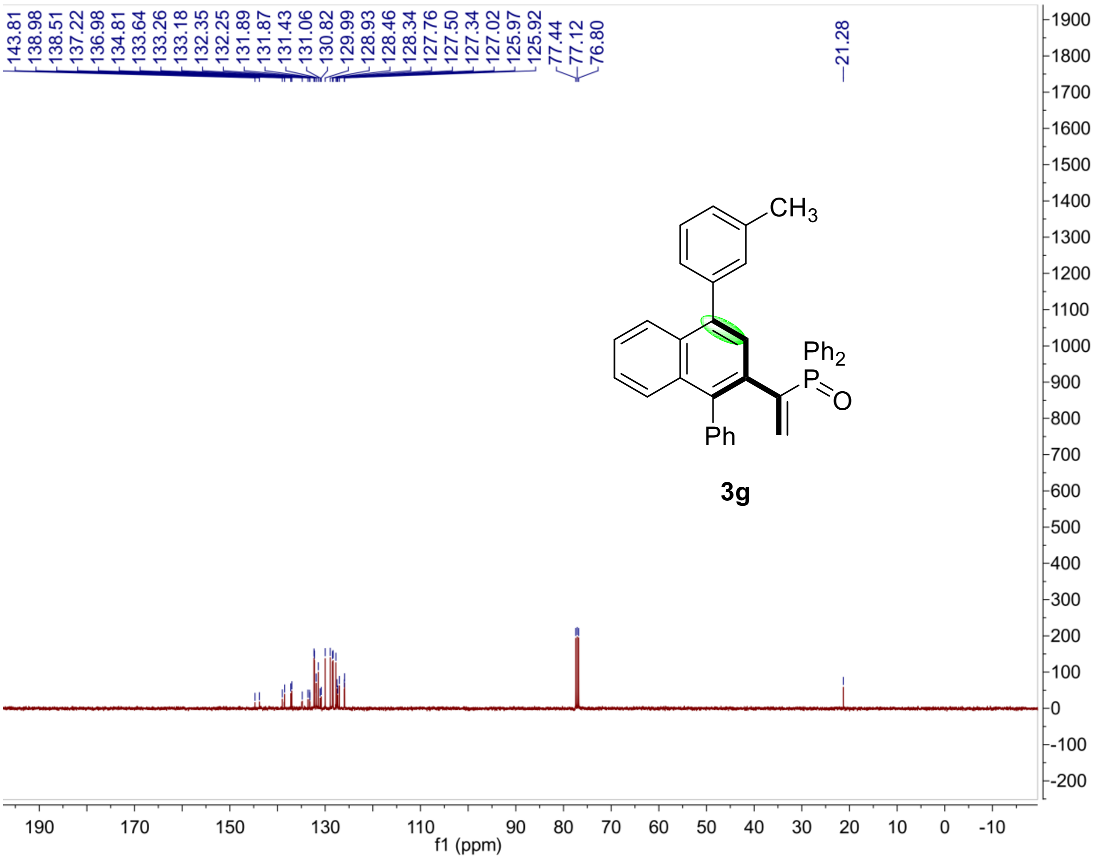

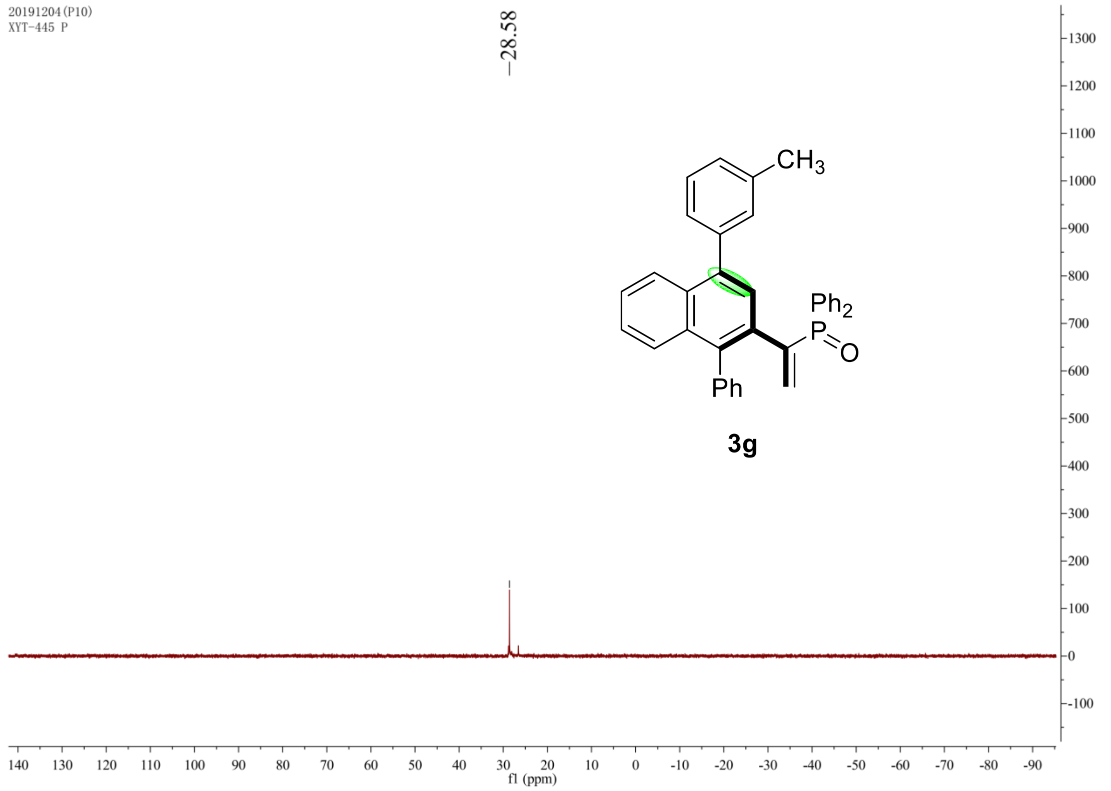
20191204(P10)
XYT-445 P
28.58
CH3
Ph2
P
O
Ph
3g
f1 (ppm)

3h

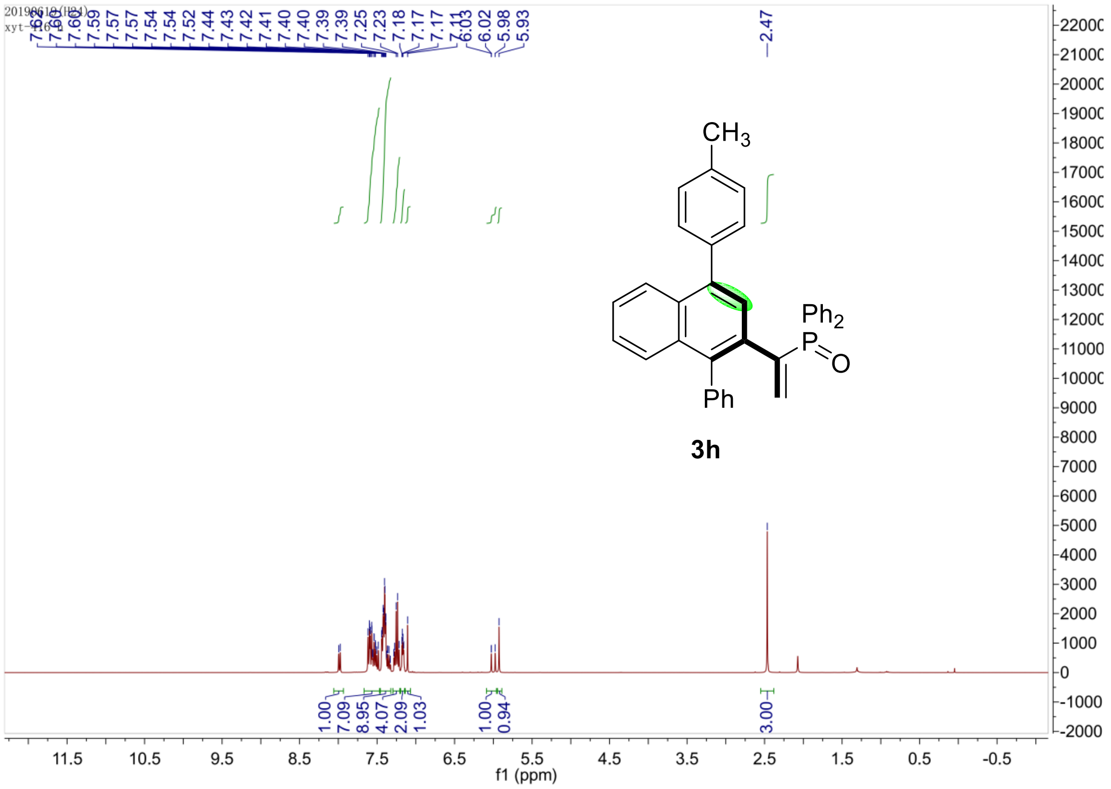
7.62 7.60 7.60 7.59 7.57 7.57 7.54 7.54 7.52 7.44 7.43 7.42 7.41 7.40 7.39 7.39 7.25 7.23 7.18 7.17 7.17 7.11 6.03 6.02 5.98 5.93
2.47
CH3
Ph2
P
O
Ph
3h
1.00 7.09 8.95 4.07 2.09 1.03
1.00 0.94
3.00
f1 (ppm)

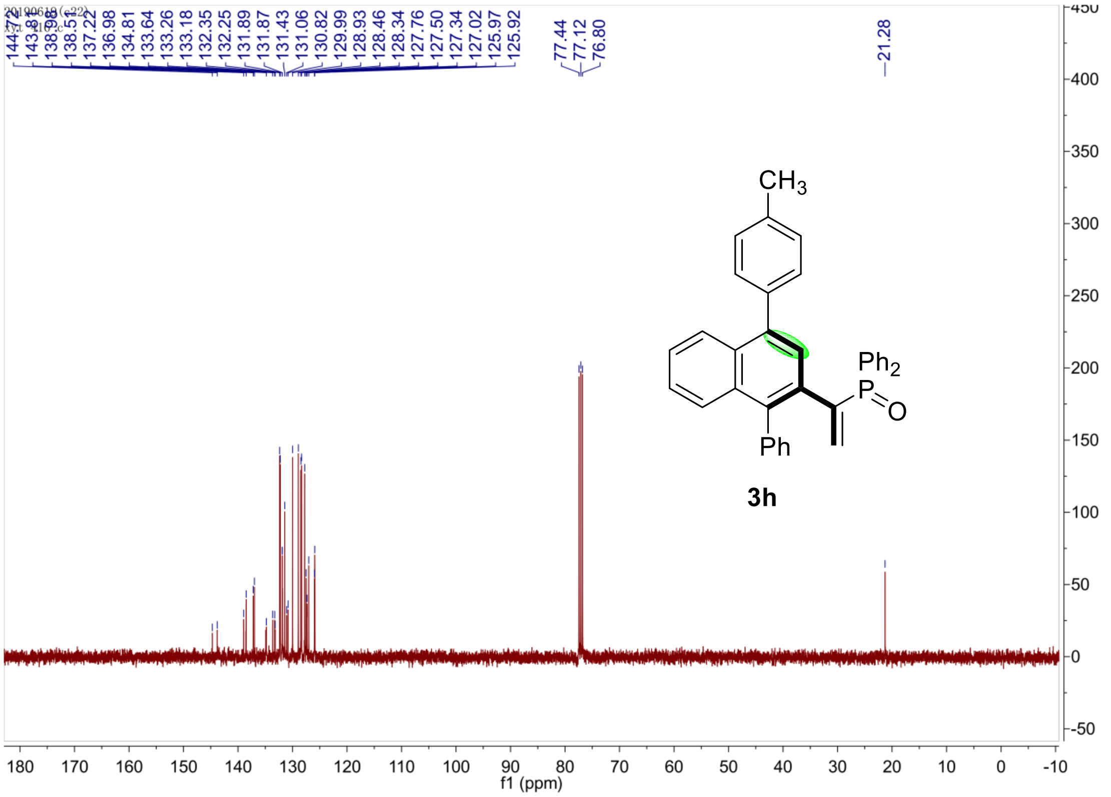
20190619(c22)
xyt416-c
144.72
143.81
138.98
138.51
137.22
136.98
134.81
133.64
133.26
133.18
132.35
132.25
131.89
131.87
131.43
131.06
130.82
129.99
128.93
128.46
128.34
127.76
127.50
127.34
127.02
125.97
125.92
77.44
77.12
76.80
21.28
CH3
Ph2
P
O
Ph
3h
f1 (ppm)

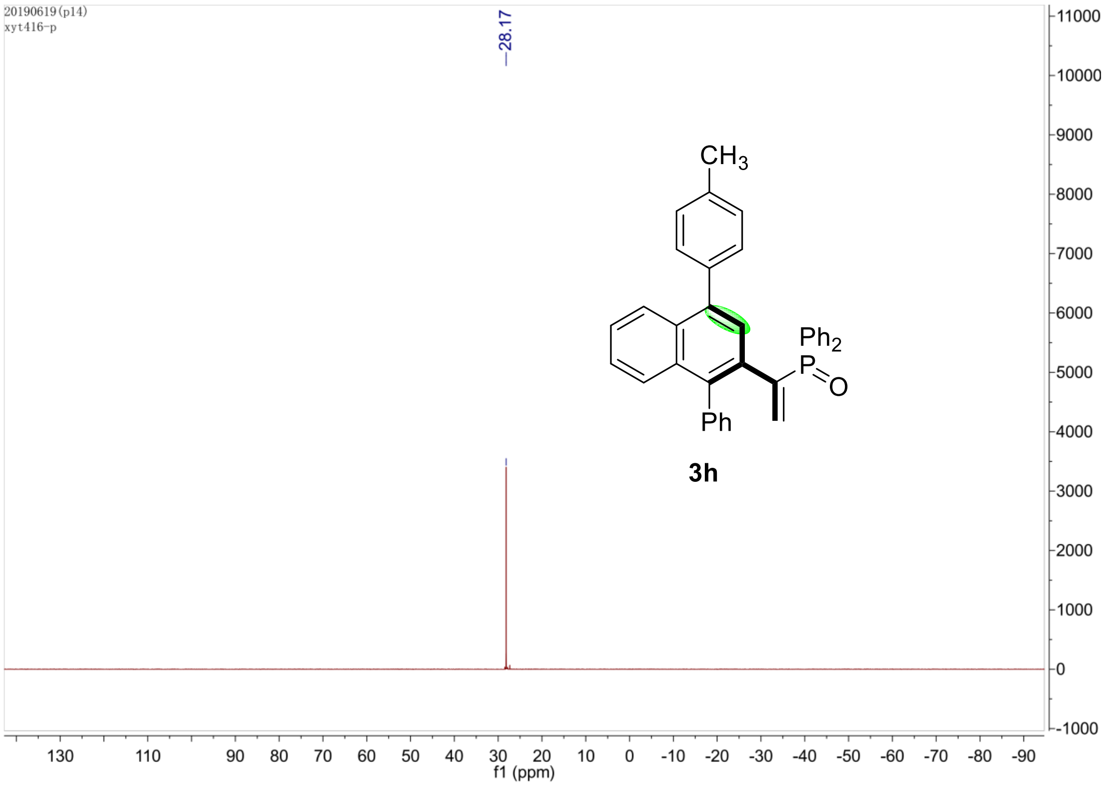
20190619(p14)
xyt416-p
28.17
CH3
Ph2
P
O
Ph
3h
f1 (ppm)

3i

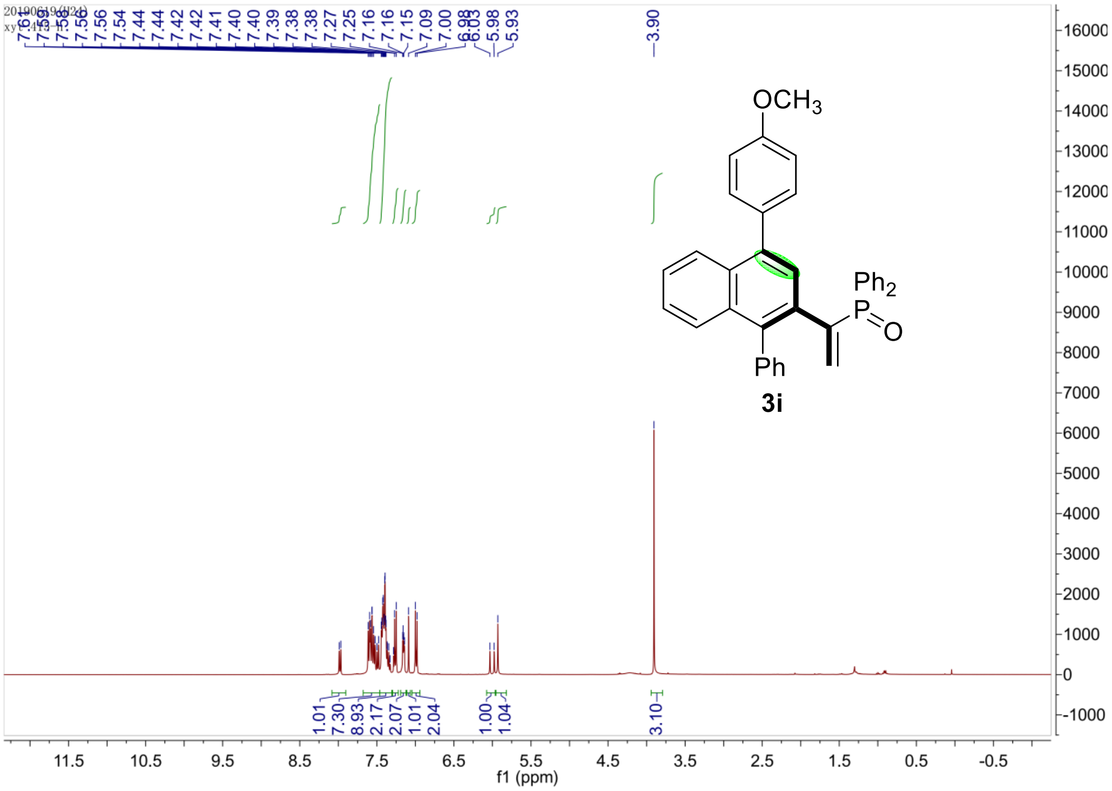

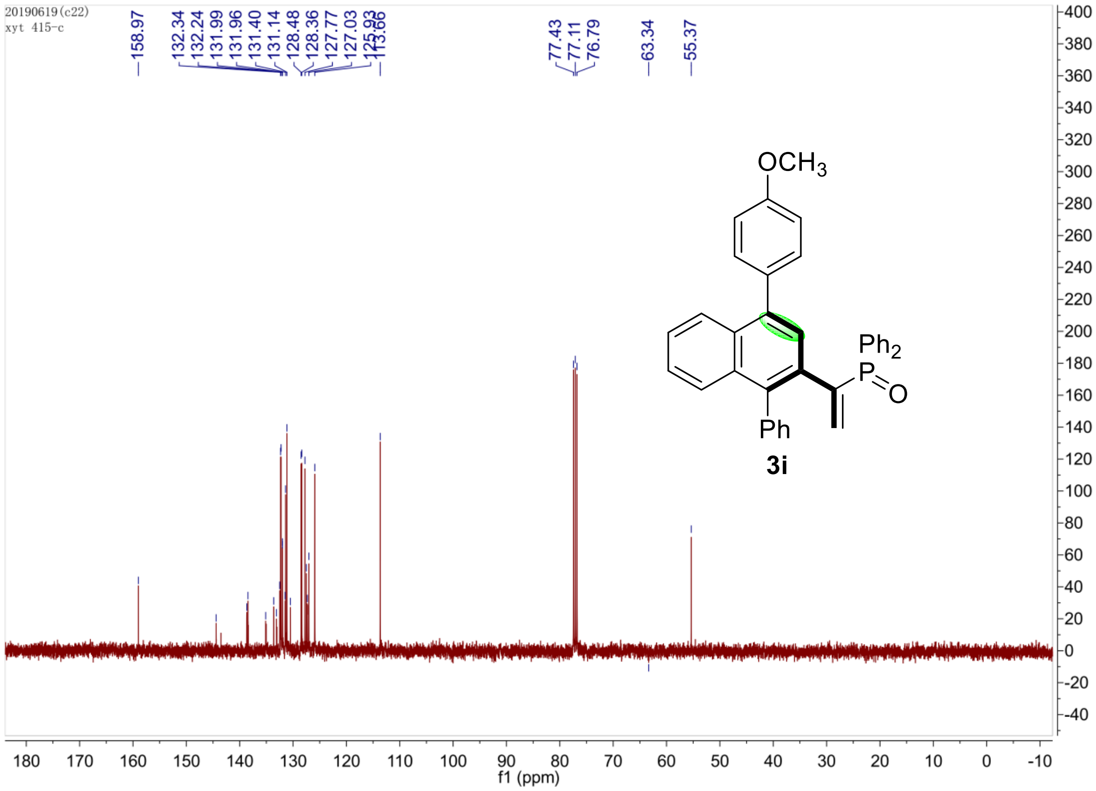

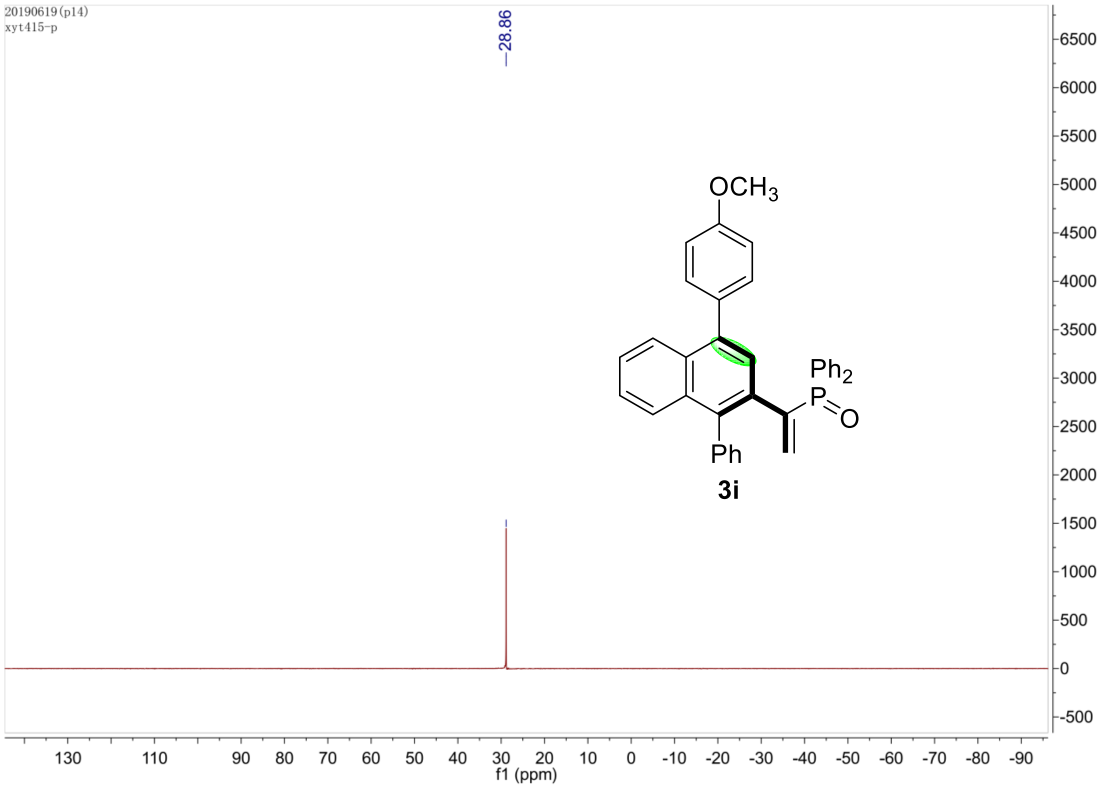
20190619(p14)
xyt415-p
—28.86
OCH3
Ph2
P
O
Ph
3i
f1 (ppm)

3k

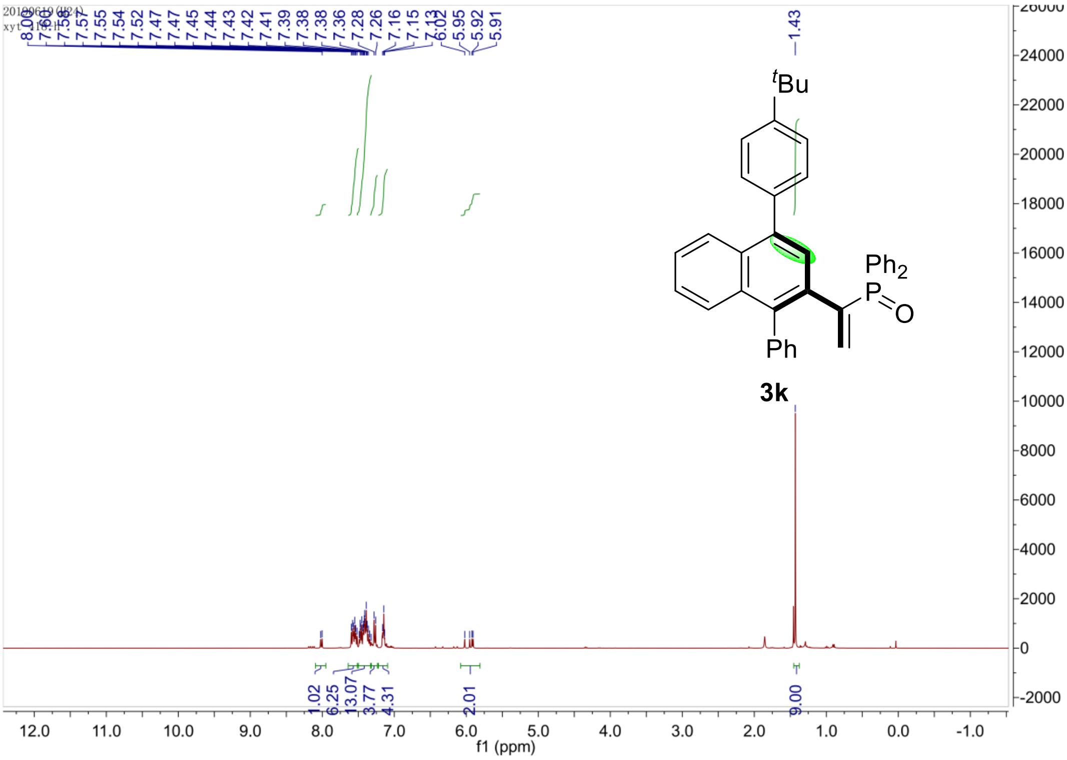
—1.43
tBu
Ph2
P
O
Ph
3k
1.02
6.25
13.07
3.77
4.31
2.01
9.00
f1 (ppm)

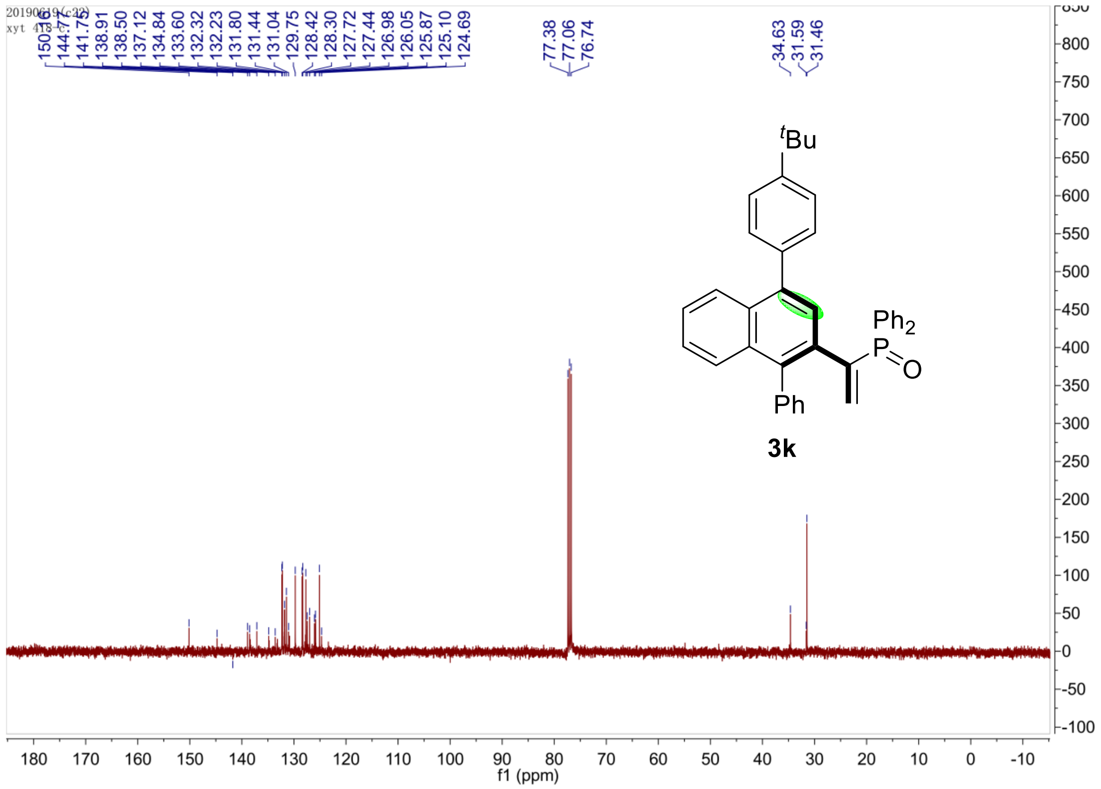
141.75
138.91
138.50
137.12
134.84
133.60
132.32
132.23
131.80
131.44
131.04
129.75
128.42
128.30
127.72
127.44
126.98
126.05
125.87
125.10
124.69
77.38
77.06
76.74
34.63
31.59
31.46
f1 (ppm)
tBu
Ph2
P
O
Ph
3k

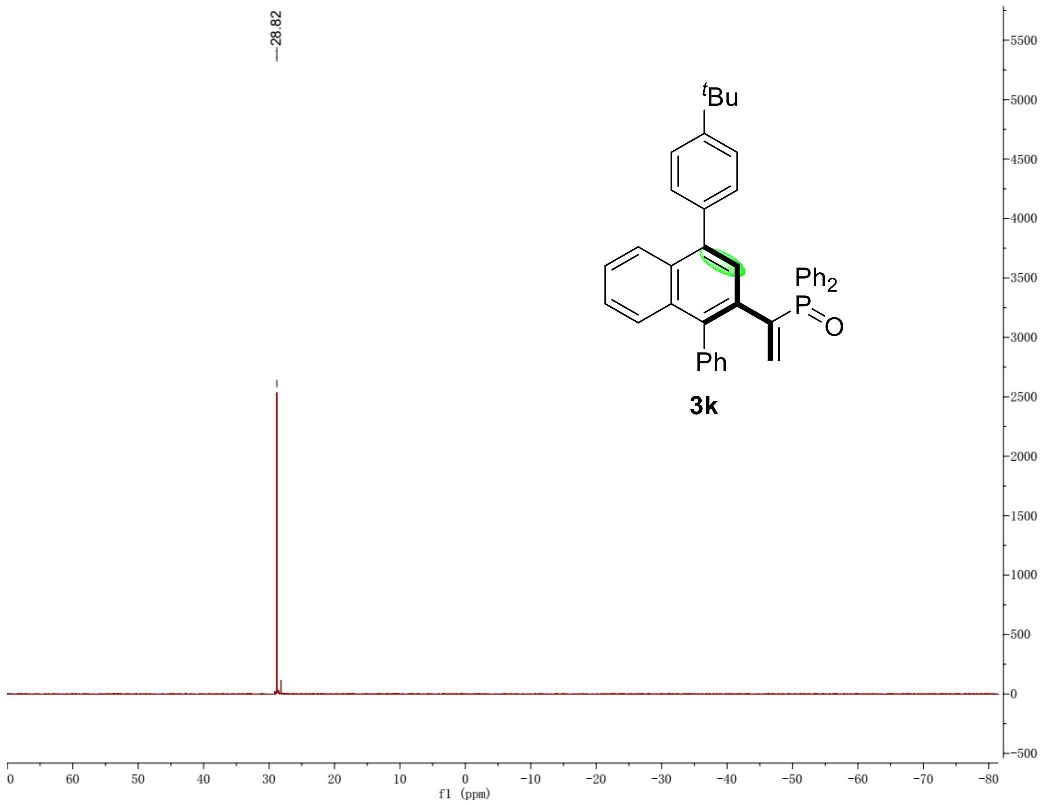
28.82
f1 (ppm)
tBu
Ph2
P
O
Ph
3k

3l

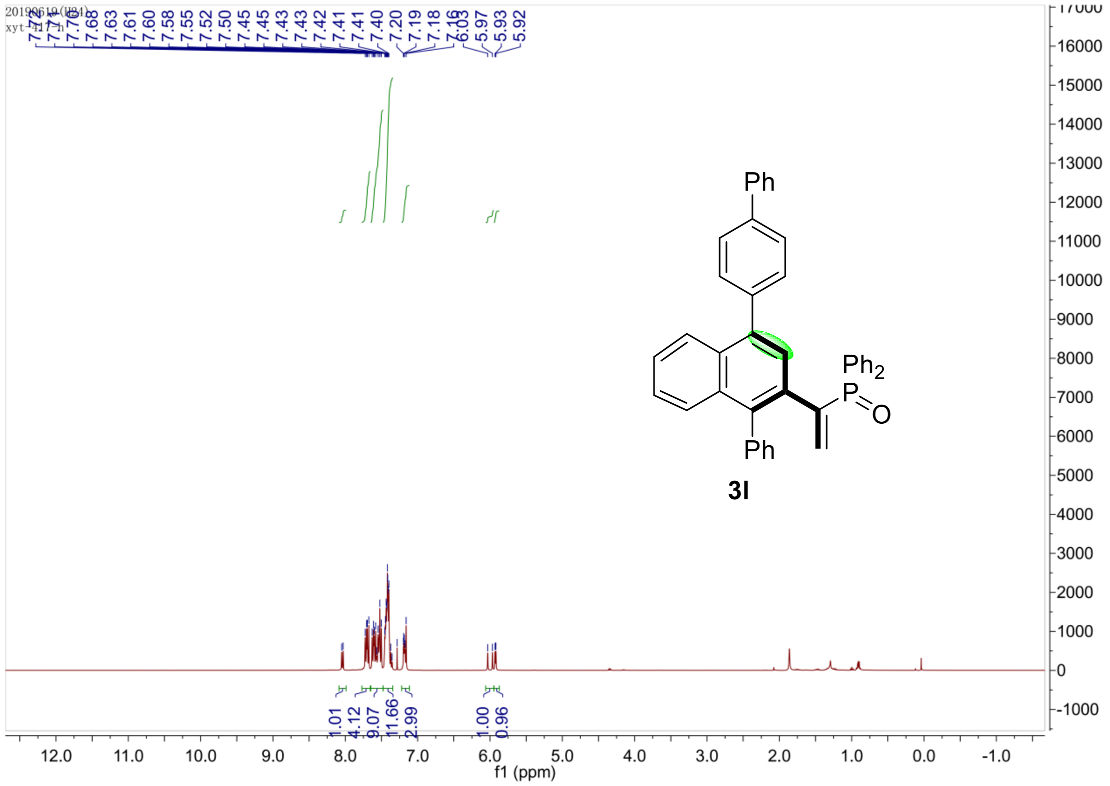

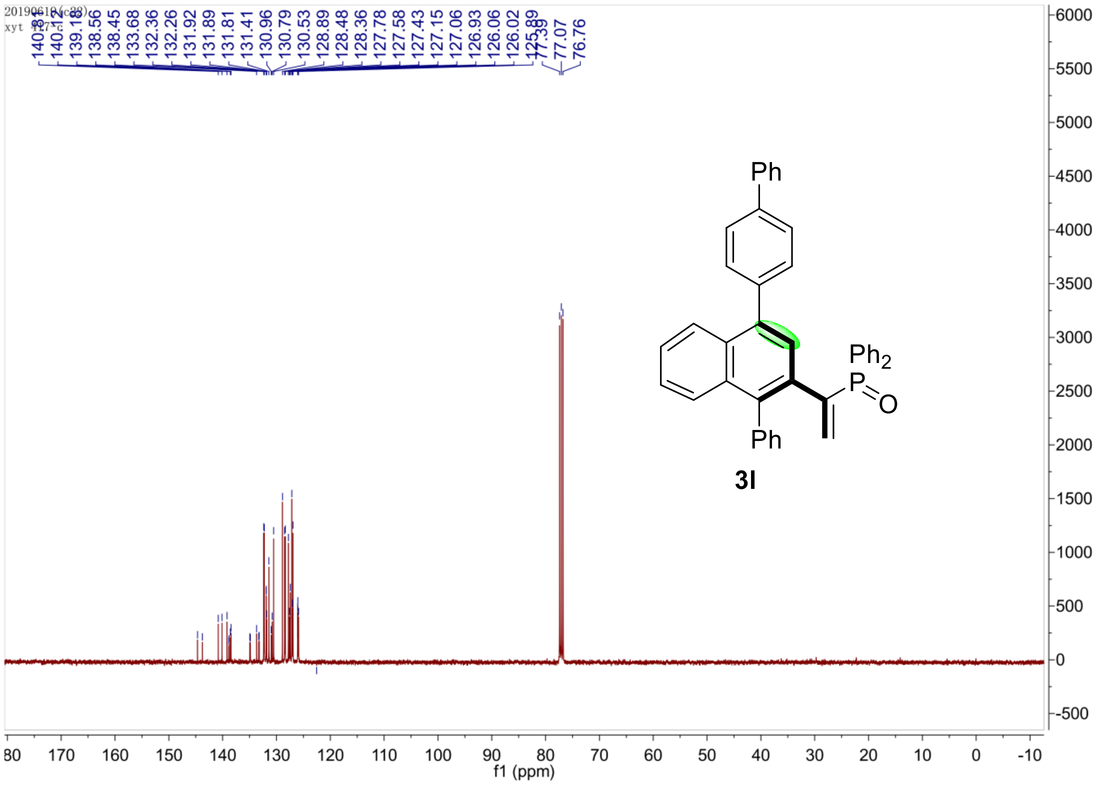

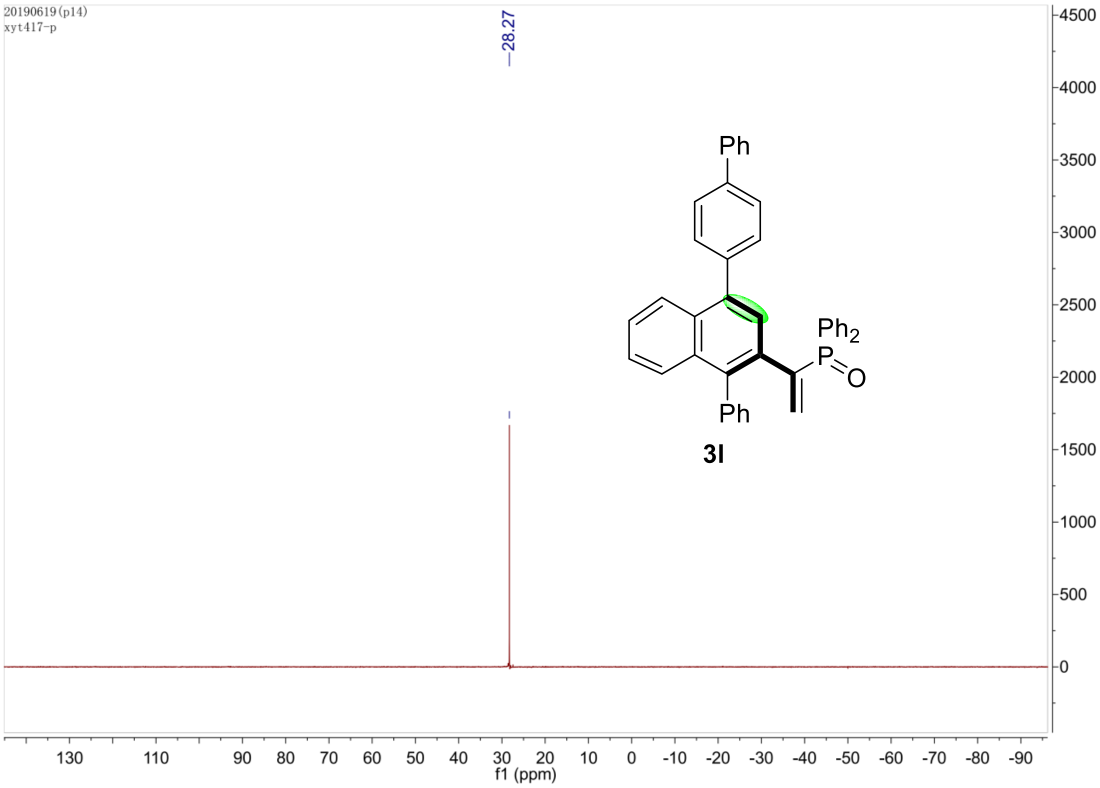
20190619(p14)
xyt417-p
28.27
Ph
Ph2
P
O
Ph
3l
f1 (ppm)

3m

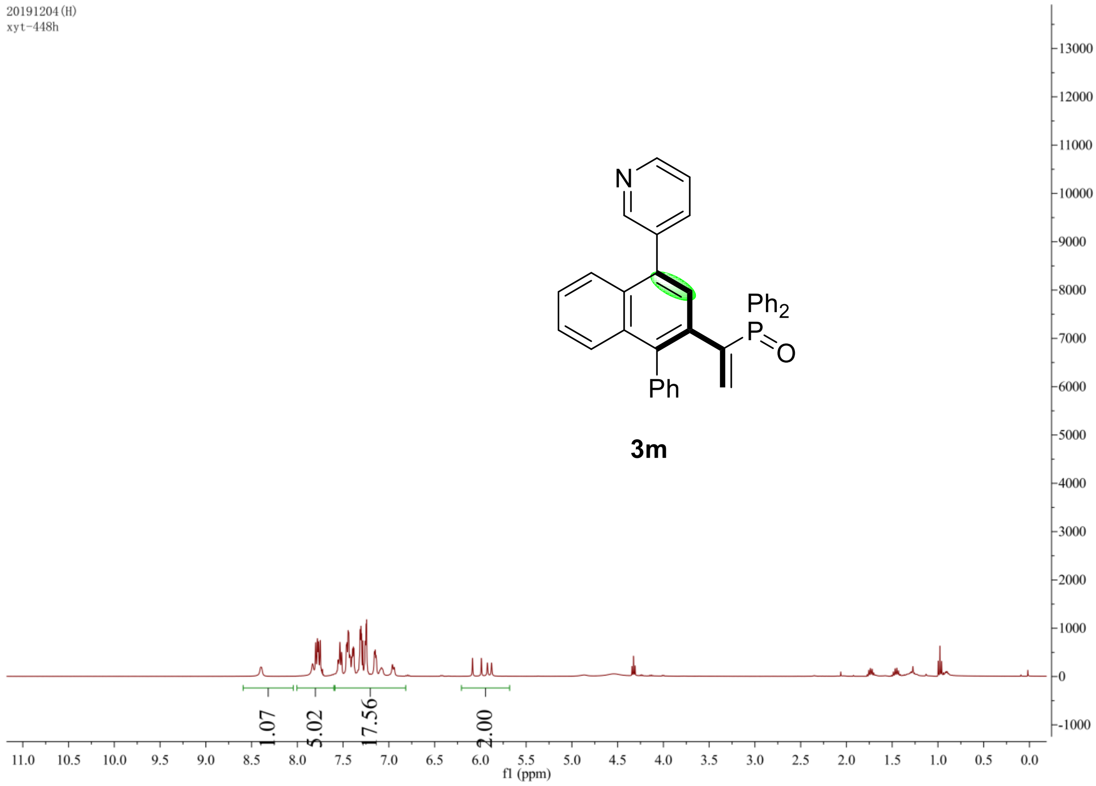
20191204(H)
xyt-448h
N
Ph2
P
O
Ph
3m
1.07
5.02
17.56
2.00
f1 (ppm)

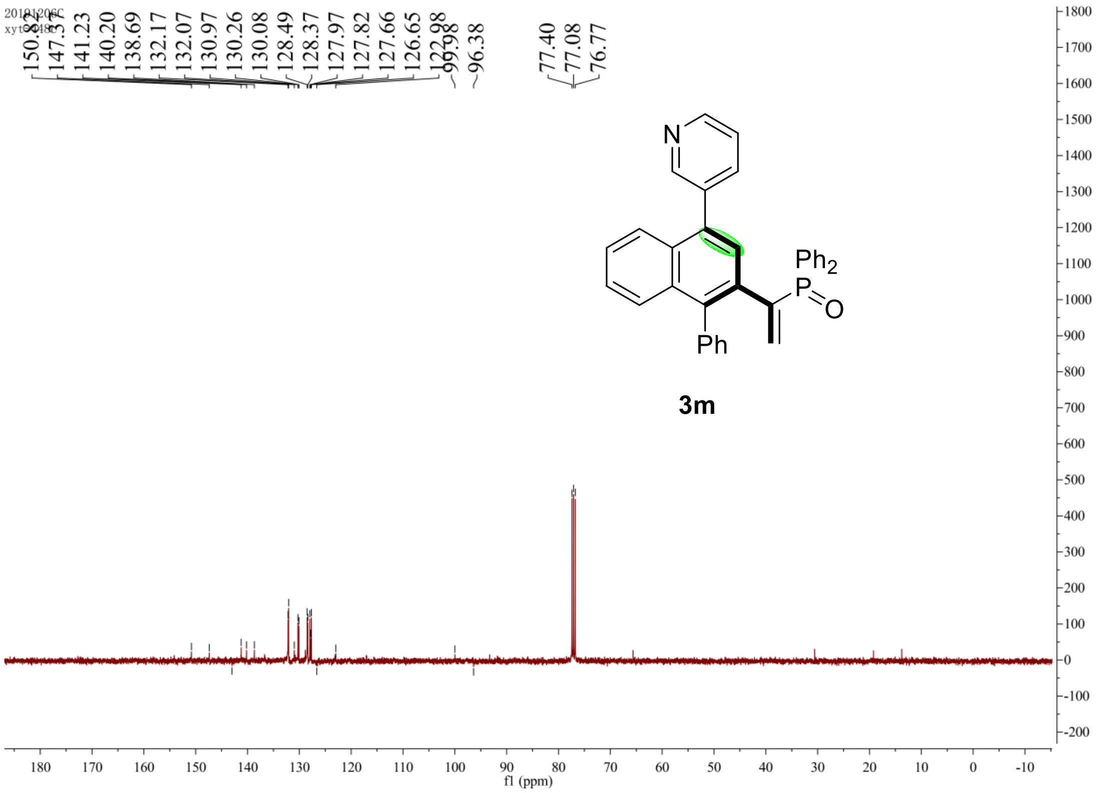
150.82
147.57
141.23
140.20
138.69
132.17
132.07
130.97
130.26
130.08
128.49
128.37
127.97
127.82
127.66
126.65
122.98
99.98
96.38
77.40
77.08
76.77
N
Ph2
P
O
Ph
3m
f1 (ppm)

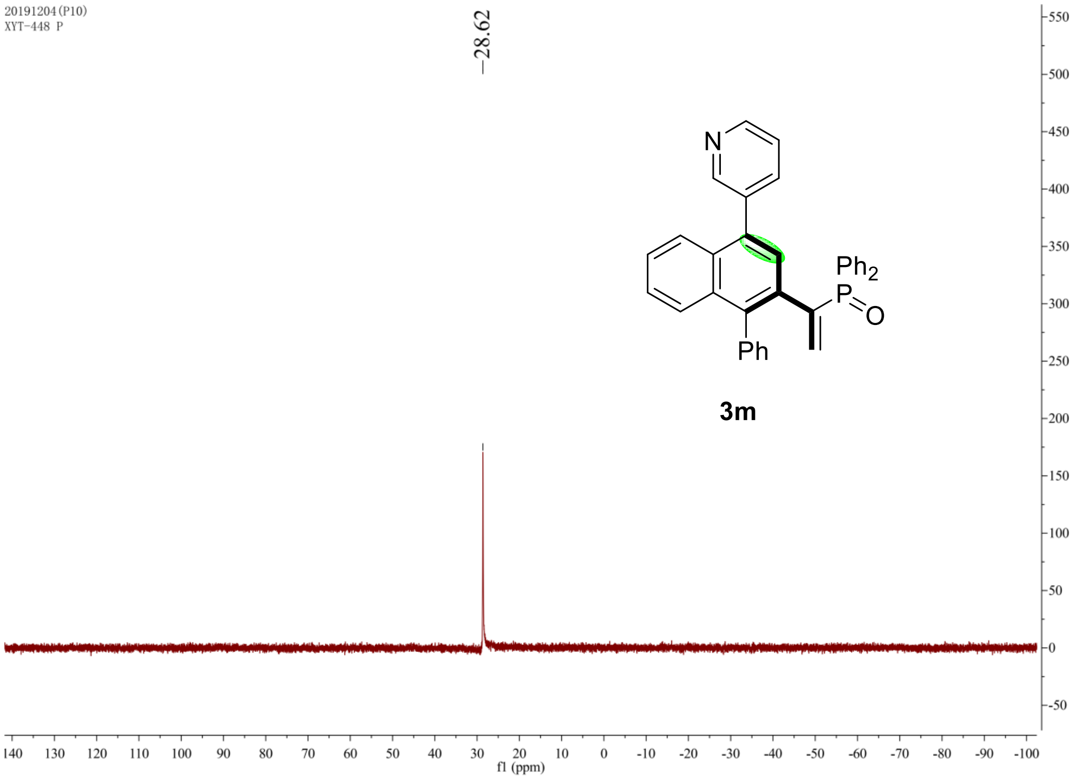
20191204(P10)
XYT-448 P
28.62
N
Ph2
P
O
Ph
3m
f1 (ppm)

3n

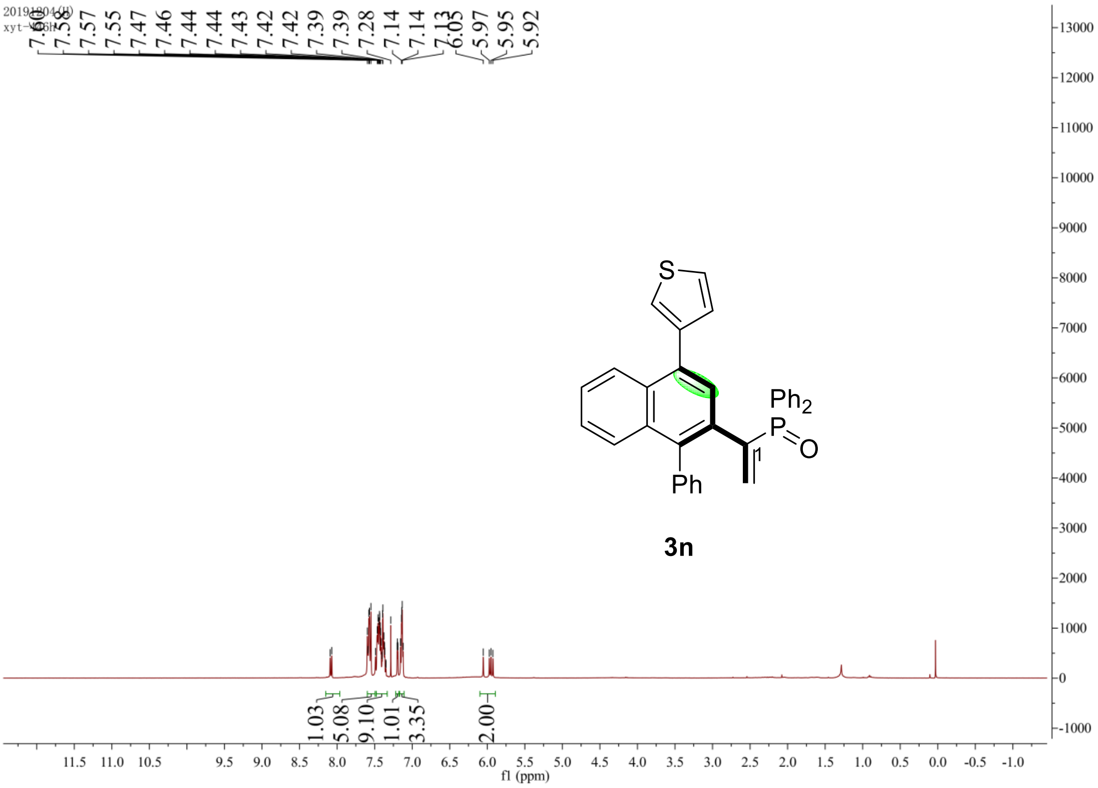
7.60
7.58
7.57
7.55
7.47
7.46
7.44
7.44
7.43
7.42
7.42
7.39
7.39
7.28
7.14
7.14
7.13
6.05
5.97
5.95
5.92
S
Ph2
P
O
Ph
3n
1.03
5.08
9.10
1.01
3.35
2.00
fl (ppm)

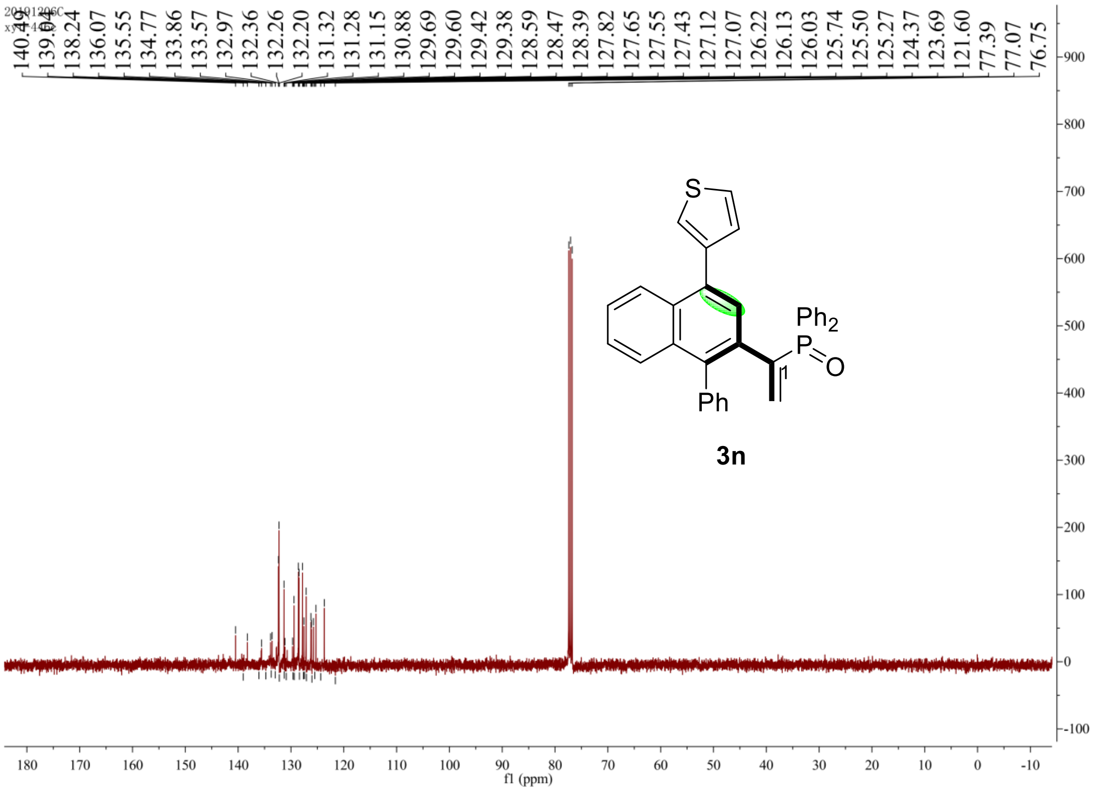
140.49
139.04
138.24
136.07
135.55
134.77
133.86
133.57
132.97
132.36
132.26
132.20
131.32
131.28
131.15
130.88
129.69
129.60
129.42
129.38
128.59
128.47
128.39
127.82
127.65
127.55
127.43
127.12
127.07
126.22
126.13
126.03
125.74
125.50
125.27
124.37
123.69
121.60
77.39
77.07
76.75
S
Ph2
P
O
Ph
3n
fl (ppm)

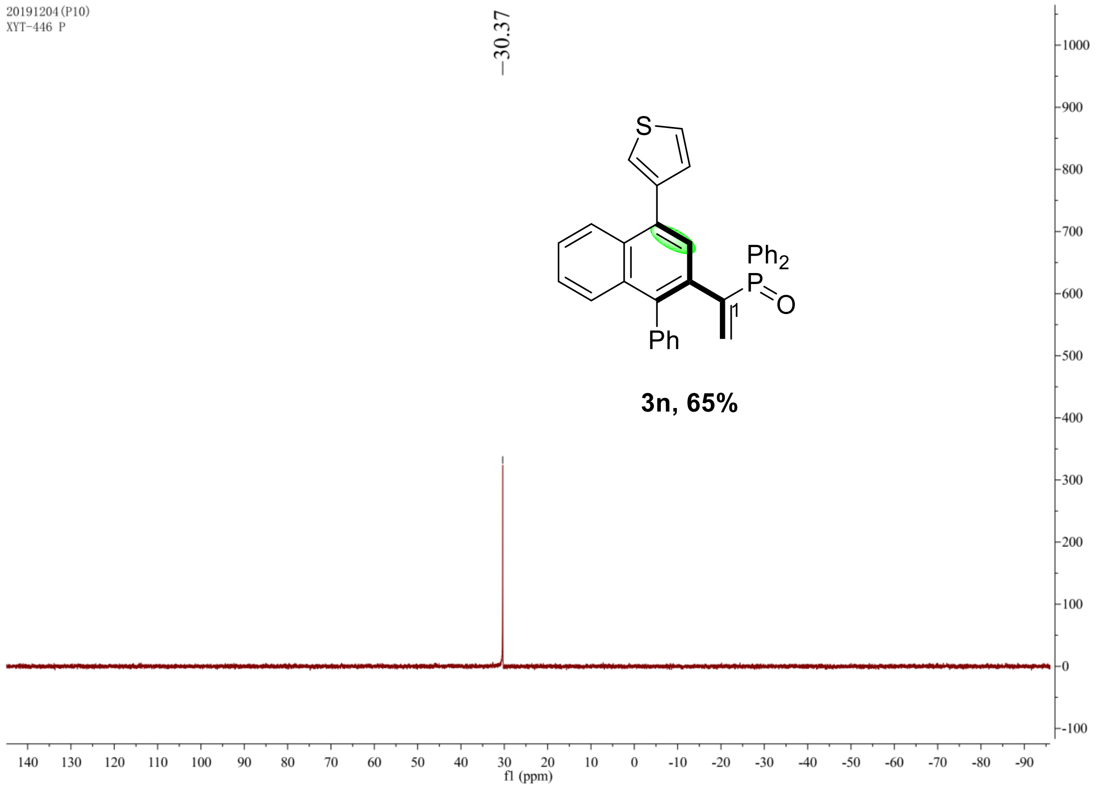
20191204(P10)
XYT-446 P
—30.37
S
Ph2
P
O
Ph
3n, 65%
f1 (ppm)
